# Baseline Body Mass Index and the Efficacy of Hypoglycemic Treatment in Type 2 Diabetes: A Meta-Analysis

**DOI:** 10.1371/journal.pone.0166625

**Published:** 2016-12-09

**Authors:** Xiaoling Cai, Wenjia Yang, Xueying Gao, Lingli Zhou, Xueyao Han, Linong Ji

**Affiliations:** Peking University People’s Hospital, Endocrine & Metabolism Department, Beijing, China; Universita degli Studi di Bari Aldo Moro, ITALY

## Abstract

**Aim:**

The aim of this study is to compare the effects of hypoglycemic treatments in groups of patients categorized according to the mean baseline body mass indexes (BMIs).

**Methods:**

Studies were identified by a literature search and all the studies were double blind, placebo-controlled randomized trials in type 2 diabetes patients; study length of ≥12 weeks with the efficacy evaluated by changes in HbA1c from baseline in groups. The electronic search was first conducted in January 2015 and repeated in June 2015.

**Results:**

227 studies were included. Treatment with sulfonylureas was compared with placebo in overweight patients and resulted in a significantly greater change in the HbA1c levels (weighted mean difference (WMD), −1.39%) compared to obese patients (WMD, −0.77%)(p<0.05). Treatment with metformin in overweight patients resulted in a comparable change in the HbA1c levels (WMD, −0.99%) compared to obese patients (WMD, −1.06%)(p>0.05). Treatment with alpha glucosidase inhibitors in normal weight patients was associated with a HbA1c change (WMD, −0.94%) that was comparable that in overweight (WMD, −0.72%) and obese patients (WMD, −0.56%)(p>0.05). Treatment with thiazolidinediones in normal weight patients was associated with a HbA1c change (WMD, −1.04%) that was comparable with that in overweight (WMD, −1.02%) and obese patients (WMD, −0.88%)(p>0.05). Treatment with DPP-4 inhibitors in normal weight patients was associated with a HbA1c change (WMD, −0.93%) that was comparable with that in overweight (WMD, −0.66%) and obese patients (WMD, −0.61%)(p>0.05). In total, of the seven hypoglycemic agents, regression analysis indicated that the mean baseline BMI was not associated with the mean HbA1c changes from baseline.

**Conclusion:**

In each kind of hypoglycemic therapy in type 2 diabetes, the baseline BMI was not associated with the efficacy of HbA1c changes from baseline.

## Introduction

The efficacy of glucose lowering effects of different hypoglycemic drugs is well known; however, in obese or overweight people, are the effects on the hemoglobin A1c (HbA1c) change comparable with normal weight people? There is uncertainty regarding whether treatment with hypoglycemic drugs is different in patients with different body mass indexes (BMIs), which might depend on the choice of drug. Some investigators performed a series of randomized clinical trials and post-hoc analyses comparing the effects of glucose lowering drugs at different BMI levels and had inconsistent findings. In the ADOPT study [1], the subgroup analyses for different baseline BMI levels suggested that the treatment effect was significantly greater with rosiglitazone than with glyburide for obese patients (>30 kg/m^2^) compared to overweight patients (≤30 kg/m^2^). The post-hoc analysis of ADVANCE study [2,3] indicated that one of the independent predictors of change in HbA1c with gliclazide MR was baseline BMI (p < 0.001). In a group of Korean type 2 diabetes patients [4], one of the predictors of good response to metformin was higher BMI. In the same group of patients, they also found that the predictor of good response to rosiglitazone was higher BMI. In extremely obese Caucasians [5], relatively lower BMI (31 kg/m^2^ versus 37 kg/m^2^) was reported as the predictor of good response to thiazolidinediones (TZDs). In a study of Japanese type 2 diabetes patients with sitagliptin treatment [6], multiple regression analysis indicated that baseline BMI was independently correlated with HbA1c reduction at 3 months (p < 0.001). Contrarily, in a trial [7] comparing the efficacy of metformin monotherapy among normal-weight, overweight, and obese patients with newly diagnosed type 2 diabetes, Ji reported that baseline BMI had no impact on glycemic control. Additionally, some meta-analyses [8] indicated that baseline BMI might be associated with the different efficacies of glucose changes for some hypoglycemic treatments, while others did not [9,10]. Moreover, in a recently published review [11], the authors indicated that the shared identified common variants of type 2 diabetes and obesity was limited. Therefore, because the association between baseline BMI and treatment efficacy has not been evaluated comprehensively, the aim of this meta-analysis is to compare the effects of blood glucose lowering regimens in groups of type 2 diabetes patients who are categorized by baseline BMI.

## Materials and Methods

### Search strategy

Studies were identified by a literature search of MEDLINE^®^ (PubMed), EMBASE^®^ and the Cochrane Central Register of Controlled Trials (CENTRAL) from when recording began until December 2014. The electronic search was first conducted in January 2015 and repeated in June 2015. The overall strategy was performed using the following terms: type 2 diabetes; metformin; sulfonylurea; alpha glucosidase inhibitors; thiazolidinediones; DPP-4 inhibitors; sodium-glucose cotransporter 2 inhibitors; glucagon-like peptide-1; incretin; and randomized controlled trials. The PubMed search strategy formed the basis for the strategies developed for the other electronic databases. Moreover, documents for approved medications were searched for trials at the clinical trials website (http://www.clinicalstudyresults.org and http://www.clinicaltrials.gov). Results were limited to trials published in English.

The registration number for this meta-analysis is: CRD42015024171.

### Study selection

The inclusion criteria for this meta-analysis were as follows: 1) Placebo-controlled randomized anti-diabetic treatment trial performed in type 2 diabetes participants; 2) study length ≥12 weeks; 3) glucose change was assessed as the change in HbA_1c_ from baseline during the clinical trial in the comparative groups; and 4) baseline BMI was reported in the trial; 5) mono-therapy or add-on therapy.

Two authors (XC and WY) independently evaluated the eligibility of all of the studies retrieved from the databases in duplicate based on predetermined inclusion criteria. Disagreements between reviewers were resolved by consultation with a third investigator (XG). The quality of each study and the risk of bias were evaluated by the Cochrane instrument [12].

### Data extraction

Two review authors (XC and WY) independently extracted the following data from each publication using a standardized form: publication data (title, first author, year and source of publication), study design, baseline characteristics of the study population (sample size, age, duration of T2DM, and HbA_1c_), description of the study drugs, treatment duration, and primary outcome measures (change from baseline to study endpoint for HbA_1c_). Disagreements or discrepancies were resolved by discussion between the two review authors and were also discussed with a third investigator (LZ).

There might be different doses in the seven kinds of non-insulin hypoglycemic treatment, which was documented in the supplement table of baseline characteristics, and was also adjusted as a factor when the meta-regression was made. If there were several doses in one trial, the standard doses recommended and approved in the clinical practice were documented.

### Statistical analysis

BMI was calculated as the weight (kg) divided by the height (m^2^). For each randomized controlled trial, though individual participant data was not provided, the mean baseline BMI of each placebo-controlled hypoglycemic treatment was reported and recorded as a surrogate factor. Studies included in this meta-analysis were divided into the following three groups according to mean baseline BMI: normal weight (BMI<25 kg/m^2^), overweight (25 kg/m^2^≤BMI<30 kg/m^2^), and obese (BMI≥30 kg/m^2^). A meta-analysis was performed in each group that had been stratified by baseline BMI to evaluate the HbA1c changes from baseline with different hypoglycemic treatments.

The statistical analysis has been reported previously (9). All statistical analyses were performed with the Review Manager statistical software package (Version 5.1). This meta-analysis was conducted according to the PRISMA guidelines for the conduct and reporting of meta-analyses of RCTs [13].

Meta-regression was performed for association analysis of the baseline BMI and the efficacy of hypoglycemic treatment, which was adjusted by the baseline age, gender, duration of diabetes, baseline HbA1c as well as study duration. The results were expressed as the p-values for the interaction term (where p <0.05 indicates a significant interaction). Descriptive analysis was used for the demographics and baseline characteristics for each group before hypoglycemic treatment. Meta-regression analyses were performed with the STATA statistical software package (Version 11.0).

There might be different doses in the seven kinds of non-insulin anti-diabetes treatment, which was documented in the supplement table of baseline characteristics, and was also adjusted as a factor when the meta-regression was made. If there were several doses in one trial, the standard doses recommended and approved in the clinical practice were documented (such as metformin 1500-2000mg/day, acarbose 300mg/day, voglibose 0.3mg/day, miglitol 300mg/day, glimepiride 6mg/day, glipiclazide 120mg/day, gliburide 7.5mg/day, glipizide 15mg/day, rosiglitazone 8mg/day, pioglitazone 30mg/day, sitagliptin 100mg/day, vildagliptin 100mg/day, saxagliptin 5mg/day, alogliptin 25mg/day, linagliptin 5mg/day, dapagliflozin 10mg/day, canagliflozin 300mg/day, empagliflozin 25mg/day, ipragliflozin 300mg/day, liraglutide 1.8mg/day, exenatide 10ug/day, lixisenatide 30ug/day).

## Results

### Search results and study characteristics

**Fig 1:**
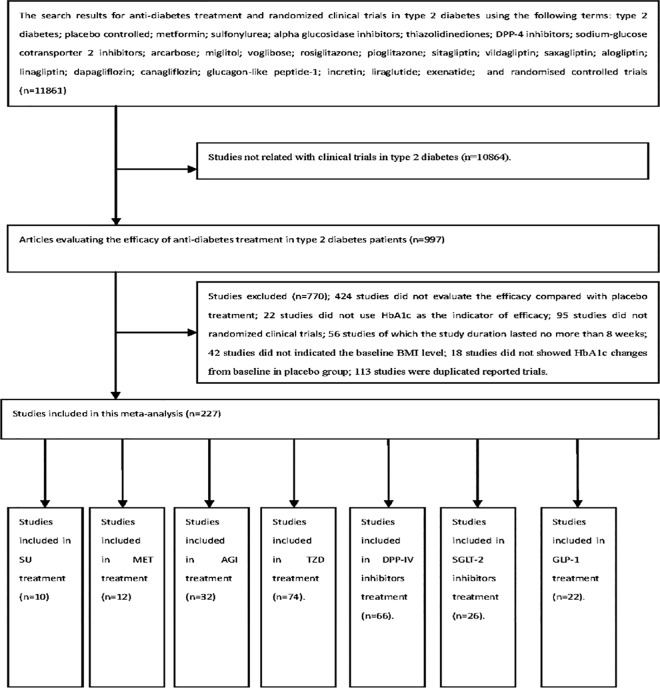
The flowchart of included studies.

The study selection process is summarized in Fig 1. A total of 366 full-text articles were examined in detail, and 227 RCTs were considered to be appropriate for inclusion in the meta-analysis according to our inclusion criteria including 10 studies that compared a sulfonylurea with placebo, which was defined as the sulfonylurea group (SU); 12 trials that compared metformin with placebo (MET); 32 studies that compared an α-glucosidase inhibitor with placebo (AGI); 74 trials that compared a thiazolidinedione with placebo (TZD); 66 trials that compared DPP-IV inhibitors with placebo (DPP-4i); 26 studies that compared SGLT2 inhibitors with placebo (SGLT2i); and 22 studies that compared GLP-1 receptor agonists with placebo (GLP-1). The details are shown in S1 Table.

The analyses were based on data from 1275 individuals in the SU group, 2373 individuals in the MET group, 4059 individuals in the AGI group, 11922 individuals in the TZD group, 20862 individuals in the DPP-4i group, 6737 individuals in the SGLT2i group, and 4768 individuals in the GLP-1 receptor agonists treatment. The baseline characteristics of the patients receiving hypoglycemic agent treatment in the different groups that were stratified by baseline BMI are shown in Table 1.

**Table 1:** Baseline characteristics stratified by baseline BMI in hypoglycemic treatments.

Treatment	Age (years)	Male (%)	BMI(kg/m^2^)	DM duration(years)	HbA1c(%)
**SU**					
**BMI≤25 kg/m^2^**	/	/	/	/	/
**25<BMI<30 kg/m^2^**	55.0±6.7	40.6%	27.4±1.9	12.0±5.1	9.07±1.52
**BMI≥30 kg/m^2^**	56.3±2.4	43.7%	31.7±1.3	7.3±1.0	8.50±0.75
**MET**					
**BMI≤25 kg/m^2^**	/	/	/	/	/
**25<BMI<30 kg/m^2^**	56.0±2.4	40.5%	29.3±0.7	5.0±1.3	7.86±0.67
**BMI≥30 kg/m^2^**	57.5±2.8	43.9%	31.9±2.1	9.1±3.6	9.05±1.21
**AGI**					
**BMI≤25 kg/m^2^**	57.6±0.3	53.6%	24.6±0.3	9.3±1.2	9.70±0.28
**25<BMI<30 kg/m^2^**	61.0±4.0	46.3%	28.0±1.3	6.2±2.9	8.28±1.24
**BMI≥30 kg/m^2^**	57.4±3.0	47.5%	31.7±1.9	6.6±3.1	7.73±1.17
**TZD**					
**BMI≤25 kg/m^2^**	55.9±2.5	46.2%	23.8±1.0	8.1±3.1	8.80±0.81
**25<BMI<30 kg/m^2^**	57.5±3.8	39.1%	28.5±1.3	5.9±2.3	8.06±0.88
**BMI≥30 kg/m^2^**	57.6±3.5	42.5%	31.8±1.8	9.0±3.7	8.50±0.90
**DPP-IV i**					
**BMI≤25 kg/m^2^**	59.5±1.5	34.5%	24.4±0.5	7.2±1.7	8.00±0.40
**25<BMI<30 kg/m^2^**	56.8±5.5	45.0%	28.0±1.7	5.7±2.7	8.26±0.43
**BMI≥30 kg/m^2^**	56.6±3.8	47.6%	31.5±0.9	6.4±3.8	8.19±0.48
**SGLT-2 i**					
**BMI≤25 kg/m^2^**	/	/	/	/	/
**25<BMI<30 kg/m^2^**	56.9±3.6	43.5%	28.5±1.8	5.2±3.5	8.01±0.14
**BMI≥30 kg/m^2^**	56.4±5.9	48.5%	32.2±1.5	7.3±4.8	8.12±0.74
**GLP-1**					
**BMI≤25 kg/m^2^**	58.4±4.1	34.2%	24.0±0.6	9.6±2.8	8.17±0.06
**25<BMI<30 kg/m^2^**	56.1±1.5	38.7%	27.5±2.1	7.2±2.9	8.23±0.31
**BMI≥30 kg/m^2^**	55.2±2.0	44.2%	32.7±3.3	6.3±2.4	8.13±0.40

### Methodological quality

Studies included in this meta-analysis were all placebo controlled, double-blind trials. All the studies reported the inclusion criteria clearly (The details are shown in S1–S7 Figs). Figures of Funnel plots were used to assess the publication bias which suggested an even distribution (data not shown). A high level of study heterogeneity was identified among the studies, suggesting that a random-effects model would accurately describe the data.

### HbA1c changes in hypoglycemic treatment stratified by the baseline BMI

Pooled analysis of the data showed that compared with placebo treatment, treatment with SU resulted in a significant decrease from baseline in HbA_1c_ in overweight patients (WMD, −1.39%; 95% CI, −1.81 to −0.97%, p<0.001) and also a significant decrease in the obese patients (WMD, −0.77%; 95% CI, −1.02 to −0.53%, p<0.001).

Analyses in MET treatment indicated that compared with placebo, the treatment with MET had a significant decrease in HbA_1c_ in overweight patients (WMD, −0.99%; 95% CI, −1.30 to −0.68%, p<0.001), as well as a significant decrease in obese patients (WMD, −1.06%; 95% CI, −1.66 to −0.46%, p<0.001).

Compared with placebo, the AGI treatment was associated with a significant decrease in HbA_1c_ in normal weight patients (WMD, −0.94%; 95% CI, −1.63 to −0.26%, p<0.001), a significant decrease in overweight patients (WMD, −0.72%; 95% CI, −0.80 to −0.64%, p<0.001), and a significant decrease in obese patients (WMD, −0.56%; 95% CI, −0.69 to −0.43%, p<0.001).

Analysis from TZD treatment indicated that treatment with TZD was associated with a significant decrease in HbA_1c_ in normal weight patients (WMD, −1.04%; 95% CI, −1.51 to −0.57%, p<0.001), a significant decrease in overweight patients (WMD, −1.02%; 95% CI, −1.19 to −0.85%, p<0.001), and a significant decrease in obese patients (WMD, −0.88%; 95% CI, −1.01 to −0.75%, p<0.001).

Compared with placebo, treatment with DPP-4i was associated with a significant decrease in HbA_1c_ in normal weight patients (WMD, −0.93%; 95% CI, −1.11 to −0.75%, p<0.001), a significant decrease in overweight patients (WMD, −0.66%; 95% CI, −0.71 to −0.62%, p<0.001), and a significant decrease in obese patients (WMD, −0.61%; 95% CI, −0.67 to −0.54%, p<0.001).

Compared with placebo, treatment with SGLT2i was associated with a significant decrease in HbA_1c_ in overweight patients (WMD, −0.64%; 95% CI, −0.64 to −0.63%, p<0.001) and a significant decrease in obese patients (WMD, −0.60%; 95% CI, −0.70 to −0.51%, p<0.001).

Compared with placebo, treatment with GLP-1 receptor agonists was associated with a significant decrease in HbA_1c_ in normal weight patients (WMD, −1.43%; 95% CI, −2.01 to −0.84%, p<0.001), a significant decrease in overweight patients (WMD, −1.20%; 95% CI, −1.56 to −0.84%, p<0.001), and a significant decrease in obese patients (WMD, −0.96%; 95% CI, −1.04 to −0.88%, p<0.001). The details are shown in Table 2.

**Table 2:** HbA_1c_ changes from baseline stratified by baseline BMI in hypoglycemic treatments.

**Treatment**	**Number of study**	**active hypoglycaemic agents(patients)**	**Placebo (patients)**	**MD (%)**	**95%CI**
**SU**					
**BMI≤25 kg/m^2^**	0	/	/	/	/
**25<BMI<30 kg/m^2^**	4	173	170	-1.39	-1.81 to -0.97
**BMI≥30 kg/m^2^**	6	529	403	-0.77	-1.02 to -0.53
**MET**					
**BMI≤25 kg/m^2^**	0	/	/	/	/
**25<BMI<30 kg/m^2^**	7	594	510	-0.99	-1.30 to -0.68
**BMI≥30 kg/m^2^**	8	641	628	-1.06	-1.66 to -0.46
**AGI**					
**BMI≤25 kg/m^2^**	2	105	104	-0.94	-1.63 to -0.26
**25<BMI<30 kg/m^2^**	15	819	853	-0.72	-0.80 to -0.64
**BMI≥30 kg/m^2^**	17	1170	1008	-0.56	-0.69 to -0.43
**TZD**					
**BMI≤25 kg/m^2^**	6	534	404	-1.04	-1.51 to -0.57
**25<BMI<30 kg/m^2^**	33	2400	2218	-1.02	-1.19 to -0.85
**BMI≥30 kg/m^2^**	37	3494	2872	-0.88	-1.01 to -0.75
**DPP-IV I**					
**BMI≤25 kg/m^2^**	8	805	709	-0.93	-1.11 to -0.75
**25<BMI<30 kg/m^2^**	27	6026	4287	-0.66	-0.71 to -0.62
**BMI≥30 kg/m^2^**	31	4995	4040	-0.61	-0.67 to -0.54
**SGLT-2 i**					
**BMI≤25 kg/m^2^**	0	/	/	/	/
**25<BMI<30 kg/m^2^**	9	1299	1296	-0.64	-0.64 to -0.63
**BMI≥30 kg/m^2^**	17	2160	1982	-0.60	-0.70 to -0.51
**GLP-1**					
**BMI≤25 kg/m^2^**	2	132	134	-1.43	-2.01 to -0.84
**25<BMI<30 kg/m^2^**	3	312	312	-1.20	-1.56 to -0.84
**BMI≥30 kg/m^2^**	17	2141	1737	-0.96	-1.04 to -0.88

Moreover, another subgroup meta-analysis to evaluate the efficacy between Asian and Caucasian in the seven kinds of anti-diabetes agents was shown in Table 3.

**Table 3:** HbA_1c_ changes from baseline stratified by baseline BMI in hypoglycemic treatments between Asian and Caucasian*.

**Treatment**	**Baseline BMI**	**Number of study**	**active hypoglycaemic agents(patients)**	**Placebo (patients)**	**MD (%)**	**95%CI**
**AGI**						
Asian	24.3±0.3	2	105	104	-0.94	-1.63,-0.26
Caucasian	29.7±2.5	29	1989	1861	-0.65	-0.72,-0.58
**TZD**						
Asian	24.5±1.7	10	715	595	-1.37	-1.42,-1.32
Caucasian	31.0±2.1	65	5713	4899	-0.95	-1.05,-0.86
**DPP-IV I**						
Asian	25.6±1.3	18	3198	2409	-0.67	-0.67,-0.67
Caucasian	30.7±1.3	48	8651	6742	-0.60	-0.60,-0.59
**SGLT-2 i**						
Asian	25.1±1.0	2	208	207	-0.89	-1.06,-0.73
Caucasian	32.0±1.5	24	3445	3264	-0.60	-0.65,-0.56
**GLP-1**						
Asian	25.1±1.0	4	403	406	-0.86	-0.88,-0.85
Caucasian	31.2±6.0	18	2182	1777	-0.98	-1.07,-0.90

*In MET, SU group, no study was carried out in Asian population.

### Meta-regression analysis between baseline BMI and HbA1c changes

**Fig 2:**
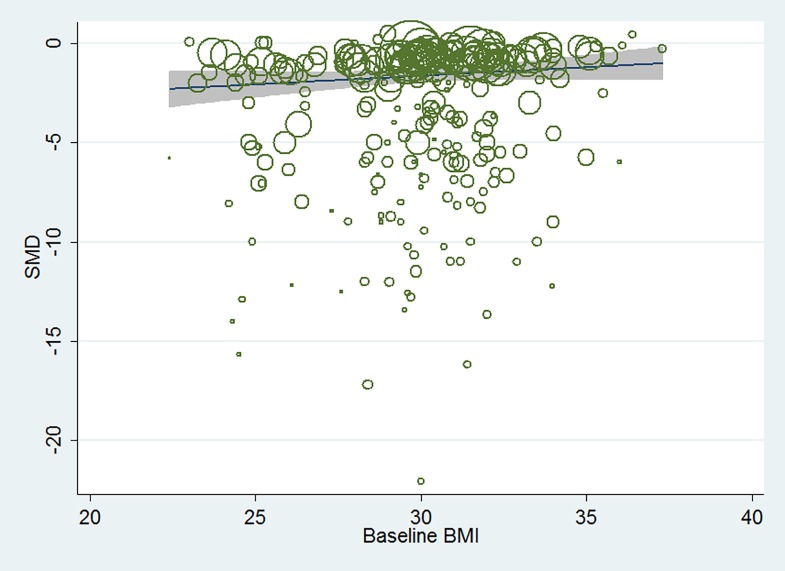
Meta-regression analysis of the association between baseline BMI and the efficacy in HbA1c change in total seven hypoglycemic agents.

**Fig 3:**
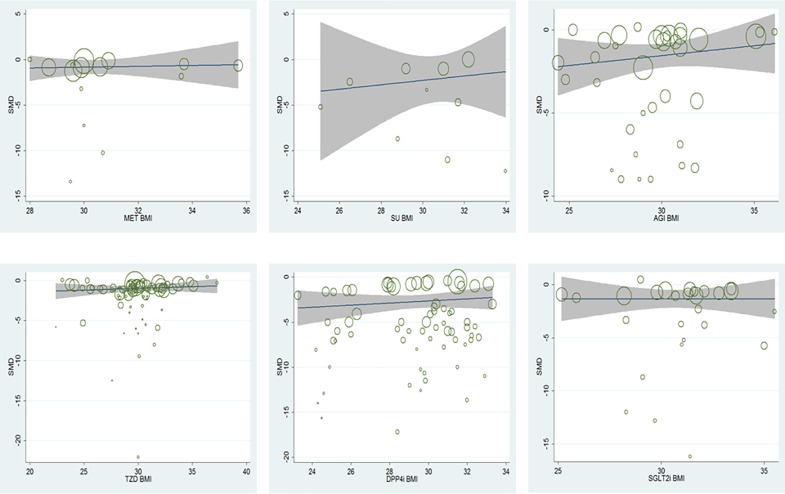
Meta-regression analysis of the association between baseline BMI and the efficacy in HbA1c change in oral hypoglycemic agents.

**Fig 4:**
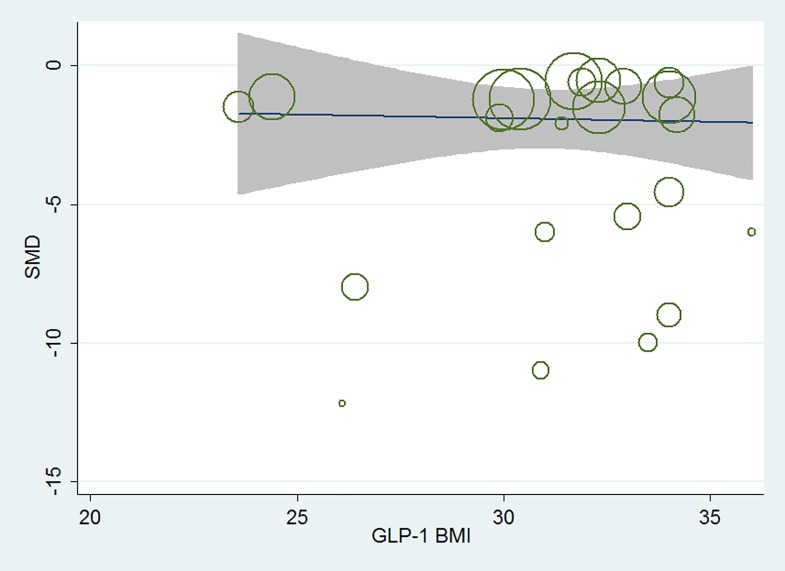
Meta-regression analysis of the association between baseline BMI and the efficacy in HbA1c change in GLP-1 treatment.

In total, of the seven hypoglycemic agents, when compared with placebo, adjusted by the baseline age, gender, duration of diabetes, baseline HbA1c as well as study duration, meta-regression analysis between baseline BMI and HbA1c changes indicated that baseline BMI was not associated with the HbA_1c_ changes from baseline (β, 0.152; 95% CI, -0.023 to 0.328, p = 0.226). Fig 2, Fig 3 and Fig 4 showed the results of meta-regression analysis in the total seven active hypoglycemic agents, the oral agents and GLP-1 receptor agonists separately. In each hypoglycemic treatment, meta-regression analysis also indicated that the baseline BMI was not associated with the HbA_1c_ changes from baseline.

In AGI, adjusted by the baseline age, gender, duration of diabetes, baseline HbA1c as well as study duration, HbA1c changes corrected by placebo was not associated with baseline BMI (β, 0.77; 95% CI, 0.44 to 1.33, p = 0.324); in DPP-4 inhibitors, HbA1c changes corrected by placebo was not associated with baseline BMI (β, 1.09; 95% CI, 0.72 to 1.63, p = 0.679); in GLP-1 receptor agonists, HbA1c changes corrected by placebo was not associated with baseline BMI (β, 1.05; 95% CI, 0.53 to 2.08, p = 0.883); in MET, HbA1c changes corrected by placebo was not associated with baseline BMI (β, 1.38; 95% CI, 0.16 to 11.89, p = 0.713); in SGLT2 inhibitors, HbA1c changes corrected by placebo was not associated with baseline BMI (β, 1.05; 95% CI, 0.18 to 6.30, p = 0.952); in SU, HbA1c changes corrected by placebo was not associated with baseline BMI (β, 0.41; 95% CI, 0.02 to 7.43, p = 0.619); in TZD, HbA1c changes corrected by placebo was not associated with baseline BMI (β, 0.98; 95% CI, 0.62 to 1.55, p = 0.926).

## Discussion

The aim of this meta-analysis is to compare the effects of hypoglycemic treatments in groups of patients categorized according to baseline BMI. The pooled analysis based on a large dataset of individuals, found that in each of the seven types of hypoglycemic treatments, the efficacy in HbA1c changes from baseline was irrespective of baseline BMI, indicating that obese patients can benefit from the same types of hypoglycemic treatments as normal weight patients.

So far, it is still uncertainty regarding whether the efficacy in glucose control with hypoglycemic drugs is different in patients with different BMIs, though it is an important factor and should be taken into account when treating type 2 diabetes patients. According to the results concluded from our meta-analysis, the efficacy in glucose control was not associated with baseline BMI and the possible reasons for explanation might be as follows. In a recently published review [11] talking about the genetic correlation and genetic overlap of obesity and type 2 diabetes, the authors indicated that although type 2 diabetes and obesity are highly interrelated from both epidemiological and pathophysiological viewpoints, the shared identified common variants is limited. Findings from this review might give an explanation to what we have found in our meta-analysis, which indicated that whether patients with obesity or not (represented by the baseline BMI) might not be significantly associated with the efficacy in their T2DM treatments (represented by HbA1c changes). Besides the possible genetic reason, so far, obvious reason is few, but similar results were reported in some trials or meta-analyses of type 2 diabetes patients who were treated with SUs, AGIs, or metformin, DPP-4 inhibitors, GLP-1 receptor agonists.In SU treated patients, a subgroup analysis from the ADVANCE study indicated that [2], the mean HbA1c reduction between the group of baseline BMI <28 and baseline BMI ≥28 suggested no significant difference. In metformin or AGI treated patients, the results from a prospective, multicenter, open-label study in newly diagnosed Chinese type 2 diabetes [7], indicated that baseline BMI had no impact on glycemic control, weight change or other efficacy measures. Another post-hoc analysis [14] of a randomized controlled trial also concluded that both acarbose and metformin similarly decreased the HbA1c levels regardless of the BMI status. In DPP-4 inhibitors treated patients, Schweizer reported a pooled-analysis [15] and the results indicated that it was efficaciously independent of the BMI group. Another meta-analysis [9] reported that the baseline BMI level was not associated with the difference in efficacy between Asian and Caucasian patients. Another meta-analysis [10] indicated that the placebo-subtracted effect of DPP-4 inhibitors on HbA1c was not associated with baseline BMI. In GLP-1 receptor agonists treated patients, a pooled analysis of exenatide treatment [16] and another real-world study of liraglutide treatment [17] also concluded that the treatment has beneficial effects in patients regardless of the baseline BMI.

Contrarily, some conclusions indicated that the baseline BMI was associated with the efficacy in T2DM treatment, which was not associated with that concluded from this meta-analysis. In SU treated patients, a subgroup analyses of the ADOPT study [1], suggested that the treatment effect was significantly greater with rosiglitazone than with glyburide among obese patients (>30 kg/m^2^) compared to overweight patients (≤30 kg/m^2^). In metformin treated patients, a study in Korean T2DM patients [4] indicated that one of the predictors of good response to metformin or rosiglitazone was higher BMI. Moreover, as reported by Jones et al [18], the addition of rosiglitazone to metformin was most effective in obese, insulin-resistant patients with type 2 diabetes. In DPP-4 inhibitors treated patients, two studies in Japanese patients [19] and in Korean T2DM subjects [20] suggested that DPP-4 inhibitor treatment efficacy was associated with a low baseline BMI. In GLP-1 receptor agonists treated patients, a study designed to identify predictors of response to liraglutide therapy in Japanese patients [21] concluded that the efficacy of liraglutide could be associated with BMI at baseline. Another two meta-analyses [8,22] of the efficacy of DPP-4 inhibitor and GLP-1 analogue treatment indicated that different BMI levels might be associated with the efficacy difference between Asian and non-Asian patients.

In consideration of the association between baseline BMI and efficacy in HbA1c changes, another important factor is that baseline HbA1c levels may influence the response to treatment in different baseline BMI groups. Therefore, comparisons of the baseline HbA1c levels among the different BMI groups should be made to determine whether the baseline HbA1c levels are well matched among different BMI categories. In addition, the baseline HbA1c level should be adjusted as a covariator for the association between the baseline BMI level and efficacy. In this meta-analysis, the baseline HbA1c levels were well matched among the different BMI categories, and in meta-regression analysis, the baseline HbA1c level was adjusted as a covariator for the association between the baseline BMI and efficacy, indicating negative results.

This meta-analysis compared the glucose control efficacy of seven types of hypoglycemic treatment with placebo treatment in a large sample of individuals. However, as a meta-analysis, the study has several limitations. Data from separate studies were combined to determine the treatment effects. The inclusion criteria, baseline characteristics, and titrations of the study drugs may be different across studies. Data on baseline mean BMI could only be collected from several studies, and others that lacked baseline mean BMI information were excluded from this analysis, which may indicate the presence of selection bias. What’s more, because the individual participant data was not provided, the mean baseline BMI of each placebo-controlled hypoglycemic treatment was used as a surrogate factor, which made the results of this meta-analysis should be interpreted with cautious. Another possible publication bias is that positive results had a greater chance of being selected for publication than negative results. However, assessment of the funnel plot was performed to minimize this limitation. Therefore, the results should be interpreted cautiously. Additionally, the number of trials included different BMI groups in the hypoglycemic treatment groups was not very well compared, which may be another limitation affecting the results observed in this meta-analysis.

While different types of hypoglycemic treatments have been studied in a variety of clinical studies, the optimal stage for their use in normal weight, overweight and obese patients has not been fully clarified and is still under debate. Based on the known effects on beta-cell function and insulin resistance for type 2 diabetes, there is a perception that hypoglycemic treatment may be less efficacious with increasing insulin resistance in obese patients. However, according to this meta-analysis, each hypoglycemic therapy option that is now widely used in type 2 diabetes patients is efficacious across a wide spectrum of BMIs.

## Conclusion

In each kind of hypoglycemic therapy in type 2 diabetes, the baseline BMI was not associated with the efficacy of HbA1c changes from baseline.

## Supporting Information

S1 Fig The quality of each study and the risk of bias were evaluated by the Cochrane instrument in AGI treatment. (PNG)

S2 Fig The quality of each study and the risk of bias were evaluated by the Cochrane instrument in SU treatment. (PNG)

S3 Fig The quality of each study and the risk of bias were evaluated by the Cochrane instrument in MET treatment. (PNG)

S4 Fig The quality of each study and the risk of bias were evaluated by the Cochrane instrument in TZD treatment. (PNG)

S5 Fig The quality of each study and the risk of bias were evaluated by the Cochrane instrument in DPP-4i treatment. (PNG)

S6 Fig The quality of each study and the risk of bias were evaluated by the Cochrane instrument in SGLT2i treatment. (PNG)

S7 Fig The quality of each study and the risk of bias were evaluated by the Cochrane instrument in GLP-1 treatment. (PNG)

S1 File Supporting Information: PRISMA checklist. (DOCX)

S2 File Supporting Information: Protocol of this meta-analysis. (PDF)

S1 Table Characteristics of randomized controlled trials in type 2 diabetes included in the meta-analysis. (DOCX)
