# Supplementary figures and images for: Baseline Body Mass Index and the Efficacy of Hypoglycemic Treatment in Type 2 Diabetes: A Meta-Analysis
Source: PLoS One. 2016 Dec 9;11(12):e0166625. doi: 10.1371/journal.pone.0166625 (PMC5147850; doi:10.1371/journal.pone.0166625)

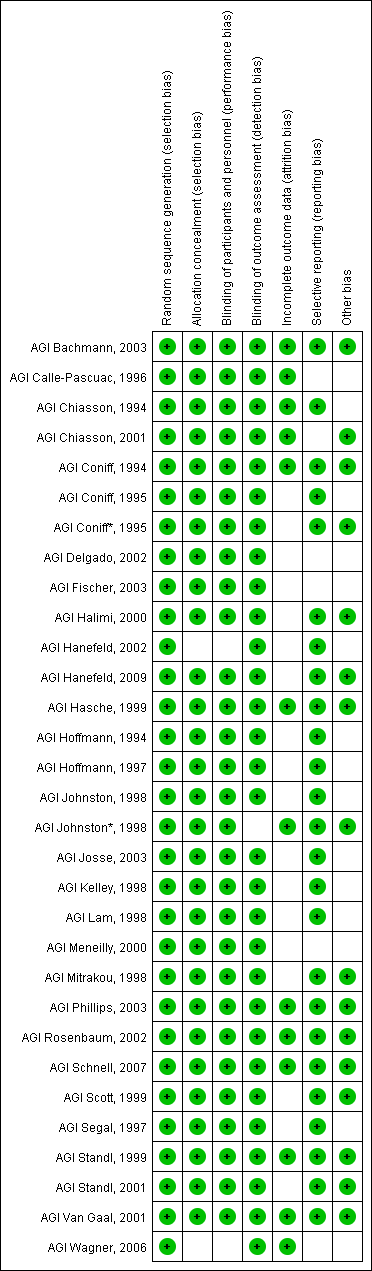

Supplement: S1 Fig — (PNG) [file pone.0166625.s001.png]

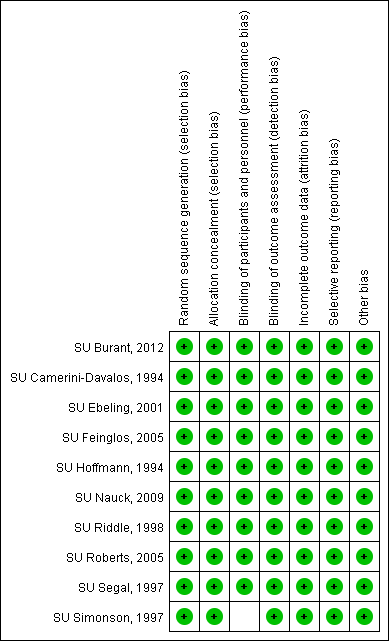

Supplement: S2 Fig — (PNG) [file pone.0166625.s002.png]

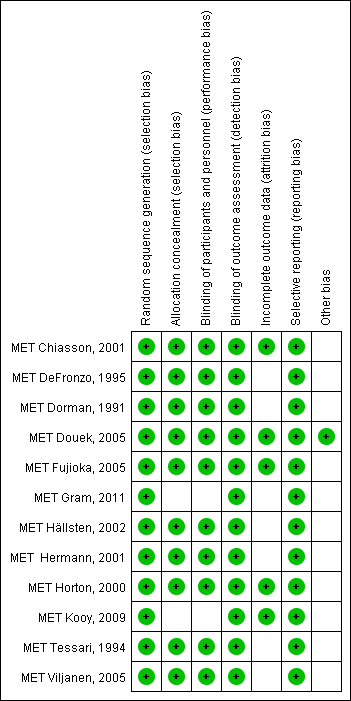

Supplement: S3 Fig — (PNG) [file pone.0166625.s003.png]

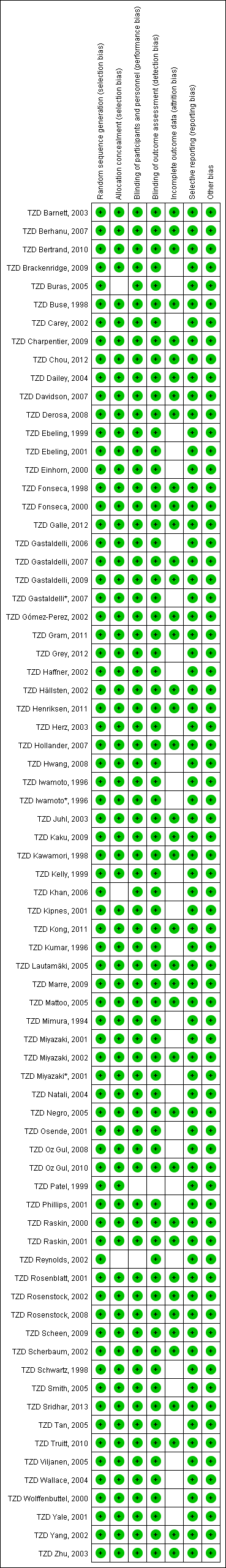

Supplement: S4 Fig — (PNG) [file pone.0166625.s004.png]

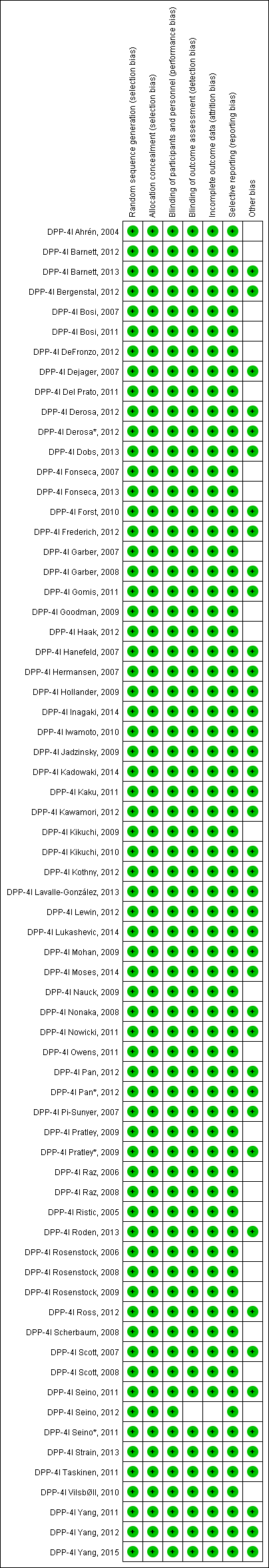

Supplement: S5 Fig — (PNG) [file pone.0166625.s005.png]

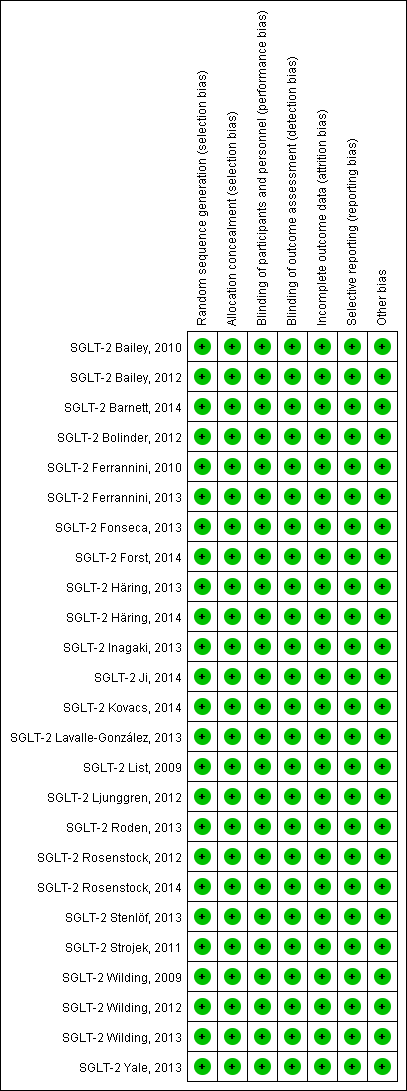

Supplement: S6 Fig — (PNG) [file pone.0166625.s006.png]

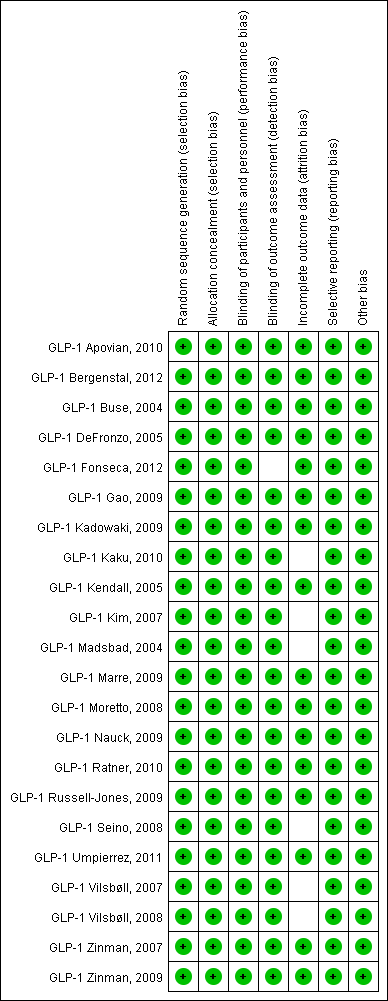

Supplement: S7 Fig — (PNG) [file pone.0166625.s007.png]
