# Supplementary material for: Baseline Body Mass Index and the Efficacy of Hypoglycemic Treatment in Type 2 Diabetes: A Meta-Analysis
Source: PLoS One. 2016 Dec 9;11(12):e0166625. doi: 10.1371/journal.pone.0166625 (PMC5147850; doi:10.1371/journal.pone.0166625)
Supplement: S2 File — (PDF) [file pone.0166625.s009.pdf]

Efficacy of hypoglycemic treatment in type 2 diabetes according to baseline body mass index: a meta-analysis

Protocol

Study Rationale

The efficacy of glucose lowering in different anti-diabetes drugs has been well known, however the effects on HbA1c changes as well as body weight changes in obese people compared with people of normal weight might depend on choice of drug.

There is an uncertainty that treatment with anti-diabetes drugs might be different in diverse patients. Investigators did a series of post-hoc analyses comparing the effects of glucose lowering drugs at different levels of baseline body-mass index (BMI) in the clinical trials with inconsistent findings. Some meta-analysis (Kim et al) also indicated that baseline BMI might be associated with the different efficacy on glucose changes in some hypoglycemic treatment. However, the association between baseline BMI and treatment efficacy was underpinned in that there was not a comprehensive manuscript focused on this association so far.

Therefore, the aim of this meta-analysis is to compare the effects of blood glucose lowering regimens in groups of patients categorized by baseline BMI.

Hypothesis

Hypothesis is that the effects on blood glucose control of blood glucose lowering regimens might be different in groups of patients categorized by baseline BMI. The aim of this meta-analysis is to compare the effects of blood glucose lowering regimens in groups of patients categorized by baseline BMI.

Primary objectives

- To compare the efficacy of glucose lowering in different anti-diabetes treatment stratified by baseline BMI.

Method:

Data collection: Meta-analysis and studies are identified by a literature search of MEDLINE® (PubMed), EMBASE® and the Cochrane Central Register of Controlled Trials (CENTRAL) from when recording began until December 2014. The electronic search was first conducted in January 2015 and repeated in June 2015. Moreover, documents for approved medications could be searched for trials at the clinicaltrials website (http://www.clinicalstudyresults.org and http://www.clinicaltrials.gov).

Searching Strategy: type 2 diabetes; metformin; sulfonylurea; alpha glucosidase inhibitors; thiazolidinediones; DPP-4 inhibitors; sodium-glucose cotransporter 2 inhibitors; arcarbose; miglitol; voglibose; rosiglitazone; pioglitazone; sitagliptin; vildagliptin; saxagliptin; alogliptin; linagliptin; dapagliflozin; canagliflozin; empagliflozin and randomised controlled trials.

Participants: RCTs in T2DM patients to compare the efficacy of OADs with placebo or active anti-diabetes agents and the treatment duration is more than 12 weeks

Inclusion criteria

- Placebo-controlled or active-controlled randomized anti-diabetic treatment trial carried out in type 2 diabetes participants
- Study length of ≥12 weeks
- Change in glucose was assessed as change in HbA1c from baseline during the clinical trial in the comparative groups
- Baseline BMI was reported in the trial

Exclusion criteria

- Active-controlled non-randomized anti-diabetic treatment trial carried out in type 2 diabetes participants
- Trials in type 1 diabetes
- Study length less than 12 weeks

- Baseline BMI was not reported in the trial
- Change in glucose was not assessed as change in HbA1c from baseline

Study selection

- The eligibility of all studies retrieved from the databases should be screened independently by two review authors in duplicate based on predetermined inclusion criteria. Disagreements between reviewers should be resolved by consultation with a third investigator. The quality of each study should be evaluated by the researchers according to standard scores.

Data extraction

- Two review authors independently will extract the following data from each publication using a standardised form: publication data (title, first author, year and source of publication, study design, baseline characteristics of the study population (sample size, age, duration of T2DM, HbA_{1c}), description of study drugs, treatment duration, primary outcome measures (change from baseline to study endpoint for HbA_{1c}). Disagreements or discrepancies should be resolved by discussion between the two review authors and also discussed with a third investigator (ZLL).

Main Assessments: HbA1c change from baseline, Goal attainment rate (HbA1c<7.0%) stratified by the baseline BMI (<25kg/m^2, 25-29.9 kg/m^2, ≥30 kg/m^2).

Analysis methodology

BMI was calculated as the formula of weight (kg) divided by height (m^2). Studies according to baseline BMI were divided into three groups: normal weight (BMI<25 kg/m^2), overweight (25 kg/m^2≤BMI<30 kg/m^2), obese (BMI≥30 kg/m^2). Meta-analysis was performed in each group stratified by baseline BMI for the evaluation of the HbA1c changes from baseline in different anti-diabetes treatment.

The meta-analysis was performed by computing the weighted mean difference (WMD) and 95%

confidence interval (CIs) for change from baseline to study endpoint for placebo treatment in different anti-diabetic therapies. Between-study heterogeneity was examined using Q^2 statistics, which was then distributed as χ^2 statistics; statistical significance was indicated by $p < 0.10$. Higgins I^2 statistics were used to quantify the percentage of total variance in the summary estimate due to between-study heterogeneity. An I^2 value >50% represented substantial variability. The extent of study heterogeneity determined whether a fixed effects or random-effects model was used in the analysis. Fixed-effects and random-effects models were used with low and high levels of heterogeneity, respectively. Publication bias was assessed via visual inspection of the funnel plot. All statistical analyses were performed with the Review Manager statistical software package (Version 5.1). This meta-analysis was conducted according to the PRISMA guidelines for the conduct and reporting of meta-analyses of RCTs. Regression was performed for the association analysis of the baseline characteristics and the efficacy of anti-diabetes treatment. Meta-regression analyses were performed with the STATA statistical software package (Version 11.0). Results were expressed as p-values for the interaction term (where $p < 0.05$ represented a significant interaction).Descriptive analysis will be used for the incidence of adverse events and the characteristics of demography and baseline for each group.
