# Supplementary material for: Baseline Body Mass Index and the Efficacy of Hypoglycemic Treatment in Type 2 Diabetes: A Meta-Analysis
Source: PLoS One. 2016 Dec 9;11(12):e0166625. doi: 10.1371/journal.pone.0166625 (PMC5147850; doi:10.1371/journal.pone.0166625)
Supplement: S1 Table — (DOCX) [file pone.0166625.s010.docx]

**Supplement table S1:**

| **Author , year** | **Study duration** | | **Treatment group** | **No. of patients** | **Age (years)** | **Men (%)** | **BMI (kg/m^2^)** | **DM duration**  **(years)** | **HbA1c change (%)** | **Baseline HbA1c (%)** | **Baseline Weight**  **(kg)** | | **Weight Change**  **(kg)** | |
| --- | --- | --- | --- | --- | --- | --- | --- | --- | --- | --- | --- | --- | --- | --- |
| **SU versus Placebo, monotherapy** | | | | | | | | | | |  | |  | |
| Ebeling, 2001[^1^](#_ENREF_1) | | 6 months | Glibenclamide 2.5mg qd | 10 | / | / | 30.2±1.7 | / | -1.2±0.3 | 8.9±0.3 | / | | / | |
|  |  |  | Pioglitazone 30mg qd | 9 | / | / | 30.5±1.3 | / | -1.1±0.5 | 9.1±0.3 | / | | / | |
|  |  |  | PBO | 10 | / | / | 31.9±1.5 | / | -0.2±0.3 | 8.6±0.2 | / | | / | |
| Hoffmann, 1994[^2^](#_ENREF_2) | | 24 weeks | Glibenclamide 4.3mg qd | 27 | 59.5±5.7 | 48 | 26.5±2.1 | 1.4±1.1 | -0.8±0.37 | 8.30±0.37 | / | | / | |
|  |  |  | Acarbose 100mg tid | 28 | 58.8±6.9 | 46 | 26.5±1.6 | 1.0±0.9 | -0.99±0.42 | 8.29±0.42 | / | | / | |
|  |  |  | PBO | 30 | 56.9±6.7 | 40 | 26.8±1.5 | 1.0±0.9 | 0.11±0.37 | 8.29±0.37 | / | | / | |
| Segal, 1997[^3^](#_ENREF_3) | | 24 weeks | Miglitol 100mg tid | 40 | 61 | 55 | 28.6 | / | -0.60±1 | 7.95 | / | | -1.4±1 | |
|  |  |  | Glibenclamide 3.5mg bid | 37 | 56 | 62.2 | 29.2 | / | -0.86±1 | 7.96 | / | | +1.4±1 | |
|  |  |  | PBO | 42 | 59 | 57.1 | 29.1 | / | 0.15±1 | 8.25 | / | | 0±1 | |
| Simonson, 1997[^4^](#_ENREF_4) | | 16 weeks | glipizide 5mg | 68 | 57.4 | 58.8 | 29 | 6.9 | -0.9±0.2 | 8.5±0.2 | 83.9 | | -0.14±0.18 | |
|  |  |  | glipizide 10mg | 42 | 58.7 | 59.5 | 28.4 | 8.8 | -1.2±0.2 | 8.8±0.2 | 82.1 | | / | |
|  |  |  | glipizide 15mg | 42 | 55.5 | 61.9 | 29.5 | 6.5 | -0.49±0.2 | 8.6±0.2 | 84.8 | | / | |
|  |  |  | glipizide 20mg | 69 | 59.3 | 68.1 | 28.8 | 7.8 | -0.9±0.2 | 8.7±0.2 | 84.8 | | / | |
|  |  |  | glipizide 40mg | 28 | 61.7 | 60.7 | 29.6 | 6.6 | -1.27±0.3 | 8.4±0.3 | 88.9 | | / | |
|  |  |  | glipizide 60mg | 29 | 56.9 | 79.3 | 30.5 | 5.3 | -1.1±0.3 | 8.6±0.3 | 91.2 | | / | |
|  |  |  | PBO | 69 | 60.2 | 76.8 | 29.7 | 7.5 | 0.84±0.2 | 8.3±0.2 | 87.1 | | -3.1±0.4 | |
| **SU versus Placebo, add-on therapy** | | | | | | | | | | |  | |  | |
| Burant, 2012[^5^](#_ENREF_5) | | 12 weeks | TAK-875 6.25mg Qd | 60 | 51.6±10.4 | 48 | 32.2±5.3 | 5.7±5.2 | -0.65±1.0 | 8.60±0.96 | 85.8±18.4 | | / | |
|  |  |  | TAK-875 25mg Qd | 61 | 51.3±10.9 | 46 | 32.3±4.6 | 5.2±4.6 | / | 8.23±0.93 | 88.4±22.7 | | / | |
|  |  |  | TAK-875 50mg Qd | 60 | 52.1±10.8 | 47 | 31.0±4.6 | 6.2±5.4 | -1.12±0.8 | 8.33±0.80 | 83.7±16.3 | | / | |
|  |  |  | TAK-875 100mg Qd | 62 | 51.8±10.1 | 47 | 32.3±5.4 | 4.8±3.8 | -1.0±0.9 | 8.33±0.87 | 88.9±22.5 | | / | |
|  |  |  | TAK-875 200mg Qd | 60 | 49.0±10.7 | 48 | 31.3±4.8 | 6.4±5.0 | / | 8.56±0.93 | 84.6±18.3 | | / | |
|  |  |  | glimepiride 4mg Qd | 62 | 52.2±9.7 | 55 | 31.0±5.4 | 6.4±5.7 | -1.05±0.8 | 8.43±0.81 | 83.5±17.8 | | +0.86±17.8 | |
|  |  |  | PBO | 61 | 52.9±11.3 | 43 | 31.2±5.0 | 5.6±4.8 | -0.13±1.0 | 8.46±1.07 | 84.5±20.7 | | -0.73±20.7 | |
| Camerini-Davalos, 1994[^6^](#_ENREF_6) | | 3 years | Glipizide 5mg qd+insulin | 40 | 45.2±1.7 | / | 25.1±0.2 | 10.5 | -1.6±0.3 | 11.2±0.3 | / | | / | |
|  |  |  | PBO+insulin | 29 | 46.4±2.0 | / | 24.3±0.4 | 14.1 | 0.2±0.4 | 10.3±0.3 | / | | / | |
| Feinglos, 2005[^7^](#_ENREF_7) | | 16 weeks | glipizide 2.5mg/day+met | 61 | 57.7±10.7 | 45.9 | 31.7±4.4 | 6.5 | -0.66±0.1 | 7.45 | 90±18.7 | | +0.4±18.7 | |
|  |  |  | PBO+met | 61 | 58.8±10.0 | 41 | 32.1±4.9 | 4.6 | -0.19±0.1 | 7.64 | 90.8±18.4 | | -1.7±18.4 | |
| Nauck, 2009[^8^](#_ENREF_8) | | 26 weeks | liraglutide 0.6mg/day+met | 242 | 56±11 | 62 | 30.5±4.8 | 7±5 | -0.7±0.1 | 8.4±0.9 | / | | -1.8±0.2 | |
|  |  |  | liraglutide 1.2mg/day+met | 240 | 57±9 | 54 | 31.1±4.8 | 7±5 | -1.0±0.1 | 8.3±1.0 | / | | -2.6±0.2 | |
|  |  |  | liraglutide 1.8mg/day+met | 242 | 57±9 | 59 | 30.9±4.6 | 8±5 | -1.0±0.1 | 8.4±1.0 | / | | -2.8±0.2 | |
|  |  |  | glimepiride 4mg/day+met | 242 | 57±9 | 57 | 31.2±4.6 | 8±5 | -1±0.1 | 8.4±1.0 | / | | +1.0±0.2 | |
|  |  |  | PBO+met | 121 | 56±9 | 60 | 31.6±4.4 | 8±6 | 0.1±0.1 | 8.4±1.1 | / | | -1.5±0.3 | |
| Riddle, 1998[^9^](#_ENREF_9) | | 24 weeks | Glimepiride 8mg bid+insulin | 72 | 58±8 | 62.5 | 32.2±4.4 | 7±4 | -2.1±1.2 | 9.7±1.3 | 93.9±15.9 | | +4.3±16.5 | |
|  |  |  | PBO+insulin | 73 | 58±8 | 54.8 | 33.7±5.4 | 7±4 | -2.1±1.2 | 9.8±1.3 | 99.2±20.8 | | +4.0±20.8 | |
| Roberts, 2005[^10^](#_ENREF_10) | | 26 weeks | Glimepiride 8mg qd+metformin+SU | 82 | 56.5±9.8 | 61 | 33.98±5.15 | 8.7±6.8 | -1.31±0.08 | 8.15±0.76 | 100.88±18.97 | | +3.76±0.54 | |
|  |  |  | PBO+metformin+SU | 77 | 56.4±10.0 | 62.3 | 32.76±5.11 | 7.9±4.9 | -0.33±0.08 | 8.15±0.65 | 96.31±19.1 | | +0.45±0.52 | |
| **Author , year** | | **Study duration** | **Treatment group** | **No. of patients** | **Age (years)** | **Men (%)** | **BMI (kg/m2)** | **DM duration**  **(years)** | **HbA1c change (%)** | **Baseline HbA1c (%)** | **Baseline Weight**  **(kg)** | | **Weight Change**  **(kg)** | |
| **MET versus Placebo, monotherapy** | | | | | | | | | | |  | |  | |
| Chiasson, 2001[^11^](#_ENREF_11) | | 36 weeks | Miglitol 100mg tid | 82 | 57.3±9.0 | 78.1 | 31.1±4.5 | 5.2±4.7 | 0.02±0.10 | 8.2±0.9 | 91±15.5 | | -0.42±0.29 | |
|  | |  | Metformin 500mg tid | 83 | 57.9±8.6 | 73.5 | 30.7±5.1 | 7.5±7.4 | -0.85±0.12 | 8.2±0.9 | 89±17.8 | | -0.79±0.33 | |
|  |  |  | miglitol+metformin | 76 | 58.9±7.9 | 77.6 | 29.5±3.8 | 6.1±5.5 | -1.39±0.11 | 8.3±0.8 | / | | / | |
|  | |  | placebo | 83 | 57.7±9.9 | 67.5 | 31.1±4.4 | 5.1±4.9 | 0.38±0.12 | 8.1±0.7 | 88.6±14.1 | | -0.69±0.27 | |
| DeFronzo, 1995[^12^](#_ENREF_12) | | 29weeks | Metformin 2550mg qd | 143 | 53±1 | 43.4 | 29.9±0.3 | 6.0±0.5 | -1.4±0.1 | 8.4±0.1 | 94.4±1.1 | | -0.6±0.3 | |
|  |  |  | placebo | 146 | 53±1 | 42.5 | 29.2±0.3 | 6.0±0.6 | 0.4±0.1 | 8.2±0.2 | 92.2±1.2 | | -1.1±1.2 | |
| Dornan, 1991[^13^](#_ENREF_13) | | 8 months | Metformin 1g tid | 30 | 55±1 | / | 30±1 | / | -1.4±0.4 | 11.7±0.4 | 84.6±2.7 | | 0±2.7 | |
|  |  |  | placebo | 30 | 55±1 | / | 30±1 | / | 1.5±0.4 | 11.8±0.4 | 79.5±2.5 | | -0.9±2.5 | |
| Fujioka, 2005[^14^](#_ENREF_14) | | 24 weeks | metformin 1000mg once daily | 161 | 55±11 | 53 | 28.7±3.9 | 3.3±2.8 | -0.6±0.9 | 8.1±0.9 | / | | / | |
|  |  |  | placebo | 79 | 58±11 | 63 | 28.9±3.5 | 3.2±2.6 | 0.2±0.9 | 7.9±0.9 | / | | / | |
|  |  | 16 weeks | metformin 500mg once daily | 128 | 55±11 | 44 | 30.1±4.0 | 3.3±2.9 | -0.5±0.9 | 8.2±0.9 | / | | / | |
|  |  |  | metformin 1000mg once daily | 120 | 56±10 | 58 | 30.6±4.2 | 3.0±2.7 | -0.6±1.1 | 8.4±1.1 | / | | / | |
|  |  |  | metformin 1500mg once daily | 120 | 56±11 | 48 | 29.7±4.0 | 2.9±2.7 | -0.9±2.6 | 9.9±2.6 | / | | / | |
|  |  |  | metformin 2000mg once daily | 134 | 55±11 | 41 | 30.9±4.0 | 2.7±2.5 | -0.9±1.1 | 8.4±1.1 | / | | / | |
|  |  |  | metformin 1000mg twice daily | 123 | 57±10 | 47 | 30.6±4.6 | 3.1±2.7 | -1.1±1.1 | 8.4±1.1 | / | | / | |
|  |  |  | placebo | 117 | 54±10 | 49 | 30.7±4.1 | 2.7±2.7 | 0.1±1.1 | 8.3±1.1 | / | | / | |
| Hällsten, 2002[^15^](#_ENREF_15) | | 26 weeks | metformin 1g bid | 13 | 57.8±2.2 | 61.5 | 29.9±1.1 | / | -0.7±0.2 | 6.9±0.2 | / | | / | |
|  |  |  | rosiglitazone 4mg bid | 14 | 58.6±2.0 | 71.4 | 29.3±1.0 | / | -0.3±0.2 | 6.8±0.2 | / | | / | |
|  |  |  | placebo | 14 | 57.7±1.9 | 71.4 | 30.3±1.2 | / | -0.2±0.1 | 6.3±0.1 | / | | / | |
| Horton, 2000[^16^](#_ENREF_16) | | 24 weeks | metformin 500mg tid | 178 | 56.8±10.9 | 68.5 | 29.6±4.3 | 4.5±5.5 | -0.8±1.1 | 8.4±1.2 | / | | / | |
|  |  |  | nateglinide 120mg tid | 179 | 58.6±10.7 | 61.5 | 29.6±3.8 | 4.7±5.5 | -0.5±1.0 | 8.3±1.0 | / | | / | |
|  |  |  | nateglinide+metformin | 172 | 58.4±10.9 | 58.7 | 30.0±3.7 | 4.5±5.3 | -1.4±1 | 8.4±1.1 | / | | / | |
|  |  |  | placebo | 172 | 59.6±10.9 | 60.5 | 29.2±3.9 | 4.6±4.7 | 0.5±1.1 | 8.3±1.1 | / | | / | |
| Tessari, 1994[^17^](#_ENREF_17) | | 4 weeks | Metformin 850mg bid | 11 | 53±3 | 54.5 | 28±1 | / | -0.1±0.8 | 8.0±0.6 | / | | / | |
|  |  |  | placebo | 6 | 60±3 | 33.3 | 28±1 | / | -0.1±0.5 | 6.7±0.3 | / | | / | |
| Viljanen, 2005[^18^](#_ENREF_18) | | 26 weeks | Rosiglitazone 4mg bid | 14 | 58.6±7.7 | 64.3 | 29.3±4.0 | / | -0.3±0.9 | 6.8±0.9 | 83.7±8.1 | | +0.6±9.2 | |
|  |  |  | Metformin 1g bid | 12 | 57.8±8.7 | 58.3 | 29.6±4.0 | / | -0.6±0.9 | 6.9±0.9 | 87.6±10.7 | | -1.8±10.7 | |
|  |  |  | PBO | 11 | 58.7±8.3 | 81.8 | 29.8±4.1 | / | -0.1±0.7 | 6.2±0.7 | 89.1±9.3 | | +0.4±9.4 | |
| **MET versus Placebo, add-on therapy** | | | | | | | | | | |  | |  | |
| Douek, 2005[^19^](#_ENREF_19) | | 12 months | Metformin 2g qd+insulin | 92 | 58±8.9 | 67.4 | 30.9±4.5 | 9±5.2 | -1.5±1.4 | 9.7±1.3 | 88.5±14.7 | | 6.3±14.7 | |
|  |  |  | Placebo+insulin | 91 | 58±7.7 | 62.6 | 31.5±4.3 | 10±5.2 | -1.3±1.5 | 10.0±1.5 | 91.1±15.7 | | 7.5±15.7 | |
| Gram, 2011[^20^](#_ENREF_20) | | 2 years | PBO+NPH | 46 | 55.8±7.7 | 71.7 | 34.0±6.0 | 7.3±4.3 | -0.4±1.4 | 8.7±1.3 | 100.2±19.8 | | +5.3±20 | |
|  |  |  | Metformin 1000mg bid+NPH | 45 | 55.4±8.5 | 57.8 | 35.7±6.4 | 8.2±4.0 | -1.3±1.3 | 8.9±1.2 | 105.1±17.7 | | 2.9±20 | |
|  |  |  | Rosiglitazone 4mg bid+NPH | 46 | 57.3±8.9 | 60.9 | 34.0±5.7 | 9.2±6.9 | -0.8±1.6 | 8.7±1.2 | 100.9±16.5 | | 7.6±20 | |
|  |  |  | metformin+rosiglitazone  +NPH | 46 | 57.3±8.2 | 65.2 | 34.4±7.0 | 8.1±5.3 | -1.7±1.1 | 8.5±1.1 | / | | / | |
|  |  |  | PBO+ASP | 48 | 57.1±8.5 | 47.9 | 33.7±5.0 | 9.1±5.5 | -0.6±1.2 | 8.5±1.2 | 98.3±16.6 | | 6.1±19 | |
|  |  |  | Metformin 1000mg bid+ASP | 45 | 56.1±8.2 | 62.2 | 33.7±6.1 | 8.7±4.5 | -1.2±1.2 | 8.5±1.2 | 100.5±17.9 | | 3.9±20 | |
|  |  |  | Rosiglitazone 4mg bid+ASP | 47 | 56.1±8.3 | 57.4 | 32.7±4.7 | 9.4±6.3 | -1.2±1 | 8.3±1.0 | 95.6±14.6 | | 10.4±20 | |
|  |  |  | metformin+rosiglitazone  +ASP | 48 | 55.3±9.1 | 70.8 | 32.9±4.4 | 9.0±5.8 | -1.5±1.2 | 8.5±1.2 | / | | / | |
| Hermann, 2001[^21^](#_ENREF_21) | | 12 months | metformin 850mg bid+insulin | 16 | 56.9±10.2 | 43.8 | 33.6±3.5 | 13 | -1.1±1.3 | 9.1±1.3 | 96.4±16.5 | | -1.4±16.5 | |
|  |  |  | Placebo+insulin | 19 | 58.1±9.7 | 63.2 | 32.6±3.8 | 13 | 0.3±1.0 | 8.7±1.0 | 94.2±9.4 | | +0.2±9.4 | |
| Kooy, 2009[^22^](#_ENREF_22) | | 4.3 years | metformin 850mg tid hydrochloride+insulin | 196 | 64±10 | 41.3 | 30±5 | 14±9 | -0.2±1.1 | 7.9±1.2 | 85±16 | | +2±17 | |
|  |  |  | placebo+insulin | 194 | 59±11 | 50 | 30±5 | 12±8 | 0.0±1.1 | 7.9±1.2 | 87±15 | | +4±17 | |
| **Author , year** | | **Study duration** | **Treatment group** | **No. of patients** | **Age (years)** | **Men (%)** | **BMI (kg/m2)** | **DM duration**  **(years)** | **HbA1c change (%)** | **Baseline HbA1c (%)** | **Baseline Weight**  **(kg)** | | **Weight Change**  **(kg)** | |
| **AGI versus Placebo, monotherapy** | | | | | | | | | | |  | |  | |
| Calle-Pascuac, 1996[^23^](#_ENREF_23) | | 16 weeks | Acarbose 100mg tid | 20 | / | / | 35.3±8.8 | / | -0.2±0.93 | 6.3±0.8 | 88.7±18.2 | | -5.4±18.2 | |
|  |  |  | Placebo | 20 | / | / | 35.9±7.6 | / | 0±1.48 | 6.4±1.3 | 89.5±16.1 | | -1.3±16.1 | |
| Chiasson, 1994[^24^](#_ENREF_24) | | 1 year | Acarbose 200mg qd | 38 | / | / | 28.8±0.5 | / | -0.9±0.2 | 6.7±0.2 | 84.5±1.2 | | -0.3±1.6 | |
|  |  |  | Placebo | 39 | / | / | 28.8±0.5 | / | 0±0.2 |  | 81.3±1.2 | | +0.1±1.3 | |
| Chiasson, 2001[^11^](#_ENREF_11) | | 36 weeks | Placebo | 83 | 57.7±9.9 | 67.5 | 31.1±4.5 | 5.1±4.9 | -0.235±0.06 | 8.1±0.7 | 91±15.5 | | -0.42±0.29 | |
|  |  |  | Miglitol | 82 | 57.3±9 | 78 | 31.1±4.4 | 5.2±4.7 | -0.685±0.05 | 8.2±0.9 | 88.6±14.1 | | -0.69±0.27 | |
| Coniff, 1994[^25^](#_ENREF_25) | | 36 weeks | Acarbose 50-300mg tid | 91 | 56.0±1.0 | 54 | 32 | 4 | -0.06±1 | 6.78 | 93.4±1.8 | | -0.93±1.8 | |
|  |  |  | Placebo | 98 | 55.6±1.0 | 45 | 31.5 | 3 | 0.53±1.0 | 6.65 | 94.5±1.7 | | -0.77±1.7 | |
| Coniff, 1995[^26^](#_ENREF_26) | | 16 weeks | Acarbose 100mg tid | 58 | 55 | 53 | 31 | 6 | -0.45±1 | 8.69 | 85.9 | | -0.19±1 | |
|  |  |  | Acarbose 200mg tid | 54 | 56 | 59 | 31 | 5 | -0.4±1 | 8.96 | 87.9 | | -0.8±1 | |
|  |  |  | Acarbose 300mg tid | 53 | 54 | 58 | 30 | 5 | -0.77±1 | 9.54 | 84.5 | | -0.45±1 | |
|  |  |  | Placebo | 64 | 54 | 58 | 32 | 5 | 0.33±1.0 | 8.67 | 91.3 | | -0.37±1 | |
| Coniff, 1995[^27^](#_ENREF_27) | | 36 weeks | Acarbose 200mg tid | 67 | 56.2 | 39 | 29.7 | 5.1 | -0.54±1 | 6.88 | 81.6 | | -1.4±1 | |
|  |  |  | Tolbutamide 250-1000mg tid | 66 | 55.4 | 56 | 29.5 | 5.6 | -0.93±1.0 | 6.95 | 84.8 | | +1.8±1 | |
|  |  |  | Placebo | 62 | 56.3 | 52 | 29.9 | 5.5 | 0.04±1.0 | 7.1 | 85.8 | | -1.4±1 | |
| Delgado, 2002[^28^](#_ENREF_28) | | 4 months | Acarbose 100mg qd | 9 | / | / | 36.1±1.5 | / | -0.1±0.5 | 6.8±0.5 | 103.8±2.9 | | 0.8±2.9 | |
|  |  |  | Placebo | 8 | / | / | 34.4±2.8 | / | 0±1 | 7.5±0.6 | 90.6±8.9 | | 0.5±8.9 | |
| Fischer, 2003[^29^](#_ENREF_29) | | 16 weeks | Acarbose 100mg tid | 25 | 59.4±5.6 | / | 27.3±0.8 | 7.8±0.9 | 0±0.11 | 8.1±0.2 | 78.2±2.3 | | -1.5±2.4 | |
|  |  |  | Placebo | 25 | 58.6±6.3 | / | 27.0±0.7 | 6.4±0.9 | 0.7±0.04 | 8.3±0.3 | 79.2±1.8 | | -1.3±1.8 | |
| Hanefeld, 2002[^30^](#_ENREF_30) | | 16 weeks | Acarbose 100mg tid | 11 | 60.4±1.3 | 91 | 27.5±0.7 | 7.7±1.3 | 0.4±0.6 | 8.2±0.3 | / | | / | |
|  |  |  | Glibenclamide 1mg tid | 8 | 60.6±2.5 | 62.5 | 27.1±1.1 | 7.1±1.2 | -0.8±0.6 | 8.4±0.4 | / | | / | |
|  |  |  | Placebo | 8 | 59±1.6 | 75 | 27.2±1.1 | 6.8±1.6 | 0.9±0.6 | 8.7±0.6 | / | | / | |
| Hanefeld, 2009[^31^](#_ENREF_31) | | 20 weeks | Acarbose 100mg tid | 42 | 62.33±8.7 | / | 31.02±5.12 | / | -0.07±0.55 | 6.11±0.48 | / | | / | |
|  |  |  | Placebo | 45 | 59.92±10.0 | / | 30.78±3.70 | / | -0.03±0.75 | 6.09±0.66 | / | | / | |
| Hasche, 1999[^32^](#_ENREF_32) | | 24 months | Acarbose 100mg tid | 52 | / | / | 26.9±3.1 | / | -1.71±1.6 | 8.9±0.8 | 74.9±8.7 | | -1.4±2.8 | |
|  |  |  | Placebo | 48 | / | / | 26.2±2.4 | / | -0.82±1.1 | 8.7±0.9 | 74.5±8.6 | | -1.3±3 | |
| Hoffmann, 1997[^33^](#_ENREF_33) | | 24 weeks | Acarbose 100mg tid | 31 | 58.9±9.4 | 19 | 26.4±2.7 | 3.0±2.3 | -1.1±0.9 | 9.6±0.9 | 73.9±10.3 | | -0.8±10.3 | |
|  |  |  | Metformin 850mg bid | 31 | 55.9±7.8 | 45 | 27.4±2.2 | 2.1±1.5 | -1.0±0.9 | 9.7±0.9 | 79±8.8 | | +0.2±8.8 | |
|  |  |  | Placebo | 32 | 60.2±8.6 | 38 | 26.3±2.2 | 3.6±2.8 | 0.4±0.9 | 9.4±0.9 | 74.9±9.7 | | -0.5±9.7 | |
| Hoffmann, 1994[^2^](#_ENREF_2) | | 24 weeks | Acarbose 100mg tid | 28 | 58.8±6.9 | 46 | 26.5±1.6 | 12.7±10.8 | -0.99±0.4 | 8.29±0.42 | / | | / | |
|  |  |  | Placebo | 30 | 56.9±6.7 | 40 | 26.8±1.5 | 12.1±10.8 | 0.11±0.3 | 8.29±0.37 | / | | / | |
| Josse, 2003[^34^](#_ENREF_34) | | 12 months | Acarbose 100mg tid | 93 | 69.7±0.5 | / | 28.3±0.4 | 5.8±0.7 | -0.3±0.1 | 7.4±0.1 | 79.4±1.3 | | / | |
|  |  |  | Placebo | 99 | 70.3±0.5 | / | 28.6±0.4 | 4.8±0.5 | 0.3±0.1 | 7.3±0.1 | 81.3±1.6 | | / | |
| Meneilly, 2000[^35^](#_ENREF_35) | | 12 months | Acarbose 100mg tid | 22 | 68±1 | / | 29.0±1.0 | / | -0.4±0.1 | 7.3±0.1 | / | | / | |
|  |  |  | Placebo | 23 | 70±1 | / | 28.0±1.0 | / | 0.4±0.2 | 7±0.2 | / | | / | |
| Rosenbaum, 2002[^36^](#_ENREF_36) | | 22 weeks | Acarbose 300mg qd | 20 | 59.8±8.2 | 30 | 30.3±2.9 | 6.8 | -0.8±1.98 | 6.4±1.7 | 75.1±11.6 | | / | |
|  |  |  | Placebo | 20 | 62±9.7 | 40 | 31.7±3.9 | 6.8 | 0±2.2 | 6.3±2.1 | 80.2±9.8 | | / | |
| Segal, 1997[^3^](#_ENREF_3) | | 24 weeks | miglitol | 40 | 61 | 55 | 28.6 | / | -0.60±1 | 7.95 | / | | -1.4±1 | |
|  |  |  | glibenclamide | 37 | 56 | 62.2 | 29.2 | / | -0.86±1 | 7.96 | / | | +1.4±1 | |
|  |  |  | PBO | 42 | 59 | 57.1 | 29.1 | / | 0.15±1 | 8.25 | / | | 0 | |
| Scott, 1999[^37^](#_ENREF_37) | | 16 weeks | Acarbose 100mg tid | 53 | 56±9 | 62 | 31.0±3.0 | 1.75±1.25 | -0.14±0.91 | 7.0±0.87 | 91±13 | | / | |
|  |  |  | Placebo | 52 | 57±8 | 65 | 29.0±3.0 | 2.17±1.42 | 0.25±1.15 | 6.89±0.85 | 84±12 | | / | |
| Wagner, 2006[^38^](#_ENREF_38) | | 12 weeks | Acarbose 100mg tid | 14 | 57(52-58) | 57.1 | 28.7  (26.3-29, 6) | 3.5(1-6) | -0.4±0.7 | 5.9±0.75 | 53.1 | | +0.2±1 | |
|  |  |  | Placebo | 17 | 54(50-58) | 82.3 | 28.7  (25.6-30.30) | 4(2-5) | -0.5±0.55 | 6.6±0.5 | 60.5 | | 0±1 | |
| **AGI versus Placebo, add-on therapy** | | | | | | | | | | |  | |  | |
| Bachmann, 2003[^39^](#_ENREF_39) | | 78 weeks | Acarbose100mg tid +SU | 164 | 63.8±7.1 | 47.6 | 29.0±3.1 | 8±13.3 | -0.54±0.32 | 9.42±0.66 | 80.7±11.4 | | / | |
|  |  |  | Placebo+SU | 166 | 63.3±7.2 | 43.3 | 29.0±2.9 | 8±12.5 | 0±0.73 | 9.38±0.73 | 81.6±11.8 | | / | |
| Chiasson, 1994[^24^](#_ENREF_24) | | 1 year | Acarbose 200mg qd +MET | 41 | / | / | 29.4±0.6 | / | -0.6±0.2 | 7.8±0.2 | / | | / | |
|  |  |  | Placebo+MET | 42 | / | / | 29.4±0.6 | / | 0.3±0.2 |  | / | | / | |
|  |  |  | Acarbose 200mg qd +SU | 52 | / | / | 27.8±0.4 | / | -0.6±0.2 | 8±0.2 | / | | / | |
|  |  |  | Placebo+SU | 51 | / | / | 27.8±0.4 | / | 0.3±0.2 |  | / | | / | |
|  |  |  | Acarbose 200mg qd +insulin | 41 | / | / | 30.2±0.5 | / | -0.5±0.2 | 7.7±0.2 | / | | / | |
|  |  |  | Placebo+insulin | 50 | / | / | 30.2±0.5 | / | -0.1±0.2 |  | / | | / | |
| Chiasson, 2001[^11^](#_ENREF_11) | | 36 weeks | Placebo+MET | 83 | 57.9±8.6 | 73.5 | 30.7±5.1 | 7.5±7.4 | -0.85±0.12 | 8.2±0.9 | 88.6±14.1 | | -0.69±0.27 | |
|  |  |  | Miglitol+MET | 76 | 58.9±7.9 | 77.6 | 29.5±3.8 | 6.1±5.5 | -1.39±0.11 | 8.3±0.8 | 91±15.5 | | -0.42±0.29 | |
| Halimi, 2000[^40^](#_ENREF_40) | | 6 months | Acarbose 100mg tid +MET | 59 | 56±9.2 | 47.4 | 30.1±3.3 | 9.5±7.4 | -0.7±1.2 | 8.6±1.1 | / | | / | |
|  |  |  | Placebo+MET | 70 | 55±10 | 62.8 | 29.7±3.3 | 9±7.5 | 0.2±1.3 | 8.5±1.1 | / | | / | |
| Kelley, 1998[^41^](#_ENREF_41) | | 24 weeks | Acarbose 100mg tid+insulin | 72 | 61.8 | 63 | 31 | 12.5 | -0.58±0.1 | 8.77 | 91.4 | | -0.58±1 | |
|  |  |  | Placebo+insulin | 73 | 60.8 | 48 | 31.1 | 12.3 | 0.11±0.1 | 8.69 | 88.8 | | 0.11±1 | |
| Johnston, 1998 [^42^](#_ENREF_42) | | 1 year | Miglitol 100mg tid±SU | 204 | 55±1 | 50 | 31.9±0.1 | 5.1±0.5 | -0.21±0.15 | 8.69±0.14 | / | | / | |
|  |  |  | Placebo±SU | 105 | 56.9±1.3 | 51 | 32.0±0.9 | 4.5±0.7 | 0.53±0.21 | 8.62±0.18 | / | | / | |
| Johnston, 1998-2[^43^](#_ENREF_43) | | 1 year | Miglitol 200mg tid±SU | 220 | 52.9 | / | 31.8 | 5.7 | -0.26±1 | 8.7 | 85.8 | | / | |
|  |  |  | Placebo±SU | 120 | 53.9 | / | 30.6 | 4.8 | 0.57±1 | 8.53 | 82.9 | | / | |
| Lam KSL, 1998[^44^](#_ENREF_44) | | 24 weeks | Acarbose 100mg tid+SU+met | 45 | 57.8±1.3 | 44.4 | 24.8±0.5 | 10.2±0.7 | -0.5±0.2 | 9.5±0.1 | / | | -0.54±0.32 | |
|  |  |  | Placebo+SU+met | 44 | 56.9±1.3 | 43.2 | 24.1±0.4 | 10.1±0.8 | 0.1±0.2 | 9.4±0.1 | / | | +0.42±0.29 | |
| Mitrakou,1998[^45^](#_ENREF_45) | | 24 weeks | Miglitol 100mg tid+insulin | 60 | 57.4±5.6 | 48.3 | 24.4±3.1 | 8.5±4.5 | -1.6±0.7 | 9.9±0.5 | 67.3±6.2 | | +0.6±6 | |
|  |  |  | Placebo+ insulin | 60 | 57.4±5.8 | 61.7 | 24.5±3.4 | 7.9±3.2 | -0.3±0.6 | 9.9±0.4 | 68.9±7.3 | | +0.2±7.5 | |
| Phillips, 2003[^46^](#_ENREF_46) | | 24 weeks | Acarbose 100mg bid +MET | 40 | 58.37±10.7 | 65 | 30.75±2.96 | 5.32±4.55 | -0.16±0.18 | 8.05±0.89 | 89.77±12.73 | | -1.32±2.37 | |
|  |  |  | Placebo+MET | 43 | 62.39±8.02 | 76.7 | 30.09±2.85 | 6.06±5.32 | 0.86±0.16 | 7.82±0.83 | 87.88±11.7 | | -0.43±2.9 | |
| Schnell, 2007[^47^](#_ENREF_47) | | 20 weeks | Acarbose 100mg tid+insulin | 82 | 61.5±8.9 | / | 30.4±4.2 | 11.5±7 | -2.31±1.61 | 9.8±1.5 | 85.44±13.64 | | -0.25±20.73 | |
|  |  |  | Placebo+insulin | 81 | 62.3±7.4 | / | 29.9±4.5 | 9.6±5.1 | -1.81±1.11 | 9.4±1 | 84.41±13.18 | | 10.36±13.68 | |
| Standl, 1999[^48^](#_ENREF_48) | | 24 weeks | Acarbose 200mg tid+insulin | 24 | 59.3±8.5 | / | 25.2±2.2 | 11.5±6.8 | -2.4±1.0 | 10.9±1 | 70.5±11.7 | | +1.7±11.7 | |
|  |  |  | Placebo+insulin | 24 | 62.9±9.4 | / | 24.1±2.0 | 12.2±5.7 | -2.4±1.0 | 11.0±1.2 | 66.7±10.8 | | +1.5±10.8 | |
| Standl, 2001[^49^](#_ENREF_49) | | 24 weeks | Miglitol 100mg tid+SU+MET | 65 | 62±8 | 50.8 | 27.7±3.7 | 8 | -0.55±1.02 | 8.83±0.85 | 79±13 | | / | |
|  |  |  | Placebo+ SU+MET | 68 | 61±8 | 54.4 | 27.9±3.5 | 9 | -0.2±1.07 | 8.84±0.66 | 82±15 | | / | |
| Van Gaal, 2001[^50^](#_ENREF_50) | | 32 weeks | Miglitol 100mg tid+MET | 77 | 57.9±10 | 41.5 | 30.0±4.0 | 6 | -0.21±1.13 | 8.5±1 | / | | -2.5±3.8 | |
|  |  |  | Placebo+ MET | 75 | 57.9±8.5 | 49.3 | 29.7±3.9 | 6 | 0.22±1.17 | 8.4±1 | / | | -0.7±2.5 | |
| **Author , year** | | **Study duration** | **Treatment group** | **No. of patients** | **Age (years)** | **Men (%)** | **BMI (kg/m2)** | **DM duration**  **(years)** | **HbA1c change (%)** | **Baseline HbA1c (%)** | **Baseline Weight**  **(kg)** | | **Weight Change**  **(kg)** | |
| **TZD versus Placebo, monotherapy** | | | | | | | | | | |  | |  | |
| Carey, 2002[^51^](#_ENREF_51) | | 16 weeks | rosiglitazone 8mg/day | 16 | 54.2±11.1 | 87.5 | 29.8±4.0 | 3.3±4.5 | -0.7±0.7 | 7.8±1.3 | 93.9±14.1 | | 2.1±2.0 | |
|  |  |  | placebo | 17 | 57.9±10.7 | 76.5 | 31.3±3.6 | 3.1±3.3 | 0.4±1.0 | 7.1±1.4 | 89.5±12.5 | | 0.1±1.8 | |
| Chou, 2012[^52^](#_ENREF_52) | | 26 weeks | rivoglitazone 1.0mg/day | 274 | 55.0±10.51 | 48.2 | 29.7±5.63 | 5.0±5.26 | -0.4±0.05 | 7.7±0.53 | 80.5±19.65 | | +2.3±19.65 | |
|  |  |  | rivoglitazone 1.5mg/day | 750 | 55.1±10.59 | 50.9 | 29.6±5.27 | 4.3±4.40 | -0.7±0.03 | 7.7±0.57 | 80.6±17.67 | | +3.4±17.67 | |
|  |  |  | pioglitazone 45mg/day | 751 | 55.0±10.84 | 53 | 30.0±5.80 | 4.4±4.99 | -0.6±0.03 | 7.7±0.58 | 81.6±19.59 | | +2.7±19.59 | |
|  |  |  | placebo | 137 | 55.4±12.32 | 48.9 | 30.1±5.43 | 4.9±6.13 | 0.2±0.06 | 7.7±0.54 | 82±19.73 | | -0.8±19.73 | |
| Ebeling, 1999[^53^](#_ENREF_53) | | 16 weeks | Troglitazone 400mg qd | 15 | 62.6±2.2 | 33.3 | 32.3±1.3 | 15.9±2.1 | -1.2±0.3 | 8.7±0.3 | / | | / | |
|  |  |  | placebo | 12 | 63.5±2.8 | 50 | 33.1±1.0 | 14.3±1.9 | -0.1±0.3 | 8.8±0.3 | / | | / | |
| Ebeling, 2001[^1^](#_ENREF_1) | | 6 months | Pioglitazone 30mg qd | 9 | / | / | 30.5±1.3 | / | -1.1±0.5 | 9.1±0.3 | / | | / | |
|  |  |  | glibenclamide | 10 | / | / | 30.2±1.7 | / | -1.2±0.3 | 8.9±0.3 | / | | / | |
|  |  |  | PBO | 10 | / | / | 31.9±1.5 | / | -0.2±0.3 | 8.6±0.2 | / | | / | |
| Fonseca, 1998[^54^](#_ENREF_54) | | 26 weeks | Troglitazone 600mg qd | 18 | 60.4±5.9 | 44.4 | 37.3±9.2 | / | 0.3±2 | 9.5±2 | / | | / | |
|  |  |  | placebo | 8 | 52.6±7.5 | 37.5 | 39.6±13.4 | / | 0.8±1.3 | 10.1±1.43 | / | | / | |
| Gastaldelli, 2006[^55^](#_ENREF_55) | | 12 weeks | rosiglitazone 8mg/day | 13 | 53±2 | 53.8 | 29.3±1.2 | 4±2 | -1.1±0.5 | 8.6±0.5 | / | | / | |
|  |  |  | PBO | 13 | 56±2 | 61.5 | 30.2±1.0 | 3±1 | 0.4±0.4 | 8.2±0.4 | / | | / | |
| Gastaldelli, 2007-1[^56^](#_ENREF_56) | | 4 months | rosiglitazone 8mg/day | 12 | 55±3 | 50 | 29.2±1.3 | 4±2 | -1.4±0.5 | 8.7±0.5 | / | | / | |
|  |  |  | PBO | 12 | 56±2 | 66.7 | 29.8±1.2 | 2±1 | 0.6±0.5 | 8.1±0.4 | / | | / | |
| Gastaldelli, 2009[^57^](#_ENREF_57) | | 16 weeks | Pioglitazone 45mg/d | 17 | 61±1 | 59.3 | 28.7±0.7 | / | -0.44±0.27 | 7.0±0.2 | 78±2 | | 1.2±0.6 | |
|  |  |  | PBO | 10 | 61±1 | 59.3 | 28.7±0.7 | / | 0.14±0.35 | 7.0±0.2 | 78±2 | | 0.1±0.8 | |
| Haffner, 2002[^58^](#_ENREF_58) | | 26 weeks | rosiglitazone 4mg/day | 126 | 60.7±9.3 | 53.2 | 30.3±4.1 | 4.7±6.1 | -0.6±1.2 | 8.8±1.4 | / | | / | |
|  |  |  | rosiglitazone 8mg/day | 136 | 60.4±9.3 | 47.8 | 29.5±3.8 | 4.9±5.2 | -0.9±1.2 | 8.6±1.5 | / | | / | |
|  |  |  | PBO | 95 | 59.8±10.5 | 61.1 | 30.1±3.9 | 4.5±4.8 | 0.6±1.1 | 8.7±1.5 | / | | / | |
| Hällsten, 2002[^15^](#_ENREF_15) | | 26 weeks | Rosiglitzone 4mg bid | 14 | 58.6±2.0 | 71.4 | 29.3±1.0 | / | -0.3±0.2 | 6.8±0.2 | / | | / | |
|  |  |  | metformin | 13 | 57.8±2.2 | 61.5 | 29.9±1.1 | / | -0.7±0.2 | 6.9±0.2 | / | | / | |
|  |  |  | PBO | 14 | 57.7±1.9 | 71.4 | 30.3±1.2 | / | -0.2±0.1 | 6.3±0.1 | / | | / | |
| Herz, 2003[^59^](#_ENREF_59) | | 16 weeks | Pioglitazone 30mg qd | 99 | 59.9±11.0 | 59.6 | 31.7±4.6 | 1.9±3.2 | -0.8±1 | / | 86.3±17.4 | | 0.35 | |
|  |  |  | Pioglitazone 45mg qd | 99 | 58.1±11.0 | 52.5 | 30.8±5.1 | 1.7±3.6 | -0.8±1 | / | 86.6±15.9 | | 0.82 | |
|  |  |  | PBO | 99 | 58.0±10.7 | 49.5 | 31.7±4.5 | 1.5±2.5 | -0.2±1.0 | / | 84.1±16.8 | | -1.58 | |
| Iwamoto, 1996[^60^](#_ENREF_60) | | 12 weeks | troglitazone 400mg/day | 136 | 54.6±10.1 | 50.7 | 24.1±3.5 | 6.3±4.4 | -0.5±1.7 | 8.61±1.51 | 61±11 | | 0.6±1.6 | |
|  |  |  | PBO | 126 | 57.4±9.3 | 53.2 | 24.7±3.4 | 7.5±5.4 | 0.1±1.6 | 8.51±1.46 | 61.8±10.4 | | -0.4±1.2 | |
| Juhl, 2003[^61^](#_ENREF_61) | | 13 weeks | rosiglitazone 4mg bid | 10 | 54±9 | 90 | 30.0±2.7 | / | -0.3±1.2 | 7.0±1.4 | 92±7 | | +2±8 | |
|  |  |  | PBO | 10 | 54±9 | 60 | 31.7±1.9 | / | 0.1±1.1 | 6.8±1.0 | 95±10 | | -0.3±10 | |
| Khan, 2006[^62^](#_ENREF_62) | | 26 weeks | pioglitazone 15mg | 22 | 56.4±9.95 | 63.6 | 31.8±3.67 | / | -0.70±0.279 | 8.46±0.309 | 93.7±18.09 | | / | |
|  |  |  | pioglitazone 30mg | 22 | 52.7±8.95 | 68.2 | 32.3±4.48 | / | -0.91±0.282 | 8.45±0.312 | 95.3±14.96 | | / | |
|  |  |  | pioglitazone 45mg | 23 | 56.7±7.17 | 60.9 | 30.7±3.05 | / | -1.63±0.297 | 9.54±0.326 | 90.3±13.06 | | / | |
|  |  |  | PBO | 21 | 54.8±8.65 | 28.6 | 32.0±4.23 | / | 0.66±0.289 | 8.62±0.323 | 89.3±11.25 | | / | |
| Kong, 2011[^63^](#_ENREF_63) | | 12 weeks | rivoglitazone 0.5mg | 35 | 53.9±7.6 | 54.3 | 26.05±4.51 | 4.39±3.66 | -0.11±0.85 | 7.46±0.72 | 67.5±13.83 | | / | |
|  |  |  | rivoglitazone 1.0mg | 35 | 53.2±7.8 | 57.1 | 25.6±3.72 | 4.87±3.14 | -0.22±0.74 | 7.45±0.72 | 65.34±13.11 | | / | |
|  |  |  | rivoglitazone 1.5mg | 34 | 52.4±9.3 | 55.9 | 25.97±4.17 | 4.36±3.74 | -0.17±0.87 | 7.4±0.70 | 68.91±13.13 | | / | |
|  |  |  | pioglitazone 30mg | 37 | 53.6±7.6 | 56.8 | 24.86±3.26 | 5.59±4.60 | -0.06±0.86 | 7.49±0.82 | 64.09±10.79 | | / | |
|  |  |  | PBO | 32 | 54.0±8.5 | 59.4 | 25.53±4.03 | 5.85±3.89 | 0.61±0.99 | 7.35±0.62 | 67.71±13.7 | | / | |
| Kumar, 1996[^64^](#_ENREF_64) | | 12 weeks | troglitazone 200mg qd | 48 | 59 | 64.6 | 28.5±4.1 | 5 | -0.3±1 | 7.6 | / | | / | |
|  |  |  | troglitazone 400mg qd | 41 | 58 | 58.5 | 28.4±3.9 | 7 | 0.3±1 | 7.1 | / | | / | |
|  |  |  | troglitazone 600mg qd | 47 | 60 | 57.4 | 28.6±5.3 | 6 | 0±1 | 7.2 | / | | / | |
|  |  |  | troglitazone 800mg qd | 50 | 56 | 64 | 28.3±4.4 | 5 | 0±1 | 7 | / | | / | |
|  |  |  | troglitazone 200mg bid | 45 | 57 | 68.9 | 29.3±4.3 | 6 | -0.2±1 | 7.3 | / | | / | |
|  |  |  | troglitazone 400mg bid | 49 | 56 | 57.1 | 27.7±4.0 | 6 | 0.2±1 | 6.9 | / | | / | |
|  |  |  | PBO | 49 | 57 | 73.5 | 28.9±4.6 | 7 | 0.8±1 | 7.2 | / | | / | |
| Lautamäki, 2005[^65^](#_ENREF_65) | | 16 weeks | Rosiglitazone 8mg qd | 27 | 64.1±7.8 | 70.4 | 29.6±4.7 | 6.7±6.4 | -0.4±0.9 | 7.3±0.9 | 85.3±17.4 | | / | |
|  |  |  | PBO | 27 | 63.2±7.4 | 70.4 | 29.6±3.4 | 6.8±5.9 | 0.2±1.0 | 7.1±0.9 | 89.1±14.3 | | / | |
| Miyazaki, 2001[^66^](#_ENREF_66) | | 12 weeks | rosiglitazone 8mg/day | 15 | / | / | 30.0±1.1 | / | -1.3±0.3 | 8.7±0.4 | 86±4 | | 3.7±0.8 | |
|  |  |  | PBO | 14 | / | / | 30.1±1.0 | / | 0.5±0.3 | 8.3±0.4 | 87±5 | | 0±0.4 | |
| Miyazaki, 2001[^67^](#_ENREF_67) | | 16 weeks | pioglitazone 45mg/day | 12 | / | / | 28.7±1.1 | / | -1.7±0.3 | 8.9±0.3 | 84.8±3.6 | | 3.6±1.4 | |
|  |  |  | PBO | 11 | / | / | 29.5±1.3 | / | 0±0.2 | 7.9±0.3 | 81.4±5 | | 0.3±0.4 | |
| Miyazaki, 2002[^68^](#_ENREF_68) | | 26 weeks | pioglitazone 7.5mg | 13 | 51±3 | 76.9 | 31.3±1.2 | / | 0.3±0.4 | 8.9±0.4 | 93±5 | | 0.2±0.5 | |
|  |  |  | pioglitazone 15mg | 12 | 57±4 | 66.7 | 30.8±1.3 | / | -0.1±0.4 | 8.0±0.3 | 93±5 | | 2±0.9 | |
|  |  |  | pioglitazone 30mg | 11 | 51±2 | 72.7 | 32.2±1.6 | / | -0.8±0.3 | 8.5±0.5 | 97±4 | | 3±1.1 | |
|  |  |  | pioglitazone 45mg | 11 | 55±2 | 45.5 | 30.4±1.0 | / | 1.8±0.4 | 9.1±0.3 | 86±3 | | 4.5±0.7 | |
|  |  |  | PBO | 11 | 58±3 | 27.3 | 32.8±1.6 | / | 1.2±0.5 | 8.6±0.5 | 90±4 | | -0.4±1,4 | |
| Natali, 2004[^69^](#_ENREF_69) | | 16 weeks | metformin | 28 | 58±10 | 78.6 | 28.0±3.5 | 6.3±5.3 | -0.33±1.1 | 7.8±1.1 | 77.3±12.5 | | -0.6±0.4 | |
|  |  |  | Rosiglitazone 8mg qd | 24 | 59±7 | 91.7 | 27.6±2.8 | 6.5±4.5 | 0.09±1.2 | 7.2±1.2 | 80.4±10.1 | | 0.5±0.5 | |
|  |  |  | PBO | 22 | 58±9 | 81.8 | 30.2±3.1 | 3.4±3.4 | 1.3±0.8 | 7.6±0.8 | 86.9±10.5 | | -0.3±0.8 | |
| Oz Gul, 2008[^70^](#_ENREF_70) | | 12 weeks | pioglitazone 30mg/day | 14 | / | / | 29.3±2.9 | / | -1.22±1.6 | 7.82±1.7 | / | | / | |
|  |  |  | rosiglitazone 4mg/day | 11 | / | / | 28.3±4.09 | / | -0.8±1 | 7.0±1.07 | / | | / | |
|  |  |  | PBO | 10 | / | / | 29.2±2.3 | / | 0.06±1.0 | 6.39±1.1 | / | | / | |
| Oz Gul, 2010[^71^](#_ENREF_71) | | 12 weeks | pioglitazone 30mg/day | 19 | / | / | 29.3±3.0 | / | -1.1±1.4 | 7.6±1.5 | / | | / | |
|  |  |  | rosiglitazone 4mg/day | 20 | / | / | 29.6±4.8 | / | -1.1±1.3 | 7.3±1.3 | / | | / | |
|  |  |  | PBO | 21 | / | / | 29.6±4.1 | / | -0.1±0.9 | 7.3±0.9 | / | | / | |
| Patel, 1999[^72^](#_ENREF_72) | | 12 weeks | rosiglitazone 0.05mg bid | 74 | 56.7±12.33 | 66.2 | 29.4±3.76 | 4.9 | 0.6±0.14 | 9.1 | / | | / | |
|  |  |  | rosiglitazone 0.25mg bid | 72 | 55.8±11.75 | 70.8 | 28.6±4.11 | 6.7 | 0.6±0.14 | 8.9 | / | | / | |
|  |  |  | rosiglitazone 1.0mg bid | 79 | 59.8±9.51 | 64.6 | 29.5±4.06 | 4.4 | 0.1±0.13 | 9 | / | | / | |
|  |  |  | rosiglitazone 2.0mg bid | 80 | 59.7±10.0 | 68.8 | 28.4±4.09 | 5.8 | -0.1±0.13 | 9 | / | | / | |
|  |  |  | PBO | 75 | 56.8±11.50 | 69.3 | 28.9±3.98 | 4.2 | 0.3±0.13 | 9.1 | / | | / | |
| Phillips, 2001[^73^](#_ENREF_73) | | 16 weeks | rosiglitazone 4mg Qd | 181 | 57.5±9.9 | 58.6 | 29.9±4.1 | 5.4±6.1 | 0±1.6 | 8.9±1.6 | / | | / | |
|  |  |  | rosiglitazone 2mg bid | 186 | 56.8±9.4 | 59.1 | 30.0±4.2 | 5.5±4.9 | -0.1±1.5 | 8.9±1.5 | / | | / | |
|  |  |  | rosiglitazone 8mg Qd | 181 | 58.9±9.9 | 65.7 | 30.0±4.3 | 6.1±6.7 | -0.3±1.5 | 8.9±1.5 | / | | / | |
|  |  |  | rosiglitazone 4mg bid | 187 | 56.5±9.7 | 65.2 | 29.9±4.3 | 5.9±6.1 | -0.7±1.5 | 9.0±1.5 | / | | / | |
|  |  |  | PBO | 173 | 57.7±9.2 | 68.8 | 29.1±4.2 | 6.6±6.9 | 0.8±1.5 | 8.9±1.5 | / | | / | |
| Raskin, 2000[^74^](#_ENREF_74) | | 8 weeks | rosiglitazone 2mg bid | 73 | 58.47±9.80 | 61.6 | 30.15±4.68 | 5.6±5.93 | 0.4±1.8 | 8.7±1.44 | / | | / | |
|  |  |  | rosiglitazone 4mg bid | 66 | 57.02±10.0 | 60.6 | 30.49±3.76 | 4.0±4.70 | / | 8.9±1.45 | / | | / | |
|  |  |  | rosiglitazone 6mg bid | 76 | 58.61±10.05 | 60.5 | 30.02±4.38 | 6.0±5.81 | / | 8.7±1.49 | / | | / | |
|  |  |  | PBO | 69 | 60.06±9.39 | 59.4 | 30.44±4.15 | 5.6±5.19 | 1.0±2.0 | 8.7±1.63 | / | | / | |
| Rosenblatt, 2001[^75^](#_ENREF_75) | | 16 weeks | pioglitazone 30mg Qd | 101 | 53.8±10.0 | 50.5 | 31.5±4.7 | / | -0.6±0.17 | 10.65±1.77 | 89.8±18.0 | | +1.35±18.0 | |
|  |  |  | PBO | 96 | 55.2±10.0 | 56.2 | 30.7±5.0 | / | 0.76±0.17 | 10.42±1.70 | 87.2±18.4 | | -1.87±18.4 | |
| Rosenstock, 2002[^76^](#_ENREF_76) | | 16 weeks | troglitazone 600mg/day | 150 | 58 | 59 | 21-38 | 0.1-33.4 | -0.8±1 | 8.4±1.1 | / | | / | |
|  |  |  | nateglinide 120mg/day | 151 | 57 | 63 | 19-37 | 0-20.4 | -0.6±1 | 8.1±1.0 | / | | / | |
|  |  |  | nateglinide 120mg/day  +troglitazone 600mg/day | 150 | 59 | 61 | 21-45 | 0.3-26.2 | -1.7±1.3 | 8.3±1.3 | / | | / | |
|  |  |  | PBO | 148 | 58 | 59 | 20-38 | 0.2-37.9 | 0.5±1.0 | 8.2±1.2 | / | | / | |
| Scherbaum, 2002[^77^](#_ENREF_77) | | 26 weeks | pioglitazone 15mg Qd | 89 | 58 | 62.9 | 29.9 | 5.4 | -0.92±1.50 | 9.33 | 87.2 | | / | |
|  |  |  | pioglitazone 30mg Qd | 78 | 59.6 | 41 | 29.3 | 4.6 | -1.05±1.25 | 9.06 | 82.0 | | / | |
|  |  |  | PBO | 84 | 59.1 | 56 | 29.2 | 5.6 | -0.34±0.98 | 8.75 | 84.8 | | / | |
| Tan, 2005[^78^](#_ENREF_78) | | 24 weeks | rosiglitazone 4mg bid | 12 | / | / | 30.83±1.11 | / | -0.5±0.2 | 7.17±0.26 | / | | / | |
|  |  |  | PBO | 12 | / | / | 30.8±1.04 | / | -0.1±0.2 | 7.52±0.38 | / | | / | |
| Truitt, 2010[^79^](#_ENREF_79) | | 26 weeks | rivoglitazone 1mg Qd | 87 | 55.4±10.9 | 52.9 | 32.9±6.0 | 6.2±6.4 | 0.02±0.13 | 8.00±0.81 | 93.3±20.7 | | 1.2±20.7 | |
|  |  |  | rivoglitazone 2mg Qd | 85 | 55.0±10.8 | 56.5 | 33.1±6.4 | 5.2±4.5 | -0.43±0.13 | 8.05±0.87 | 96.3±18.7 | | 3.0±18.7 | |
|  |  |  | rivoglitazone 3mg Qd | 86 | 56.4±10.8 | 52.3 | 33.0±6.5 | 6.7±7.3 | -0.54±0.14 | 8.23±1.02 | 95.8±23.5 | | 4.1±23.5 | |
|  |  |  | pioglitazone 45mg Qd | 91 | 56.6±10.1 | 58.2 | 32.9±5.7 | 6.6±7.5 | -0.03±0.13 | 7.98±0.83 | 94.9±21.5 | | 2.1±21.5 | |
|  |  |  | PBO | 92 | 55.3±9.3 | 51.1 | 32.2±5.8 | 6.7±5.6 | 0.56±0.13 | 8.21±0.98 | 92.9±20.4 | | -1.4±20.4 | |
| Viljanen, 2005[^18^](#_ENREF_18) | | 26 weeks | Rosiglitazone 4mg bid | 14 | 58.6±7.7 | 64.3 | 29.3±4.0 | 6.8±0.9 | -0.3±0.9 | 6.8±0.9 | / | | / | |
|  |  |  | Metformin 1g bid | 12 | 57.8±8.7 | 58.3 | 29.6±4.0 | 6.9±0.9 | -0.3±0.9 | 6.9±0.9 | / | | / | |
|  |  |  | PBO | 11 | 58.7±8.3 | 81.8 | 29.8±4.1 | 6.2±0.7 | -0.1±0.7 | 6.2±0.7 | / | | / | |
| Wallace, 2004[^80^](#_ENREF_80) | | 12 weeks | Pioglitazone 45mg qd | 19 | 61.4±6.3 | 73.7 | 29.8±4.5 | 2.6 | -0.3±0.1 | 6.7±0.9 | 90.7±3.6 | | 0.7±0.6 | |
|  |  |  | PBO | 11 | 62.6±10.0 | 72.7 | 28.9±2.8 | 2.5 | 0.3±0.1 | 6.7±0.9 | 85.2±4.3 | | 1.1±0.5 | |
| **TZD versus Placebo, add-on therapy** | | | | | | | | | | |  | |  | |
| Barnett, 2003[^81^](#_ENREF_81) | | 26 weeks | rosiglitazone 8mg/day  +sulphonylurea | 84 | 54.3 | 80 | 26.8 | 6.5 | -1.16±1.3 | 9.21±1.27 | / | | / | |
|  |  |  | placebo+sulphonylurea | 87 | 54.1 | 75 | 26.4 | 6.5 | 0.26±1.3 | 9.06±1.3 | / | | / | |
| Berhanu, 2007[^82^](#_ENREF_82) | | 20 weeks | Pioglitazone 45mg qd+insulin±metformin | 110 | 52.9±11.33 | 43.6 | 30.7±6.09 | 7.7±6.15 | -1.6±0.11 | 8.4±0.13 | / | | +4.39±1 | |
|  |  |  | placebo+insulin±metformin | 112 | 52.5±11.07 | 41.1 | 31.8±6.2 | 8.5±5.43 | -1.4±0.11 | 8.6±0.13 | / | | +2.42±1 | |
| Bertrand, 2010[^83^](#_ENREF_83) | | 12 months | Rosiglitazone 4mg bid | 98 | 64.2±7.3 | 92 | 30.2±4.2 | 7.8±6.4 | -0.5±1.2 | 6.9±1.3 | 85.9±14 | | 2.9±14 | |
|  |  |  | placebo | 95 | 65.9±6.9 | 92 | 29.5±4.6 | 8.4±6.9 | 0.1±0.9 | 6.9±0.8 | 83.9±15.1 | | 0.4±15 | |
| Brackenridge, 2009[^84^](#_ENREF_84) | | 3 months | rosiglitazone 8mg±metformin | 8 | 66.5±2.51 | 50 | 30.0±1.5 | 4.4±1.03 | -0.4±0.3 | 6.9±0.3 | 92±6.52 | | +0.6±6.52 | |
|  |  |  | pioglitazone 30mg±metformin | 8 | 61.0±3.93 | 87.5 | 30.8±1.26 | 4.0±0.8 | -0.7±0.2 | 7.5±0.21 | 96.4±3.62 | | +1.9±3.62 | |
|  |  |  | placebo±metformin | 8 | 60.8±3.45 | 87.5 | 32.0±1.56 | 2.9±0.4 | 0.2±0.2 | 6.6±0.14 | 103.9±5.61 | | +0.5±5.61 | |
| Buras, 2005[^85^](#_ENREF_85) | | 12 weeks | troglitazone 600mg | 33 | 58±9 | 60.6 | 30.9±5.3 | 8±8 | -0.6±0.75 | 7.6±1.4 | / | | 2.3±2.5 | |
|  |  |  | placebo | 39 | 57±9 | 66.7 | 32.6±5.0 | 8±9 | 0.1±0.45 | 7.9±1.4 | / | | 0.5±1.25 | |
| Buse, 1998[^86^](#_ENREF_86) | | 26 weeks | troglitazone 200mg | 75 | 58±9 | 59 | 34.4±6.2 | / | / | 9.5±1.7 | 98.9±18.1 | | -0.03±0.44 | |
|  |  |  | troglitazone 400mg | 76 | 58±10 | 50 | 34.8±6.7 | / | -0.4±1.4 | 9.0±1.4 | 98.9±20.2 | | +0.6±0.44 | |
|  |  |  | placebo | 71 | 57±11 | 49 | 34.5±7.2 | / | -0.1±1.4 | 9.0±1.4 | 98.9±22.4 | | -0.9±0.44 | |
| Charpentier, 2009[^87^](#_ENREF_87) | | 7 months | pioglitazone 30/45mg | 142 | 60.2±9.3 | 64.6 | 29.1±3.3 | 12.5±9.0 | -0.83±0.7 | 8.1±0.7 | 82 | | +1.8±1 | |
|  |  |  | placebo | 147 | 59.2±9.6 | 66.2 | 29.2±3.1 | 12.1±7.9 | +0.22±0.6 | 8.2±0.6 | 82.5 | | -0.2±1 | |
| Dailey, 2004[^88^](#_ENREF_88) | | 24 weeks | Rosiglitazone 8mg qd＋glyburide/metformin | 181 | 57±9 | 58 | 32±5 | 9±7 | -0.9±0.9 | 8.1±0.9 | 93±18 | | 3±18 | |
|  |  |  | placebo＋glyburide/metformin | 184 | 57±10 | 61 | 32±5 | 9±6 | 0.1±0.8 | 8.1±0.8 | 93±18 | | 0.03±18 | |
| Davidson, 2007[^89^](#_ENREF_89) | | 24 weeks | Rosiglitazone 8mg qd+glyburide | 117 | 52±11.9 | 45.3 | 31.3±5.7 | 6.0±5.2 | -1.2±0.2 | 9.2±1.3 | 86.3±18.8 | | 4.3±3.5 | |
|  |  |  | placebo+ glyburide | 116 | 53±10.4 | 48.3 | 31.9±5.6 | 6.2±5.3 | 0.14±0.2 | 9.4±1.4 | 88.3±19.4 | | 0.1±2.4 | |
| Derosa, 2008[^90^](#_ENREF_90) | | 6 months | Rosiglitazone 8mg qd+metformin | 56 | 55±4 | 46.4 | 28.6±1.9 | 3±1 | -1.4±0.6 | 7.8±0.7 | / | | / | |
|  |  |  | placebo+metformin | 61 | 54±3 | 47.5 | 28.4±1.7 | 4±1 | -0.6±0.8 | 8.0±0.9 | / | | / | |
| Einhorn, 2000[^91^](#_ENREF_91) | | 16 weeks | Pioglitazone 30mg qd+metformin | 168 | 55.5±10.34 | 54.8 | 32.11±5.3 | 9.86±1.4 | -0.63±1.4 | / | / | | +0.95 | |
|  |  |  | PBO+metformin | 160 | 55.7±9.92 | 60 | 32.12±5.5 | 9.75±1.3 | 0.2±1.3 | / | / | | -1.36 | |
| Fonseca, 2000[^92^](#_ENREF_92) | | 26 weeks | metformin 2.5g/d+placebo | 113 | 58.8±9.2 | 74.3 | 30.3±4.4 | 7.3±5.7 | 0.45±1.2 | 8.6±1.3 | / | | / | |
|  |  |  | metformin 2.5g/d+RSG 4mg/d | 116 | 57.5±10.5 | 62.1 | 30.2±4.2 | 7.5±6.3 | -0.56±1.3 | 8.9±1.3 | / | | / | |
|  |  |  | metformin 2.5g/d+RSG 8mg/d | 110 | 58.3±8.8 | 68.2 | 29.8±3.9 | 8.3±6.3 | -0.78±1.5 | 8.9±1.5 | / | | / | |
| Galle, 2012[^93^](#_ENREF_93) | | 6 months | pioglitazone 30mg+insulin | 20 | 68.9±6.8 | 70 | 31.5±4.0 | 13.8±9.8 | -0.6±0.87 | 7.4±0.9 | / | | / | |
|  |  |  | placebo+insulin | 19 | 69.6±9.4 | 68.4 | 30.3±4.6 | 12.4±8.2 | 0.21±1.1 | 7.7±0.9 | / | | / | |
| Gastaldelli, 2007-2[^56^](#_ENREF_56) | | 4 months | pioglitazone 45mg/day+SU | 10 | 55±4 | 50 | 28.9±1.3 | 6±2 | -2±0.6 | 9.3±0.4 | / | | / | |
|  |  |  | PBO+SU | 10 | 55±4 | 40 | 29.9±1.4 | 5±2 | 0.9±0.5 | 8.3±0.4 | / | | / | |
| Gómez-Perez, 2002[^94^](#_ENREF_94) | | 26 weeks | Met+Rosi 4mg bid | 36 | 54.2±9.3 | 19.4 | 27.6±3.2 | 10.7±7 | -1.2±1 | / | / | | / | |
|  |  |  | Met+PBO | 34 | 53.4±7.5 | 29.4 | 28.5±3.9 | 9.1±5.6 | 0.3±1 | / | / | | / | |
| Gram, 2011[^20^](#_ENREF_20) | | 2 years | PBO+NPH | 46 | 55.8±7.7 | 71.7 | 34.0±6.0 | 7.3±4.3 | -0.4±1.4 | 8.7±1.3 | 100.2±19.8 | | +5.3±20 | |
|  |  |  | metformin+NPH | 45 | 55.4±8.5 | 57.8 | 35.7±6.4 | 8.2±4.0 | -1.3±1.3 | 8.9±1.2 | 105.1±17.7 | | 2.9±20 | |
|  |  |  | rosiglitazone+NPH | 46 | 57.3±8.9 | 60.9 | 34.0±5.7 | 9.2±6.9 | -0.8±1.6 | 8.7±1.2 | 100.9±16.5 | | 7.6±20 | |
|  |  |  | metformin+rosiglitazone  +NPH | 46 | 57.3±8.2 | 65.2 | 34.4±7.0 | 8.1±5.3 | -1.7±1.1 | 8.5±1.1 | / | | / | |
|  |  |  | PBO+ASP | 48 | 57.1±8.5 | 47.9 | 33.7±5.0 | 9.1±5.5 | -0.6±1.2 | 8.5±1.2 | 98.3±16.6 | | 6.1±19 | |
|  |  |  | metformin+ASP | 45 | 56.1±8.2 | 62.2 | 33.7±6.1 | 8.7±4.5 | -1.2±1.2 | 8.5±1.2 | 100.5±17.9 | | 3.9±20 | |
|  |  |  | rosiglitazone+ASP | 47 | 56.1±8.3 | 57.4 | 32.7±4.7 | 9.4±6.3 | -1.2±1 | 8.3±1.0 | 95.6±14.6 | | 10.4±20 | |
|  |  |  | metformin+rosiglitazone  +ASP | 48 | 55.3±9.1 | 70.8 | 32.9±4.4 | 9.0±5.8 | -1.5±1.2 | 8.5±1.2 | / | | / | |
| Grey, 2012[^95^](#_ENREF_95) | | 6 months | pioglitazone 30mg daily | 10 | 61.9±10.0 | 60 | 31.2±4.7 | / | -0.28±0.3 | 7.6±2.1 | 90.1±14.9 | | / | |
|  |  |  | PBO | 10 | 57.9±15.2 | 50 | 33.2±4.1 | / | -0.04±0.4 | 7.1±1.0 | 91.7±12.8 | | / | |
| Henriksen, 2011[^96^](#_ENREF_96) | | 26 weeks | balaglitazone 10mg+insulin | 97 | 61.0±8.8 | 72 | 34.1±6.2 | 13.2±6.2 | -0.29±0.18 | 8.6±1.4 | / | | +3.5±1 | |
|  |  |  | balaglitazone 20mg+insulin | 97 | 60.5±9.0 | 55 | 34.1±5.5 | 14.7±7.4 | -0.41±0.19 | 8.5±1.1 | / | | +5±1 | |
|  |  |  | pioglitazone 45mg+insulin | 102 | 60.1±8.6 | 69 | 33.2±5.0 | 13.8±7.4 | -0.52±0.24 | 8.7±1.4 | / | | +4.9±1 | |
|  |  |  | PBO+insulin | 106 | 60.9±7.8 | 62 | 33.9±5.5 | 12.6±7.3 | 0.7±1.3 | 8.5±1.3 | / | | +0.5±1 | |
| Hollander, 2007[^97^](#_ENREF_97) | | 24 weeks | rosiglitazone 2mg/day+insulin | 193 | 52.7±9.6 | 57 | 32.8±7.4 | 12.5±8.3 | -0.6±1.1 | 8.9±1.1 | 97.2±22.3 | | +1.94±22.3 | |
|  |  |  | rosiglitazone 4mg/day+insulin | 189 | 52.6±10.1 | 48.1 | 33.7±7.1 | 13.0±7.3 | -0.8±1.2 | 9.0±1.2 | 96.8±20.8 | | +3.16±20.8 | |
|  |  |  | PBO+insulin | 186 | 53.8±10.2 | 46.2 | 33.0±6.5 | 12.6±8.6 | -0.3±1.3 | 9.1±1.3 | 95.1±20.9 | | +0.84±20.9 | |
| Hwang, 2008[^98^](#_ENREF_98) | | 12 months | Rosiglitazone 4mg/d+glibenclamide | 43 | 55.1±10.0 | 53.5 | 26.5±2.8 | / | -0.73±0.59 | / | 68.93±7.54 | | 2.87±2.29 | |
|  |  |  | PBO+glibenclamide | 46 | 53.4±9.7 | 45.7 | 26.6±2.5 | / | 0.26±1.27 | / | 68.02±8.83 | | 0.62±2,47 | |
| Iwamoto, 1996[^99^](#_ENREF_99) | | 12 weeks | Troglitazone 200mg bid+SU | 122 | 57.8±9.0 | 50.8 | 23.7±3.4 | / | -0.7±1.6 | 9.18±1.36 | 59.2±9.3 | | 0.6±1.3 | |
|  |  |  | PBO+SU | 126 | 58.7±8.0 | 42.9 | 23.3±3.1 | / | 0.2±1.7 | 8.98±1.45 | 57.7±9.6 | | -0.2±0.9 | |
| Kaku, 2009[^100^](#_ENREF_100) | | 28 weeks | Pioglitazone 30mg qd+metformin | 83 | 52±8.6 | 66.3 | 25.6±4.2 | 4.5±3.7 | -0.67±0.8 | 7.58±1.0 | / | | 1.68 | |
|  |  |  | PBO+metformin | 86 | 53±7.5 | 57 | 25.4±3.6 | 5.6±5.0 | 0.25±0.92 | 7.55±0.9 | / | | -0.47 | |
| Kawamori, 1998[^101^](#_ENREF_101) | | 12 weeks | pioglitazone 30mg Qd±SU | 21 | 57.6±8.5 | 66.7 | 23.0±1.8 | 12.5±9.1 | -0.8±1.4 | 8.4±1.4 | 61.1±6.6 | | +1.9±7.6 | |
|  |  |  | PBO±SU | 9 | 60.6±10.0 | 55.6 | 22.0±3.0 | 11.9±8.1 | -0.9±1.3 | 8.7±1.3 | 57.1±14.2 | | -1.1±14 | |
| Kelly, 1999[^102^](#_ENREF_102) | | 12 weeks | Troglitazone 200mg tid±SU | 11 | 58.0±8.6 | 72.7 | 28.7±3.9 | / | -0.84±0.7 | 7.51±1.38 | 78.9±11.2 | | 0.66±1.4 | |
|  |  |  | PBO±SU | 10 | 58.6±7.5 | 80 | 28.6±3.76 | / | 0.29±0.97 | 8.38±1.52 | 82.1±12.2 | | +0.25±0.9 | |
| Kipnes, 2001[^103^](#_ENREF_103) | | 16 weeks | pioglitazone 15mg Qd+SU | 184 | 56.5±9.8 | 59 | 31.4±5.0 | / | -0.8±0.2 | 10.0±0.2 | / | | / | |
|  |  |  | pioglitazone 30mg Qd+SU | 189 | 56.6±10.1 | 60 | 32.4±7.2 | / | -1.2±0.2 | 9.9±0.2 | / | | / | |
|  |  |  | PBO+SU | 187 | 56.9±8.9 | 58 | 32.0±4.9 | / | 0.1±0.2 | 9.9±0.2 | / | | / | |
| Marre, 2009[^104^](#_ENREF_104) | | 26 weeks | liraglutide 0.6mg+SU | 233 | 55.7±9.9 | 54 | 30.0±5.0 | 6.5 | -0.6±1 | 8.4±1.0 | 82.6±17.7 | | +0.7±17.7 | |
|  |  |  | liraglutide 1.2mg+SU | 228 | 57.7±9.0 | 45 | 29.8±5.1 | 6.7 | -1.08±1 | 8.5±1.1 | 80±17.1 | | +0.3±17.1 | |
|  |  |  | liraglutide 1.8mg+SU | 234 | 55.6±10.0 | 53 | 30.0±5.1 | 6.5 | -1.13±1 | 8.5±0.9 | 83±18.1 | | -0.2±18.1 | |
|  |  |  | PBO+SU | 114 | 54.7±10.0 | 47 | 30.3±5.4 | 6.5 | 0.23±1.0 | 8.4±1.0 | 81.9±17.1 | | -0.1±17.1 | |
|  |  |  | Rosiglitazone 4mg qd+SU | 232 | 56.0±9.8 | 47 | 29.4±4.8 | 6.6 | -0.44±1 | 8.4±1.0 | 80.6±17 | | +2.1±17 | |
| Mattoo, 2005[^105^](#_ENREF_105) | | 6 months | Pioglitazone 30mg qd+insulin | 142 | 58.8±7.4 | 43.7 | 32.5±4.8 | 13.6±6.8 | -0.69±0.09 | 8.85±0.11 | / | | 4.05±4.03 | |
|  |  |  | PBO+insulin | 147 | 58.9±6.9 | 42.9 | 31.8±5.0 | 13.4±6.1 | -0.13±0.1 | 8.79±0.10 | / | | 0.2±2.92 | |
| Mimura, 1994[^106^](#_ENREF_106) | | 3 months | CS 045 200mg bid | 8 | 53±2.7 | 50 | 22.4±0.7 | / | -2.5±0.4 | 9.3±0.4 | / | | / | |
|  |  |  | PBO | 6 | 58±2.1 | 50 | 21.3±1.4 | / | -0.4±0.3 | 9.7±0.3 | / | | / | |
| Negro, 2005[^107^](#_ENREF_107) | | 12 months | rosiglitazone 4mg bid+met | 19 | 60.3±6.4 | 52.6 | 28.3±1.7 | 7.1±2.4 | -1.1±0.7 | 8.4±0.6 | 84.1±4.6 | | +2.7±4.6 | |
|  |  |  | PBO+met | 19 | 59±8 | 63.2 | 28.7±1.9 | 6.6±2.9 | 0.2±0.5 | 8.1±0.5 | 83.6±4.4 | | 0.3±4.5 | |
| Osende, 2001[^108^](#_ENREF_108) | | 3 months | troglitazone 600mg Qd | 19 | 57.2±1.8 | 68.4 | 30.4±1.9 | / | -1.1±0.3 | 9.1±0.3 | / | | / | |
|  |  |  | PBO | 21 | 57.0±1.7 | 52.4 | 31.5±2.1 | / | -0.3±0.2 | 9.2±0.2 | / | | / | |
| Raskin, 2001[^109^](#_ENREF_109) | | 26 weeks | rosiglitazone 4mg/day+insulin | 106 | 57.7±10.2 | 56.6 | 32.1±4.8 | 12.7±7.3 | -0.6±1.1 | 9.1±1.3 | / | | / | |
|  |  |  | rosiglitazone 8mg/day+insulin | 103 | 57.1±10.0 | 54.4 | 32.3±4.9 | 12.5±8.0 | -1.2±1.1 | 9.0±1.3 | / | | / | |
|  |  |  | PBO+insulin | 104 | 55.6±10.3 | 55.8 | 32.7±6.2 | 11.7±6.2 | 0.1±1.0 | 8.9±1.1 | / | | / | |
| Reynolds, 2002[^110^](#_ENREF_110) | | 6 months | rosiglitazone 4mg/day+insulin | 8 | / | / | 36.4±1.8 | / | -1.1±0.4 | 8.0±0.3 | 109.6±9.2 | | -11.9±4.0 | |
|  |  |  | PBO+insulin | 10 | / | / | 36.3±1.8 | / | -1.3±0.5 | 9.8±0.5 | 106.4±6.4 | | -7.3±1.5 | |
| Rosenstock, 2008[^111^](#_ENREF_111) | | 26 weeks | rosiglitazone 4mg/day  +glimepide 3mg/day | 56 | 61±9 | 57 | 28.8±4.1 | 7.1±4.5 | -0.63±1.4 | 8.2±1.4 | 82.7±13.6 | | 0.9±13.6 | |
|  |  |  | rosiglitazone 8mg/day  +glimepide 3mg/day | 59 | 63±9 | 44 | 29.9±5.0 | 6.4±4.7 | -1.17±1.5 | 8.1±1.5 | 82.3±14.5 | | 1.7±14.5 | |
|  |  |  | PBO+glimepide 3mg/day | 57 | 65±9 | 60 | 29.1±4.5 | 6.6±3.9 | -0.08±1.3 | 7.9±1.3 | 83.1±14.2 | | -0.1±14.2 | |
| Scheen, 2009[^112^](#_ENREF_112) | | 34.5 months | Pioglitazone 45mg/d+metformin | 253 | 60.8±7.6 | 70 | 31.9±4.7 | 5.1±5.1 | -0.8±1.2 | 7.6±1.3 | / | | 3.9 | |
|  |  |  | PBO+metformin | 261 | 60.3±7.9 | 67 | 32.0±5.3 | 5.6±5.4 | -0.3±1.5 | 7.6±1.2 | / | | -1.3 | |
|  |  |  | Pioglitazone 45mg/d+SU | 508 | 63.2±7.7 | 68 | 29.7±4.6 | 7.3±6.0 | -0.9±1.3 | 7.8±1.3 | / | | 2.6 | |
|  |  |  | PBO+SU | 493 | 62.9±7.8 | 71 | 29.9±4.3 | 6.9±6.1 | -0.4±1.3 | 7.7±1.4 | / | | -1.3 | |
| Schwartz, 1998[^113^](#_ENREF_113) | | 26 weeks | troglitazone 200mg/day+insulin | 116 | 56±9 | 47 | 34.8±6.3 | 10±5 | -0.8±1.1 | 9.5±1.1 | 98.4±20.5 | | 2.1±20.5 | |
|  |  |  | troglitazone 600mg/day+insulin | 116 | 56±9 | 46 | 35.1±5.5 | 10±5 | -1.4±1.1 | 9.3±1.1 | 100.7±17.9 | | 3.8±17.9 | |
|  |  |  | PBO+insulin | 118 | 56±10 | 51 | 35.0±6.3 | 10±4 | -0.1±1.1 | 9.4±1.1 | 100．6±19.3 | | 1.4±19.3 | |
| Smith, 2005[^114^](#_ENREF_114) | | 24 weeks | piogitazone 45mg/day | 21 | 56.2±9.7 | 42.9 | 32.1±5.6 | / | -0.96±1.11 | 6.88±1.35 | 93.5±19.6 | | 3.88±3.1 | |
|  |  |  | PBO | 21 | 53.1±9.3 | 47.6 | 31.9±5.0 | / | -0.11±0.79 | 6.46±0.72 | 91.5±14.9 | | -0.79±3.36 | |
| Sridhar, 2013[^115^](#_ENREF_115) | | 24 weeks | pioglitazone 30mg/day  +glimepide+met | 25 | 47.9±5.8 | 100 | 25.3±2.7 | 2.2±1.7 | -0.4±0.4 | 6.8±0.4 | 70.4±11.4 | | +1.9±11.4 | |
|  |  |  | PBO+glimepide+met | 25 | 44.0±7.2 | 100 | 25.1±3.2 | 2.9±2.1 | -0.4±0.4 | 6.8±0.4 | 69.6±7.8 | | +0.3±7.8 | |
| Wolffenbuttel, 2000[^116^](#_ENREF_116) | | 26 weeks | rosiglitazone 1mg bid+SU | 199 | 61.0±9.4 | 62.8 | 28.0±3.9 | 7 | -0.59±1.2 | 9.20±1.19 | / | | / | |
|  |  |  | rosiglitazone 2mg bid+SU | 183 | 60.6±8.7 | 55.2 | 28.3±3.9 | 7 | -1.03±1.2 | 9.23±1.18 | / | | / | |
|  |  |  | PBO+SU | 192 | 61.9±9.1 | 57.3 | 28.1±4.1 | 8 | 0.25±1.3 | 9.21±1.30 | / | | / | |
| Yale, 2001[^117^](#_ENREF_117) | | 24 weeks | Troglitazone 400mg qd+metformin+SU | 101 | 58±0.9 | 55 | 30.1±0.5 | 11.9±0.8 | -1.4±0.2 | 9.6±0.1 | 85.2±1.6 | | +2.3±0.4 | |
|  |  |  | PBO+metformin+SU | 99 | 60±0.9 | 58 | 30.0±0.4 | 10.8±0.6 | 0.1±0.1 | 9.7±0.1 | 84.6±1.5 | | -0.1±0.4 | |
| Yang, 2002[^118^](#_ENREF_118) | | 6 months | Rosiglitazone 2mg bid+SU | 30 | 58.9±9.4 | 43.3 | 25.76±2.87 | / | -0.7±1 | 9.5±1.1 | 64.9±11.8 | | 3±2.4 | |
|  |  |  | PBO+SU | 34 | 57.8±8.9 | 38.2 | 25.84±3.50 | / | 0.4±1.3 | 9.7±1.4 | 65.3±11.2 | | -0.4±1.9 | |
| Zhu, 2003[^119^](#_ENREF_119) | | 24 weeks | rosiglitazone 2mg bid+SU | 215 | 59.0±7.5 | 41 | 24.8±3.4 | 7.2 | -1.4±0.15 | 9.9±1.6 | / | | / | |
|  |  |  | rosiglitazone 4mg bid+SU | 210 | 58.9±6.9 | 48 | 24.9±3.1 | 7.9 | -1.9±0.2 | 9.8±1.5 | / | | / | |
|  |  |  | PBO+SU | 105 | 58.8±7.7 | 46 | 25.1±2.8 | 7.6 | -0.4±0.25 | 9.8±1.3 | / | | / | |
| **Author , year** | | **Study duration** | **Treatment group** | **No. of patients** | **Age (years)** | **Men (%)** | **BMI (kg/m2)** | **DM duration**  **(years)** | **HbA1c change (%)** | **Baseline HbA1c (%)** | **Baseline Weight**  **(kg)** | | **Weight Change**  **(kg)** | |
| **DPP-IV inhibitor versus Placebo, monotherapy** | | | | | | | | | | |  | |  | |
| Dejager, 2007[^120^](#_ENREF_120) | | 24 weeks | Vildagliptin 50mg qd | 104 | 55.3±11.4 | 41.3 | 32.9±6.0 | 2.1±3.6 | -0.8±0.1 | 8.2±0.8 | / | | -1.8±0.4 | |
|  |  |  | Vildagliptin 50mg bid | 90 | 52.8±9.6 | 46.7 | 33.3±4.8 | 2.1±3.3 | -0.85±0.1 | 8.6±0.8 | / | | -0.3±0.4 | |
|  |  |  | Vildagliptin 100mg qd | 92 | 53.6±10.8 | 53.3 | 32.4±6.1 | 2.4±4.2 | -0.9±0.1 | 8.4±0.8 | / | | -0.8±0.4 | |
|  |  |  | Placebo | 94 | 52.2±11.2 | 47.9 | 32.6±5.6 | 1.6±2.5 | -0.3±0.1 | 8.4±0.8 | / | | -1.4±0.4 | |
| Frederich, 2012[^121^](#_ENREF_121) | | 24  weeks | Saxagliptin 2.5 mg q.A.M | 74 | 55.2±10.44 | 33.8 | 30.4±10.84 | 1.2±1.6 | -0.71±0.103 | 8.0±0.8 | 83.8±16.7 | | -0.3±0.35 | |
|  |  |  | Saxagliptin 5 mg q.A.M | 74 | 54.7±9.71 | 51.4 | 31.0±5.23 | 1.7±2.4 | -0.66±0.102 | 8.0±0.9 | 86.5±20.71 | | -0.9±0.31 | |
|  |  |  | Saxagliptin 2.5/5 mg q.A.M | 71 | 54.3±10.93 | 52.1 | 30.6±4.72 | 2.0±2.9 | -0.63±0.102 | 8.0±1.1 | 85.4±17.25 | | -0.9±0.41 | |
|  |  |  | Saxagliptin 5 mg q.P.M | 72 | 55.1±10.35 | 45.8 | 29.6±5.37 | 2.0±5.2 | -0.61±0.101 | 7.9±0.9 | 83.3±19.1 | | -0.4±0.36 | |
|  |  |  | Placebo | 74 | 55.6±10.32 | 47.3 | 31.1±4.54 | 1.7±2.8 | -0.26±0.103 | 7.8±1.0 | 85.4±14.4 | | -1.3±0.40 | |
| Haak, 2012[^122^](#_ENREF_122) | | 24 weeks | Linagliptin 5 mg | 142 | 56.2±10.8 | 56.3 | 29.0±4.7 | / | -0.5±0.1 | 8.7±1.0 | 79.1±17.3 | | / | |
|  |  |  | Metformin 500mg bid | 141 | 52.9±10.4 | 56.9 | 28.9±4.8 | / | -0.6±0.1 | 8.7±0.9 | 79.9±18.4 | | / | |
|  |  |  | Metformin 1000mg bid | 138 | 55.2±10.6 | 53.1 | 29.5±5.3 | / | -1.1±0.1 | 8.5±0.9 | 80±18.5 | | / | |
|  |  |  | Placebo | 72 | 55.7±11.0 | 50 | 28.6±5.2 | / | 0.1±0.1 | 8.7±1.0 | 76.8±15.0 | | / | |
| Hanefeld, 2007[^123^](#_ENREF_123) | | 12  Weeks | Sitagliptin 25mg qd | 111 | 55.1±9.6 | 51.4 | 31.9±4.8 | 3.6±3.4. | -0.28±0.9 | 7.7±0.9 | / | / | |  |
|  |  |  | Sitagliptin 50mg qd | 112 | 55.3±10.3 | 45.5 | 31.6±4.9 | 3.3±3.9 | -0.44±1.0 | 7.6±1.0 | / | / | |  |
|  |  |  | Sitagliptin 100mg qd | 110 | 56.0±7.9 | 55.5 | 31.6±5.8 | 3.6±3.9 | -0.44±0.9 | 7.8±0.9 | / | / | |  |
|  |  |  | Sitagliptin 50mg bid | 111 | 55.2±9.5 | 44.1 | 32.7±4.8 | 4.5±5.9 | -0.43±0.9 | 7.8±0.9 | / | / | |  |
|  |  |  | placebo | 111 | 55.9±9.3 | 63.1 | 31.4±5.1 | 3.3±3.4 | 0.12±0.9 | 7.6±0.9 | / | / | |  |
| Inagaki, 2014[^124^](#_ENREF_124) | | 12  weeks | Placebo | 55 | 61.6±9.79 | 66 | 24.8±3.4 | 6.0±4.0 | 0·35±0·63 | 8·15±0·95 | / | | / | |
|  |  |  | SYR-472 12.5mg | 54 | 60.6±10.24 | 61 | 25.8±3.4 | 7.8±7.3 | –0·37±0·49 | 8·18±0·89 | / | | / | |
|  |  |  | SYR-472 25mg | 52 | 58.5±10.49 | 65 | 25.8±4.4 | 6.0±6.1 | –0·32±0·47 | 7·99±0·77 | / | | / | |
|  |  |  | SYR-472 50mg | 51 | 61.0±10.18 | 53 | 25.5±3.9 | 5.8±4.7 | –0·42±0·46 | 8·07±0·86 | / | | / | |
|  |  |  | SYR-472 100mg | 55 | 57.8±10.38 | 51 | 25.1±4.0 | 6.1±5.2 | –0·55±0·50 | 8·41±0·97 | / | | / | |
|  |  |  | SYR-472 200mg | 54 | 60.5±11.26 | 67 | 25.2±3.2 | 7.0±6.1 | –0·54±0·44 | 7·84±0·76 | / | | / | |
| Iwamoto, 2010[^125^](#_ENREF_125) | | 12 weeks | Placebo | 73 | 60.2±8.0 | 68.5 | 24.1±3.2 | 6.4±5.5 | 0.28±0.12 | 7.74±0.93 | / | | -0.5±0.35 | |
|  |  |  | Sitagliptin 25mg qd | 80 | 59.9±7.9 | 63.8 | 25.0±3.6 | 4.7±4.3 | / | 7.49±0.82 | / | | 0.4±0.3 | |
|  |  |  | Sitagliptin 50mg qd | 72 | 60.2±9.4 | 65.3 | 24.5±3.6 | 5.6±6.4 | / | 7.57±0.84 | / | | 0.3±0.3 | |
|  |  |  | Sitagliptin 100mg qd | 70 | 58.3±9.5 | 51.4 | 24.2±2.8 | 5.4±5.4 | -0.69±0.12 | 7.56±0.80 | / | | 0.1±0.35 | |
|  |  |  | Sitagliptin 200mg qd | 68 | 60.6±7.7 | 58.8 | 24.4±3.0 | 5.1±4.9 | / | 7.65±0.82 | / | | 0.4±0.3 | |
| Kawamori, 2012[^126^](#_ENREF_126) | | 12 weeks | Linagliptin 5 mg | 159 | 60.3±9.4 | 69.8 | 24.6 ±4.0 | / | -0.24±0.06 | 8.07 ±0.66 | / | | / | |
|  |  |  | Linagliptin 10 mg | 160 | 61.3±10.0 | 70.0 | 25.0 ±3.8 | / | -0.25±0.06 | 7.98±0.68 | / | | / | |
|  |  |  | Placebo | 80 | 59.7±8.9 | 71.3 | 24.3±3.4 | / | 0.63±0.08 | 7.95±0.67 | / | | / | |
| Kikuchi, 2009[^127^](#_ENREF_127) | | 12 weeks | vildagliptin 10 mg | 71 | 58.9±8. | 73.2 | 24.4 ±2.7 | 4.5 ±4.2 | -0.8±0.1 | 7.4 ±0.8 | 64.2±10.4 | | -0.2±5 | |
|  |  |  | vildagliptin 25 mg | 72 | 57.8 ±8.5 | 63.9 | 24.3 ±2.5 | 4.7 ±4.5 | -1.0±0.1 | 7.4±0.9 | 63.6±9.7 | | 0.2±1.3 | |
|  |  |  | vildagliptin 50 mg | 76 | 58.8 ±8.6 | 67.1 | 24.3 ±2.8 | 4.7±4.3 | -1.2±0.1 | 7.4 ±0.8 | 62.7±9.3 | | 0.5±1.4 | |
|  |  |  | Placebo | 72 | 60.4±8.1 | 63.9 | 24.6 ±3.1 | 7.1 ±5.5 | 0.2±0.1 | 7.4 ±0.8 | 63.8±10.1 | | -0.5±1.1 | |
| Mohan, 2009[^128^](#_ENREF_128) | | 18 weeks | Sitagliptin 100mg qd | 352 | 50.9 ± 9.3 | 57 | 25.1± 3.4 | 2.1± 1.7 | -0.7±0.1 | 8.7 ± 1.0 | 66.8±10.2 | | 0.6±0.1 | |
|  |  |  | Placebo | 178 | 50.9 ± 9.3 | 60 | 24.9 ± 3.4 | 1.9 ±1.6 | 0.3±0.2 | 8.8± 1.1 | 66.6±11.4 | | 0±0.2 | |
| Nonaka, 2008[^129^](#_ENREF_129) | | 12 weeks | Sitagliptin 100mg qd | 75 | 55.6±8.6 | 60 | 25.2±3.5 | 4.0±4.1 | -0.65±0.15 | 7.5±0.9 | / | | -0.1±0.35 | |
|  |  |  | Placebo | 76 | 55.0±8.0 | 66 | 25.1±3.2 | 4.1±4.6 | 0.41±0.15 | 7.7±0.9 | / | | -0.7±0.3 | |
| Del Prato, 2011[^130^](#_ENREF_130) | | 24 weeks | Linagliptin 5 mg | 333 | 56.4±10.1 | 48.8 | 29.04±4.80 | / | -0.44±0.05 | 8.0±0.05 | 78.53±16.73 | | / | |
|  |  |  | Placebo | 163 | 54.4±10.3 | 47.3 | 29.08±4.84 | / | 0.25±0.07 | 8.0±0.07 | 79.21±15.95 | | / | |
| Pan, 2012[^131^](#_ENREF_131) | | 24 weeks | Saxagliptin 5mg qd | 284 | 51.2 ±10.0 | 56.3 | 25.9 ±3.4 | 0.8 ±1.4 | -0.84±0.1 | 8.1 ±0.8 | 69.2±11.4 | | -0.32±11.4 | |
|  |  |  | Placebo | 284 | 51.6 ±10.3 | 54.6 | 25.9 ±3.7 | 1.2 ±2.6 | -0.34±0.1 | 8.2 ±0.8 | 69.2±12.4 | | -1.14±12.4 | |
| Pi-Sunyer, 2007[^132^](#_ENREF_132) | | 24 weeks | Vildagliptin 50mg qd | 88 | 50.6±10.4 | 55.7 | 31.9±5.4 | 1.8±2.7 | -0.5±0.1 | 8.4±0.9 | 90.5±22.3 | | -0.4±0.4 | |
|  |  |  | Vildagliptin 50mg bid | 83 | 50.2±12.7 | 56.6 | 32.2±6.0 | 2.4±3.2 | -0.75±0.1 | 8.4±0.9 | 89.9±18.5 | | 0±0.4 | |
|  |  |  | Vildagliptin 100mg qd | 91 | 52.0±11.7 | 53.8 | 31.9±5.0 | 2.1±2.9 | -0.8±0.1 | 8.3±0.8 | 90.8±19.9 | | -0.4±0.3 | |
|  |  |  | placebo | 92 | 52.0±12.0 | 54.3 | 32.7±6.4 | 2.5±3.7 | 0.0±0.1 | 8.5±0.8 | 93±23.2 | | -1.4±0.4 | |
| Bergenstal, 2012[^133^](#_ENREF_133) | | 24 weeks | Taspoglutide 10mg | 182 | 55.3±9.5 | 56 | 32.7±5.2 | 6.1±4.8 | -1.23 | 7.95±0.93 | 93.6±20.4 | | -1.8±0.3 | |
|  |  |  | Taspoglutide 20mg | 187 | 56.8±8.8 | 52 | 32.3±5 | 5.7±4.7 | -1.3 | 7.97±0.86 | 91.8±18.0 | | -2.6±0.3 | |
|  |  |  | Sitagliptin 100mg | 177 | 55.5±9.9 | 59 | 32.4±5 | 6±5 | -0.89±0.85 | 7.94±0.85 | 92.5±19.7 | | -0.9±0.3 | |
|  |  |  | PBO | 90 | 56.1±10.1 | 52 | 32.5±5.5 | 5.5±3.9 | -0.1±0.83 | 8.03±0.83 | 91.1±19.0 | | -0.5±0.4 | |
| Ristic, 2005[^134^](#_ENREF_134) | | 12 weeks | Vildagliptin 25mg bid | 51 | 55.6±10.9 | 47.1 | 30.9±5.23 | 3.28±3.81 | -0.31±0.11 | 7.64±0.69 | 89.4±2.8 | | 0.06±0.33 | |
|  |  |  | Vildagliptin 25mg qd | 54 | 57.4±10.2 | 63 | 31.1±3.89 | 3.10±5.16 | -0.27±0.1 | 7.73±0.80 | 91.1±2 | | -0.55±0.32 | |
|  |  |  | Vildagliptin 50mg qd | 52 | 57.0±10.2 | 49.1 | 31.0±3.90 | 2.71±3.24 | -0.56±0.1 | 7.70±0.82 | 87.9±2.3 | | 0.04±0.33 | |
|  |  |  | Vildagliptin 100mg qd | 61 | 56.2±10.1 | 55.6 | 31.1±4.01 | 3.03±4.22 | -0.53±0.1 | 7.64±0.75 | 91.5±2.2 | | -0.07±0.31 | |
|  |  |  | Placebo | 55 | 54.6±10.6 | 56.9 | 31.6±4.41 | 2.28±2.99 | -0.13±0.1 | 7.76±0.83 | 92±2.1 | | -0.73±0.33 | |
| Roden, 2013[^135^](#_ENREF_135) | | 24  weeks | Placebo | 228 | 54.9±10.9 | 54 | 28.7±6.2 | / | 0.08±0.78 | 7.91±0.78 | 78.2±19.9 | | -0.33±0.33 | |
|  |  |  | Empagliflozin 10mg | 224 | 56.2±11.6 | 63 | 28.3±5.5 | / | -0.66±0.88 | 7.87±0.88 | 78.4±18.7 | | -2.26±0.35 | |
|  |  |  | Empagliflozin 25mg | 224 | 53.8±11.6 | 65 | 28.2±5.5 | / | -0.78±0.85 | 7.86±0.85 | 77.8±18.0 | | -2.48±0.35 | |
|  |  |  | Sitagliptin 100mg | 223 | 55.1±9.9 | 63 | 28.2±5.2 | / | -0.66±0.79 | 7.85±0.79 | 79.3±20.4 | | 0.18±0.34 | |
| Rosenstock, 2009[^136^](#_ENREF_136) | | 24 weeks | saxagliptin 2.5 mg | 102 | 53.27±10.06 | 56.9 | 31.90±4.82 | 3.1±3.5 | -0.43±0.1 | 7.9±0.9 | 92.1±18.39 | | -1.2±18.39 | |
|  |  |  | saxagliptin 5 mg | 106 | 53.91±11.57 | 50.9 | 32.24±4.5 | 2.5±3.3 | -0.46±0.1 | 8.0±1.1 | 90.87±18.15 | | -0.1±18.15 | |
|  |  |  | saxagliptin 10 mg | 98 | 52.72±11.27 | 45.9 | 31.71±4.71 | 2.3±3.1 | -0.54±0.1 | 7.8±0.9 | 89.3±17.67 | | -0.1±17.67 | |
|  |  |  | Placebo | 95 | 53.91±12.32 | 49.5 | 30.93±4.26 | 2.3±2.7 | 0.19±0.1 | 7.9±0.9 | 86.56±16.9 | | -1.4±16.9 | |
| Rosenstock, 2008[^137^](#_ENREF_137) | | 12 weeks | saxagliptin 2.5 mg | 55 | 52.5±10.53 | 40 | 30.8±3.73 | 1 | -0.72±0.12 | 7.7±0.97 | 86.6±14.17 | | -0.94±2.4 | |
|  |  |  | saxagliptin 5 mg | 47 | 53.7±10.14 | 53 | 30.8±4.21 | 0.8 | -0.9±0.14 | 7.9±1.09 | 89.8±15.92 | | -0.23±2.28 | |
|  |  |  | saxagliptin 10 mg | 63 | 54.5±8.61 | 63 | 31.0±4.03 | 0.7 | -0.81±0.11 | 8.0±1.14 | 92.4±17.78 | | -1.28±2.91 | |
|  |  |  | saxagliptin 20 mg | 54 | 53.6±8.59 | 70 | 29.7±3.63 | 1.7 | -0.74±0.12 | 7.9±0.99 | 88.9±14.63 | | -0.11±2.25 | |
|  |  |  | saxagliptin 40 mg | 52 | 54.1±11.12 | 58 | 29.8±4.29 | 1.3 | -0.80±0.12 | 7.8±1.00 | 86.8±19.55 | | 0.51±3.01 | |
|  |  |  | placebo | 67 | 55.2±9.8 | 63 | 31.1±4.46 | 1.8 | -0.27±0.11 | 8.0±0.88 | 93.1±19.21 | | -1.03±2.79 | |
| Scherbaum, 2008[^138^](#_ENREF_138) | | 52 weeks | Vildagliptin 50mg qd | 156 | 63.3±10.2 | 59.6 | 30.4±4.9 | 2.5±2.9 | -0.2±0.1 | 6.7±0.4 | / | | -0.5±0.3 | |
|  |  |  | placebo | 150 | 62.8±11.0 | 59.3 | 30.0±4.9 | 2.7±3.2 | 0.1±0.1 | 6.8±0.4 | / | | -0.2±0.3 | |
| Scott, 2007[^139^](#_ENREF_139) | | 12 weeks | Sitagliptin 5mg bid | 125 | 55.1±9.5 | 49.6 | 30.8±5.1 | 4.3±4.1 | -0.15±0.14 | 7.9±1.0 | / | | 0.1±1 | |
|  |  |  | Sitagliptin 12.5mg bid | 123 | 56.2±9.0 | 48 | 30.5±5.0 | 4.9±5.0 | -0.41±0.14 | 7.9±0.9 | / | | 0.1±1 | |
|  |  |  | Sitagliptin 25mg bid | 123 | 55.6±9.0 | 57.7 | 31.4±6.9 | 5.0±5.2 | -0.43±0.13 | 7.9±0.9 | / | | 0.3±1 | |
|  |  |  | Sitagliptin 50mg bid | 124 | 55.1±9.8 | 52.4 | 30.4±4.9 | 4.2±4.0 | -0.54±0.14 | 7.8±1.0 | / | | 0.4±1 | |
|  |  |  | placebo | 125 | 55.3±9.7 | 62.4 | 31.6±5.8 | 4.7±4.2 | 0.23±0.135 | 7.9±1.0 | / | | 0±1 | |
|  |  |  | Glipizide | 123 | 54.7±10.7 | 56.9 | 30.6±5.3 | 4.7±4.2 | -0.76±0.16 | 7.9±1 | / | | +0.9±1 | |
| Seino, 2011[^140^](#_ENREF_140) | | 12 weeks | Placebo | 75 | 59.1±10.47 | 74.7 | 24.39±3.69 | 6.83±6.07 | 0.06±0.456 | 7.85±0.89 | 64.57±12.01 | | -0.04±1.08 | |
|  |  |  | Alogliptin 6.25 mg | 79 | 58.1±10.58 | 70.9 | 24.75±4.85 | 6.63±6.41 | -0.51±0.677 | 7.85±0.94 | 65.5±14.47 | | 0.12±1.21 | |
|  |  |  | Alogliptin 12.5 mg | 84 | 58.7±9.54 | 70.2 | 24.86±4.04 | 5.94±5.32 | -0.70±0.572 | 7.99±0.88 | 67.21±13.41 | | 0.45±1.39 | |
|  |  |  | Alogliptin 25 mg | 80 | 59.5±11.16 | 78.8 | 24.70±4.15 | 6.98±6.99 | -0.76±0.547 | 7.88±0.99 | 66±13.34 | | 0.2±1.4 | |
|  |  |  | Alogliptin 50 mg | 79 | 58.8±9.23 | 69.6 | 24.29±3.02 | 6.78±5.95 | -0.82±0.474 | 8.01±0.97 | 63.8±10 | | 0.47±1.23 | |
|  |  |  | voglibose | 83 | 59.1±10.31 | 67.5 | 25.24±4.28 | 5.85±5.95 | -0.16±0.730 | 7.99±0.82 | 65.91±14.92 | | -0.46±1.62 | |
| **DPP-IV inhibitor versus Placebo, add-on therapy** | | | | | | | | | | |  | |  | |
| Ahrén, 2004[^141^](#_ENREF_141) | | 12 weeks | LAF237 50mg qd+MET | 56 | 57.9 ± 10.0 | 69.6 | 29.4± 3.6 | 5.6 ± 4.2 | -0.7±0.1 | 7.7 ± 0.6 | / | | -0.4±0.2 | |
|  |  |  | Placebo+MET | 51 | 55.7 ± 11.0 | 66.7 | 30.2 ± 3.6 | 5.5± 3.7 | 0.1±0.1 | 7.8 ± 0.7 | / | | -0.5±0.2 | |
| Barnett, 2012[^142^](#_ENREF_142) | | 24 weeks | Saxagliptin 5mg qd+insulin±met | 304 | 57.2±9.43 | 40 | 32.6±5.65 | 11.8±6.93 | -0.73±0.054 | 8.7±0.9 | 87.7±18.57 | | 0.39±0.151 | |
|  |  |  | Placebo+insulin±met | 151 | 57.3±9.27 | 45 | 31.8±4.76 | 12.2±7.37 | -0.32±0.074 | 8.6±0.86 | 86.2±16.54 | | 0.18±0.209 | |
| Barnett, 2013[^143^](#_ENREF_143) | | 24  weeks | Linagliptin 5mg qd | 162 | 74.9±4.4 | 71.6 | 29.6±4.7 | / | -0.61±0.06 | 7.8±0.8 | 86.3±16.4 | | / | |
|  |  |  | Placebo | 79 | 74.9±4.2 | 62.0 | 29.8±4.5 | / | 0.04±0.07 | 7.7±0.7 | 84.4±15.3 | | / | |
| Bosi, 2007[^144^](#_ENREF_144) | | 24 weeks | vildagliptin50mg+MET | 143 | 54.3±9.7 | 57.3 | 32.1±5.3 | 6.8±5.5 | -0.9±0.1 | 8.4±0.9 | / | | -0.4±0.3 | |
|  |  |  | Vildagliptin100mg+MET | 143 | 53.9±9.5 | 61.5 | 32.9±5.0 | 5.8±4.7 | -0.5±0.1 | 8.4±1.0 | / | | 0.2±0.3 | |
|  |  |  | Placebo+MET | 130 | 54.5±10.3 | 53.1 | 33.2±6.1 | 6.2±5.3 | 0.2±0.1 | 8.3±0.9 | / | | -1±0.3 | |
| Bosi, 2011[^145^](#_ENREF_145) | | 52  weeks | Met+Pio30mg+Alogliptin25mg | 404 | 54.3±9.86 | 52.0 | 31.5±5.25 | 7.5±5.24 | -0.70±0.86 | 8.2±0.86 | 88.2±18.9 | | 1.1±0.194 | |
|  |  |  | Met+Pio45mg | 399 | 55.9±9.94 | 51.1 | 31.6±5.18 | 6.9±4.61 | -0.29±0.83 | 8.1±0.83 | 88±19.28 | | 1.6±0.194 | |
| DeFronzo, 2012[^146^](#_ENREF_146) | | 26 weeks | placebo+MET | 129 | 55.2±9.9 | 47.3 | 30.6±4.8 | 6.0±5.0 | -0.9±0.05 | 8.5±0.6 | / | | / | |
|  |  |  | Alogliptin 12.5mg+MET | 128 | 53.1±9.6 | 52.3 | 31.0±5.1 | 6.2±5.6 | / | 8.6±0.7 | / | | / | |
|  |  |  | Alogliptin 25mg+MET | 129 | 53.7±9.3 | 38.8 | 31.5±5.7 | 5.6±4.9 | -1.4±0.05 | 8.6±0.7 | / | | / | |
|  |  |  | Placebo+PIO 15mg+MET | 130 | 54.1±9.5 | 46.9 | 31.3±5.0 | 5.7±4.8 | -0.9±0.05 | 8.5±0.7 | / | | / | |
|  |  |  | Alogliptin 12.5mg  +PIO 15mg+MET | 130 | 53.6±9.9 | 46.2 | 31.5±5.0 | 6.1±5.5 | / | 8.5±0.7 | / | | / | |
|  |  |  | Alogliptin 25mg  +PIO 15mg+MET | 130 | 54.9±9.2 | 46.9 | 30.8±4.7 | 6.9±5.5 | / | 8.5±0.7 | / | | / | |
|  |  |  | Placebo+PIO 30mg+MET | 129 | 56.1±9.4 | 48.8 | 31.4±5.4 | 7.6±7.1 | -0.9±0.05 | 8.5±0.7 | / | | / | |
|  |  |  | Alogliptin 12.5mg  +PIO 30mg+MET | 130 | 55.0±9.1 | 41.5 | 31.1±5.1 | 5.8±5.1 | / | 8.5±0.7 | / | | / | |
|  |  |  | Alogliptin 25mg  +PIO 30mg+MET | 130 | 54.4±9.7 | 42.3 | 31.9±5.6 | 6.6±6.0 | / | 8.5±0.7 | / | | / | |
|  |  |  | Placebo+PIO 45mg+MET | 129 | 54.5±9.7 | 41.1 | 30.7±4.7 | 5.7±4.2 | -0.9±0.05 | 8.5±0.7 | / | | / | |
|  |  |  | Alogliptin 12.5mg  +PIO 45mg+MET | 130 | 54.0±9.8 | 46.2 | 31.5±5.2 | 6.6±5.3 | / | 8.5±0.7 | / | | / | |
|  |  |  | Alogliptin 25mg  +PIO 45mg+MET | 130 | 54.2±8.9 | 40 | 30.6±4.8 | 6.2±5.0 | -1.6±0.1 | 8.5±0.7 | / | | / | |
| Derosa, 2012[^147^](#_ENREF_147) | | 12 months | Sitagliptin 100mg qd+MET | 91 | 55.9±8.8 | 46 | 28.1±1.2 | 5.8±2.6 | -1.4±0.7 | 8.1±0.8 | 78.4±6.6 | | -2.5±5 | |
|  |  |  | Placebo+MET | 87 | 54.8±7.9 | 51 | 28.9±2.0 | 5.4±2.3 | -0.7±0.6 | 8.0±0.7 | 78.6±6.7 | | -2.3±5.2 | |
| Derosa, 2012[^148^](#_ENREF_148) | | 12 months | Vildagliptin 50mg bid+ metformin | 84 | 54.2±8.3 | 50 | 27.9±1.5 | 6.1±3.7 | -1.2±0.6 | 8.1±0.6 | 76.9±5.8 | | -5.8±3.6 | |
|  |  |  | Placebo+ metformin | 83 | 52.4±7.1 | 51.8 | 27.8±1.4 | 6.3±3.9 | -0.8±0.7 | 8.2±0.7 | 78.5±6.4 | | -5.1±4.3 | |
| Dobs, 2013[^149^](#_ENREF_149) | | 18 weeks | Sitagliptin 100mg qd+MET+rosi | 170 | 54.4±8.8 | 56 | 30.1±6.2 | 9.3±5.9 | -1.0±0.15 | 8.8±1.0 | 82.5±19.7 | | 1.8±0.3 | |
|  |  |  | Placebo+MET+rosi | 92 | 54.8±9.5 | 60 | 30.8±5.6 | 9.4±6.8 | -0.3±0.2 | 8.7±1.0 | 86.8±20 | | 1.2±0.5 | |
| Fonseca, 2007[^150^](#_ENREF_150) | | 24 weeks | Vildagliptin 50mg bid+insulin | 144 | 59.6±10.3 | 47.9 | 33.3±5.2 | 14.4±8.6 | -0.5±0.1 | 8.4±1.0 | / | | 1.3±0.3 | |
|  |  |  | Placebo+insulin | 152 | 58.9±10.8 | 54.6 | 32.9±5.9 | 14.9±8.4 | -0.2±0.1 | 8.4±1.1 | / | | 0.6±0.3 | |
| Fonseca, 2013[^151^](#_ENREF_151) | | 26  weeks | Sitagliptin100mg+MET+PIO | 157 | 55.7±8.7 | 61.8 | 29.9±5.2 | 9.4±5.8 | -1.1±0.15 | 8.8±1.0 | 82.1±19.1 | | 1.3±2.9 | |
|  |  |  | Placebo+MET+PIO | 156 | 56.4±9.4 | 62.8 | 30.0±5.2 | 10.2±6.1 | -0.4±0.1 | 8.7±1.0 | 83.8±19.1 | | 1.1±2.4 | |
| Forst, 2010[^152^](#_ENREF_152) | | 12 weeks | Linagliptin 1mg+met | 65 | 59.1±8.4 | 55.4 | 32.2±4.3 | 6.9±5.9 | -0.15±0.7 | 8.2±0.7 | 92.5±16.9 | | -0.15±16.9 | |
|  |  |  | Linagliptin 5mg+met | 66 | 59.6±9.8 | 56.1 | 31.7±4.5 | 7.3±7.5 | -0.5±0.81 | 8.5±0.8 | 90.7±14.2 | | -0.57±14.2 | |
|  |  |  | Linagliptin 10mg+met | 66 | 61.8±8.8 | 53 | 31.7±4.5 | 8.2±6.8 | -0.42±0.7 | 8.4±0.7 | 89.9±16.3 | | -1.27±16.3 | |
|  |  |  | Glimepiride+met | 65 | 59.4±9.9 | 63.1 | 31.5±4.2 | 6.7±5.9 | -0.68±0.7 | 8.2±0.7 | 90.5±15 | | +0.73±15 | |
|  |  |  | Placebo+met | 71 | 60.1±8.1 | 62 | 32.2±4.2 | 6.2±5.1 | 0.25±0.7 | 8.4±0.7 | 93.1±16.8 | | -0.84±16.8 | |
| Garber, 2007[^153^](#_ENREF_153) | | 24 weeks | Vildagliptin 50mg+PIO | 124 | 54.0±8.2 | 54.8 | 32.6±5.0 | 4.7±4.3 | -0.8±0.1 | 8.6±1.0 | / | | 0.1±0.4 | |
|  |  |  | Vildagliptin 100mg+PIO | 136 | 54.0±9.2 | 44.9 | 32.2±5.8 | 4.6±4.8 | -1±0.1 | 8.7±1.2 | / | | 1.3±0.4 | |
|  |  |  | placebo+PIO | 138 | 54.8±10.6 | 50.7 | 32.3±5.8 | 4.8±4.6 | -0.3±0.1 | 8.7±1.2 | / | | 1.4±0.3 | |
| Garber, 2008[^154^](#_ENREF_154) | | 24 weeks | Vildagliptin 50mg+SU | 132 | 58.6±10.6 | 59.1 | 32.2±4.9 | 6.9±5.2 | -0.58±0.10 | 8.5±0.9 | / | | -0.1±0.3 | |
|  |  |  | Vildagliptin 100mg+SU | 132 | 58.2±11.1 | 59.8 | 30.8±5.3 | 6.7±5.3 | -0.63±0.09 | 8.6±1.0 | / | | 1.3±0.3 | |
|  |  |  | placebo+SU | 144 | 57.9±10.5 | 58.3 | 31.0±5.5 | 7.8±5.8 | 0.07±0.09 | 8.5±1.0 | / | | -0.4±0.3 | |
| Gomis, 2011[^155^](#_ENREF_155) | | 24 weeks | Linagliptin 5mg+pio | 259 | 57.7±9.6 | 58.7 | 28.7±4.8 | / | -1.06±0.06 | 8.6±0.05 | 78.3±15.6 | | 2.3±15.6 | |
|  |  |  | Placebo+pio | 130 | 57.1±10.1 | 65.4 | 29.7±4.8 | / | -0.56±0.09 | 8.58±0.08 | 82.7±15.8 | | 1.2±15.8 | |
| Goodman, 2009[^156^](#_ENREF_156) | | 24 weeks | Vildagliptin 100mg qd+MET | 248 | 54.9±10.8 | 52.8 | 31.4±4.7 | / | -0.595±0.11 | 8.5±1 | / | | 0.06±1 | |
|  |  |  | placebo+MET | 122 | 54.5±9.7 | 67.2 | 31.7±4.3 | / | 0.17±0.11 | 8.7±1.1 | / | | -0.69±1 | |
| Haak, 2012[^122^](#_ENREF_122) | | 24 weeks | Placebo+Metformin 500 mg bid | 144 | 52.9±10.4 | 56.9 | 28.9±4.8 | / | -0.6±0.1 | 8.7±0.9 | 79.9±18.4 | | / | |
|  |  |  | Placebo+Metformin 1000 mg bid | 147 | 55.2±10.6 | 53.1 | 29.5±5.3 | / | -1.1±0.1 | 8.5±0.9 | 80±18.5 | | / | |
|  |  |  | LINA2.5mg+MET500mg | 143 | 55.6±11.2 | 51 | 29.7±5.3 | / | -1.2±0.1 | 8.7±1.0 | 80.8±19 | | / | |
|  |  |  | LINA2.5mg+MET 1000mg bid | 143 | 56.4±10.7 | 53.8 | 28.6±4.8 | / | -1.6±0.1 | 8.7±1.0 | 76.7±16 | | / | |
| Hermansen, 2007[^157^](#_ENREF_157) | | 24 weeks | Sitagliptin 100mg qd+Glimepiride±met | 222 | 55.6±9.6 | 52.7 | 31.2±6.3 | 8.3±5.5 | -0.45±0.12 | 8.34±0.76 | 86.5±21.1 | | 0.8±0.4 | |
|  |  |  | Placebo+ Glimepiride±met | 219 | 56.5±9.6 | 53.4 | 30.7±6.3 | 9.3±6.8 | 0.28±0.12 | 8.34±0.74 | 85.9±21.8 | | -0.4±0.45 | |
| Hollander, 2009[^158^](#_ENREF_158) | | 24 weeks | Saxagliptin 2.5mg+TZD | 195 | 54.9±9.7 | 54.4 | 30.0±5.8 | 5.3±4.6 | -0.66±0.06 | 8.3±1.1 | 82.1±22 | | 1.3±22 | |
|  |  |  | Saxagliptin 5mg+TZD | 186 | 53.2±10.6 | 47.8 | 29.8±5.3 | 5.2±5.6 | -0.94±0.06 | 8.4±1.1 | 80.4±19.4 | | 1.4±19.4 | |
|  |  |  | placebo+TZD | 184 | 54.0±10.1 | 46.2 | 30.3±5.8 | 5.1±5.4 | -0.3±0.06 | 8.2±1.1 | 80.9±21.5 | | 0.9±21.5 | |
| Jadzinsky, 2009[^159^](#_ENREF_159) | | 24 weeks | Saxagliptin 5mg+met | 320 | 52.0±10.4 | 51.6 | 29.9±4.5 | 2.0±3.6 | -2.5±0.1 | 9.4±1.2 | 82.1±16.3 | | -1.8±16.3 | |
|  |  |  | Saxagliptin 10mg+met | 323 | 52.1±11.6 | 45.2 | 30.3±5.0 | 1.4±2.5 | -2.5±0.1 | 9.5±1.2 | 82.5±16.9 | | -1.4±16.9 | |
|  |  |  | placebo+met | 328 | 51.8±10.7 | 49.7 | 30.2±4.9 | 1.7±3.1 | -2±0.1 | 9.4±1.3 | 82.8±17.5 | | -1.6±17.5 | |
| Kaku, 2011[^160^](#_ENREF_160) | | 12 weeks | Placebo + pioglitazone | 115 | 60.1 ±9.7 | 76/39 | 26.4 ±4.4 | 6.7 ±5.3 | -0.19±0.55 | 7.92 ±0.85 | 69±14.4 | | -0.03±1.52 | |
|  |  |  | Alogliptin 12.5 mg + pioglitazone | 111 | 60.8 ±8.8 | 67/44 | 25.91 ±4.7 | 6.51 ±5.0 | -0.91±0.44 | 7.91 ±0.82 | 66.5±12.9 | | 0.48±1.26 | |
|  |  |  | Alogliptin 25 mg + pioglitazone | 113 | 59.3 ±10.7 | 70/43 | 26.07 ±3.7 | 6.80 ±5.7 | -0.97±0.52 | 7.89 ±0.73 | 68.07±13 | | 0.46±1.42 | |
| Kadowaki, 2014[^161^](#_ENREF_161) | | 12  weeks | Placebo+glimepiride | 98 | 60.3±7.8 | 67.3 | 24.6±3.6 | 8.3±6.2 | 0.3±0.1 | 8.4±0.8 | 65.9±11.8 | | -0.2±0.1 | |
|  |  |  | Teneligliptin 20mg qd+glimepiride | 96 | 58.4±8.6 | 64.6 | 24.9±3.6 | 9.3±6.7 | −0.7±0.1 | 8.4±0.8 | 66.2±12.1 | | 0.4±0.1 | |
| Kikuchi, 2010[^162^](#_ENREF_162) | | 12  weeks | Vildagliptin 50mg bid+glimepiride | 102 | 59.2±9.8 | 73.5 | 24.5±2.9 | 8.6±6.7 | -1.0±0.06 | 7.8±0.8 | 65±0.95 | | 0.97±0.12 | |
|  |  |  | placebo+glimepiride | 100 | 60.3±10.1 | 69 | 24.4±2.6 | 9.8±6.4 | -0.06±0.06 | 8±0.8 | 63.6±0.96 | | 0.06±0.12 | |
| Kothny, 2012[^163^](#_ENREF_163) | | 52 weeks | Vildagliptin 50mg qd | 122 | 67.1±9.0 | 57.4 | 30.3±5.2 | / | -0.6±0.1 | 7.9±1.0 | / | | / | |
|  |  |  | Placebo | 89 | 69.3±7.2 | 61.8 | 30.1±5.0 | / | -0.2±0.2 | 7.9±1.0 | / | | / | |
|  |  |  | vildagliptin | 94 | 63.7±9.1 | 52.1 | 30.8±5.8 | / | -0.8±0.2 | 7.7±1.0 | / | | / | |
|  |  |  | Placebo | 64 | 65.4±10.5 | 51.6 | 30.0±4.7 | / | -0.1±0.2 | 7.5±1.1 | / | | / | |
| Lavalle-González, 2013[^164^](#_ENREF_164) | | 26  weeks | Placebo+met | 183 | 55.3±9.8 | 51.4 | 31.1±6.1 | 6.8±5.3 | –0.17±0.06 | 8.0±0.9 | 86.6±22.4 | | -1.0±0.3 | |
|  |  |  | Sitagliptin100mg+met | 366 | 55.3±9.8 | 47 | 32.0±6.1 | 6.8±5.2 | -0.82±0.04 | 7.9±0.9 | 87.7±21.6 | | -1.2±0.2 | |
|  |  |  | Canagliflozin100mg+met | 368 | 55.5±9.4 | 47.3 | 32.4±6.4 | 6.7±5.4 | -0.79±0.04 | 7.9±0.9 | 88.8±22.2 | | -3.3±0.2 | |
|  |  |  | Canagliflozin300mg+met | 367 | 55.4±9.4 | 45 | 31.4±6.3 | 7.1±5.4 | -0.94±0.04 | 7.9±0.9 | 85.4±20.9 | | -3.7±0.2 | |
| Lewin, 2012[^165^](#_ENREF_165)) | | 18 weeks | Linagliptin 5mg+SU | 161 | 57.2±9.8 | 47.8 | 28.4±5.0 | / | -0.54±0.07 | 8.6±0.9 | / | | / | |
|  |  |  | Placebo+SU | 84 | 56.2±10.2 | 61.9 | 28.2±5.1 | / | -0.07±0.1 | 8.6±0.7 | / | | / | |
| Lukashevic, 2014[^166^](#_ENREF_166) | | 24  weeks | Vildagliptin 50mg bid+Met+Glimepiride | 158 | 55.3±10.2 | 50.6 | 27.9±4.6 | 7.1±6.2 | -1.01±0.9 | 8.7±0.9 | 73.1 | | 0.5±1 | |
|  |  |  | Placebo+Met+Glimepiride | 160 | 55.0±11.1 | 45% | 28.0±4.5 | 7.5±6.1 | -0.25±0.9 | 8.8±0.9 | 72.4 | | -0.1±1 | |
| Moses, 2014[^167^](#_ENREF_167) | | 24 weeks | Saxagliptin+MET+SU | 129 | 57.2±9.6 | 62.0 | 29.4±5.3 | / | -0.74 | / | 82.4±19.9 | | 0.2±2.2 | |
|  |  |  | PBO+MET+SU | 128 | 56.8±11.5 | 57.8 | 29.1±4.9 | / | -0.08 | / | 80.3±18.5 | | -0.6±2.1 | |
| Nauck, 2009[^168^](#_ENREF_168) | | 26 weeks | Placebo+MET | 104 | 56±11 | 48 | 32±6 | 6±5 | -0.1±0.1 | 8.0±0.9 | / | | 0±0.7 | |
|  |  |  | Alogliptin 12.5mg+ MET | 213 | 55±11 | 47.4 | 32±5 | 6±5 | -0.6±0.1 | 7.9±0.7 | / | | 0±0.7 | |
|  |  |  | Alogliptin 25mg+ MET | 210 | 54±11 | 54.3 | 32±5 | 6±4 | -0.6±0.1 | 7.9±0.8 | / | | -0.3±0.7 | |
| Nowicki, 2011[^169^](#_ENREF_169) | | 12 weeks | Saxagliptin2.5mg qd +Insulin/OADs | 85 | 66.8±8.3 | 37.6 | 31.2±6.1 | 15.1±7.5 | -0.86±0.112 | 8.5±1.2 | 83.6±15.7 | | -0.5±15.7 | |
|  |  |  | placebo+Insulin/OADs | 85 | 66.2±9.1 | 48.2 | 30.2±6.8 | 18.2±8.5 | -0.44±0.109 | 8.1±1.1 | 82.2±14.4 | | 0±14.4 | |
| Owens, 2011[^170^](#_ENREF_170) | | 24 weeks | Linagliptin 5 mg+MET+SU | 792 | 58.3±9.9 | 46.8 | 28.4±4.8 | / | -0.72±0.03 | 8.15±0.03 | 76.5±16.8 | | / | |
|  |  |  | Placebo+MET+SU | 263 | 57.6±9.7 | 48.3 | 28.2±4.5 | / | -0.1±0.05 | 8.14±0.05 | 76.8±16.8 | | / | |
| Pan, 2012[^171^](#_ENREF_171) | | 24 weeks | Vildagliptin 50mg bid+ MET | 146 | 54.2±9.62 | 50 | 26.01±3.26 | 4.92±4.8 | -1.05±0.08 | 8.09±0.85 | 71.58±11.93 | | / | |
|  |  |  | Vildagliptin 50 mg qd+ MET | 146 | 53.7±10.0 | 44.6 | 25.03±3.09 | 5.02±4.42 | -0.92±0.08 | 8.05±0.84 | 68.36±11.1 | | / | |
|  |  |  | Placebo+MET | 144 | 54.5±9.68 | 45.8 | 25.46±3.09 | 5.15±4.58 | -0.54±0.08 | 8.01±0.82 | 69.83±11.18 | | / | |
| Pratley, 2009[^172^](#_ENREF_172) | | 24 weeks | Alogliptin 12.5mg+pio | 197 | 55.5±9.4 | 55.3 | 32.3±5.7 | / | -0.66±0.9 | 8.1±0.9 | / | | / | |
|  |  |  | Alogliptin 25mg+pio | 199 | 55.4±10.2 | 62.8 | 33.1±5.4 | / | -0.8±0.8 | 8.0±0.8 | / | | / | |
|  |  |  | Placebo+pio | 97 | 55.2±10.8 | 54.6 | 33.2±6.2 | / | -0.19±0.8 | 8.0±0.8 | / | | / | |
| Pratley, 2009[^173^](#_ENREF_173) | | 26 weeks | Alogliptin 12.5mg+Glyburide | 203 | 56.5±11.1 | 54.7 | 30.2±4.8 | 7.8±6.1 | -0.39 | / | / | | 0.6±0.19 | |
|  |  |  | Alogliptin 25mg+Glyburide | 198 | 56.5±11.7 | 50.0 | 30.0±4.8 | 7.6±6.0 | -0.53 | / | / | | 0.68±0.19 | |
|  |  |  | Placebo+Glyburide | 99 | 57.1±10.0 | 51.5 | 30.0±5.3 | 7.7±5.3 | 0.01 | / | / | | -0.2±0.28 | |
| Raz, 2008[^174^](#_ENREF_174) | | 18 weeks | Sitagliptin 100mg qd+met | 96 | 53.6±9.5 | 51 | 30.1±4.4 | 8.4±6.5 | -1±0.2 | 9.3±0.9 | 81.5±16.8 | | -0.5±16.8 | |
|  |  |  | placebo+met | 94 | 56.1±9.5 | 41.5 | 30.4±5.3 | 7.3±5.3 | 0±0.05 | 9.1±0.8 | 81.2±19.4 | | -0.5±19.4 | |
| Rosenstock, 2006[^175^](#_ENREF_175) | | 24 weeks | Sitagliptin 100mg qd+PIO | 175 | 55.6±10.4 | 53.1 | 32.0±5.2 | 6.1±5.4 | -0.85±0.13 | 8.1±0.8 | 90.9±17 | | 1.8±0.65 | |
|  |  |  | Placebo+PIO | 178 | 56.9±11.1 | 57.9 | 31.0±5.0 | 6.1±5.7 | -0.15±0.12 | 8.0±0.8 | 86.4±17.4 | | 1.5±0.65 | |
| Ross, 2012[^176^](#_ENREF_176) | | 12 weeks | Linagliptin 2.5mg+ metformin | 214 | 58.7±9.9 | 61.9 | 29.8±5.2 | / | -0.46±0.05 | 7.96±0.78 | 82.2±17.2 | | -0.4±4.8 | |
|  |  |  | Linagliptin 5mg+ metformin | 221 | 58.4±10.6 | 54 | 29.6±5.0 | / | -0.52±0.05 | 7.98±0.72 | 80.6±17.5 | | -1±2.2 | |
|  |  |  | Placebo+ metformin | 43 | 59.9±10.7 | 47.7 | 28.7±5.5 | / | 0.28±0.11 | 7.92±0.74 | 77.7±19.4 | | -1.1±1.9 | |
| Scott, 2008[^177^](#_ENREF_177) | | 18 weeks | Sitagliptin 100mg qd+met | 94 | 55.2±9.8 | 55 | 30.3±4.7 | 4.9±3.5 | -0.73±0.13 | 7.8±1.0 | 83.1±17.1 | | -0.4±0.6 | |
|  |  |  | placebo+met | 92 | 55.3±9.3 | 59 | 30.0±4.5 | 5.4±3.7 | -0.22±0.14 | 7.7±0.9 | 84.6±16.5 | | -0.8±0.6 | |
|  |  |  | Rosiglitazone+met | 87 | 54.8±10.5 | 63 | 30.4±5.5 | 4.6±4 | -0.79±0.14 | 7.7±0.8 | 84.9±18.5 | | +1.5±0.45 | |
| Seino, 2011[^178^](#_ENREF_178) | | 12 weeks | Placebo+voglibose | 75 | 62.3 ±10.5 | 48/27 | 24.42 ±4.20 | 7.52 ±6.03 | 0.04±0.46 | 8.12±1.19 | 64.4±10.5 | | -0.23±1.26 | |
|  |  |  | Alogliptin12.5 mg+voglibose | 76 | 61.0 ±10.1 | 44/32 | 24.36 ±4.08 | 7.28 ±5.13 | / | 8.02 ±0.99 | 62.8±13.2 | | 0.19±1.05 | |
|  |  |  | Alogliptin25 mg+voglibose | 79 | 62.9 ±9.0 | 50/29 | 23.26 ±2.88 | 8.44 ±5.90 | -0.91±0.48 | 7.91±0.91 | 60.4±10.0 | | -0.19±0.39 | |
| Seino, 2012[^179^](#_ENREF_179) | | 24 weeks | Alogliptin 12.5 mg od +metformin | 92 | 53.4 ±8.80 | 65.2 | 25.63 ±4.10 | 6.34 ±5.39 | -0.54±0.56 | 7.89 ±0.82 | 69.47±12.46 | | / | |
|  |  |  | Alogliptin 25 mg od +metformin | 96 | 52.3 ±8.02 | 68.8 | 25.79 ±3.70 | 6.62 ±4.80 | -0.64±0.49 | 8.02 ±0.73 | 69.65±12.67 | | / | |
|  |  |  | Placebo + metformin | 100 | 52.1 ±8.05 | 72 | 26.14 ±4.58 | 6.04 ±4.36 | 0.21±0.64 | 8.00 ±0.86 | 69.89±14.23 | | / | |
| Strain, 2013[^180^](#_ENREF_180) | | 24  weeks | Vildagliptin 50mg bid | 139 | 75.1±4.3 | 52.5 | 29.1±3.8 | 12.2±7.9 | -0.9±0.8 | 7.9±0.8 | 79.9±1 | | -0.1±1 | |
|  |  |  | placebo | 139 | 74.4±4 | 38.1 | 30.5±4.8 | 10.6±6.9 | -0.3±0.7 | 7.9±0.7 | 80.7±1 | | -1±1 | |
| Taskinen, 2011[^181^](#_ENREF_181) | | 24 weeks | Placebo+MET | 177 | 56.6±10.9 | 57 | 30.05±5.01 | / | 0.15±0.06 | 8.02±0.88 | 83.3±16.6 | | / | |
|  |  |  | Linagliptin 5mg qd+MET | 523 | 56.5±10.1 | 53 | 29.85±4.84 | / | -0.49±0.04 | 8.09±0.86 | 82.2±17.2 | | / | |
| Vilsbøll, 2010[^182^](#_ENREF_182) | | 24 weeks | Sitagliptin 100mg qd+insulin | 322 | 58.3±9.1 | 49 | 31±5 | 13±7 | -0.6±0.1 | 8.7±0.9 | 86.5±18.6 | | 0.1±0.3 | |
|  |  |  | placebo+insulin | 319 | 57.2±9.3 | 53 | 31±5 | 12±6 | 0.0±0.1 | 8.6±0.9 | 87.3±17.9 | | 0.1±0.35 | |
| Yang, 2011[^183^](#_ENREF_183) | | 24 weeks | Saxagliptin 5mg qd+MET | 283 | 53.8 ±10.4 | 48.1 | 26.3 ±3.6 | 5.1 ±5.0 | -0.78±0.1 | 7.9 ±0.8 | 68.9±12.5 | | / | |
|  |  |  | Placebo+MET | 287 | 54.4 ±10.1 | 48.7 | 26.1 ±3.5 | 5.1 ±4.0 | -0.37±0.1 | 7.9±0.8 | 69±11.9 | | / | |
| Yang, 2012[^184^](#_ENREF_184) | | 24 weeks | Sitagliptin 100mg qd+MET | 197 | 54.1±9.0 | 47 | 25.3±3.1 | 6.4±4.4 | -1.0±0.15 | 8.5±0.9 | 67.9±10.7 | | 0±0.35 | |
|  |  |  | Placebo+MET | 198 | 55.1±9.8 | 55 | 25.3±3.6 | 7.3±4.6 | -0.1±0.15 | 8.5±0.9 | 68.9±13.3 | | -0.5±0.4 | |
| Yang, 2015[^185^](#_ENREF_185) | | 24  weeks | Vildagliptin50mg qd+glimepiride | 143 | 58.3±9.8 | 55.2 | 24.8±3.0 | 6.9±4.6 | −0.7±0.1 | 8.6±0.9 | 67.4±11.1 | | -0.3±11.1 | |
|  |  |  | Placebo+glimepiride | 136 | 58.7±9.3 | 58.1 | 25.0±2.8 | 6.9±4.1 | −0.2±0.1 | 8.7±1.0 | 68.8±11.7 | | -0.6±11.7 | |
| **Author , year** | | **Study duration** | **Treatment group** | **No. of patients** | **Age (years)** | **Men (%)** | **BMI (kg/m2)** | **DM duration**  **(years)** | **HbA1c change (%)** | **Baseline HbA1c (%)** | **Baseline Weight**  **(kg)** | | **Weight Change**  **(kg)** | |
| **SGLT2 versus Placebo, monotherapy** | | | | | | | | | | |  | |  | |
| Bailey, 2012[^186^](#_ENREF_186) | | 24 weeks | dapagliflozin 1.0mg/day | 72 | 53.7±9.04 | 52.8 | 32.53±5.68 | 1.6±2.55 | -0.68±0.23 | 7.8±0.98 | 88.2±18.49 | | -2.69±0.75 | |
|  |  |  | dapagliflozin 2.5mg/day | 74 | 53.5±10.61 | 45.9 | 31.13±5.47 | 1.5±2.19 | -0.72±0.23 | 8.1±1.07 | 84.3±18.18 | | -2.64±0.7 | |
|  |  |  | dapagliflozin 5.0mg/day | 68 | 51.3±11.51 | 47.1 | 30.97±5.68 | 1.4±3.24 | -0.82±0.25 | 7.9±1.03 | 85.4±19.43 | | -2.69±0.7 | |
|  |  |  | PBO | 68 | 53.5±11.08 | 54.4 | 32.47±4.91 | 1.1±1.95 | 0.02±0.2 | 7.8±1.12 | 90±17.98 | | -0.96±0.8 | |
| Ferrannini, 2010[^187^](#_ENREF_187) | | 24 weeks | dapagliflozin 2.5mg morning | 65 | 53.0±11.7 | 55.4 | 32.6±5.5 | 0.5 | -0.58±0.11 | 7.92±0.90 | 90.8±22.8 | | -3.3±0.5 | |
|  |  |  | dapagliflozin 5.0mg morning | 64 | 52.6±10.9 | 48.4 | 31.9±4.8 | 0.25 | -0.77±0.11 | 7.86±0.94 | 87.6±17.1 | | -2.8±0.5 | |
|  |  |  | dapagliflozin 10.0mg morning | 70 | 50.6±9.97 | 48.6 | 33.6±5.4 | 0.45 | -0.89±0.11 | 8.01±0.96 | 94.2±18.7 | | -3.2±0.5 | |
|  |  |  | dapagliflozin 2.5mg evening | 67 | 54.3±11.5 | 43.3 | 32.2±5.3 | 0.2 | -0.83±0.11 | 7.99±0.99 | 88.3±20.5 | | -3.8±0.5 | |
|  |  |  | dapagliflozin 5.0mg evening | 68 | 54.5±11.0 | 42.6 | 32.8±5.3 | 0.5 | -0.79±0.11 | 7.82±0.91 | 89.2±20.5 | | -3.6±0.5 | |
|  |  |  | dapagliflozin 10.0mg evening | 76 | 50.7±9.7 | 51.3 | 33.3±5.6 | 0.4 | -0.79±0.10 | 7.99±1.05 | 92.1±22.0 | | -3.1±0.4 | |
|  |  |  | dapagliflozin 5.0mg morning | 34 | 48.3±9.3 | 70.6 | 32.6±4.6 | 0.65 | -2.88±1.41 | 10.82±0.93 | 88.7±19.2 | | -2.1±3.4 | |
|  |  |  | dapagliflozin 10.0mg morning | 39 | 47.9±12.1 | 59 | 31.1±5.9 | 1.4 | -2.66±1.26 | 10.73±0.85 | 87.5±22.7 | | -1.9±3.5 | |
|  |  |  | PBO | 75 | 52.7±10.3 | 41.3 | 32.3±5.5 | 0.5 | -0.23±0.1 | 7.84±0.87 | 88.8±19 | | -2.2±0.4 | |
| Ferrannini, 2013[^188^](#_ENREF_188) | | 12 weeks | empagliflozin 5mg/day | 81 | 59 | 56.8 | 28.5 | / | -0.4±0.15 | 7.9±0.8 | 82.8±50 | | -1.81±1.03 | |
|  |  |  | empagliflozin 10mg/day | 81 | 58 | 49.4 | 28.1 | / | -0.5±0.18 | 8.0±0.8 | 76.8±50 | | -2.33±1 | |
|  |  |  | empagliflozin 25mg/day | 82 | 57 | 50 | 28.3 | / | -0.6±0.15 | 7.8±0.8 | 81.2±40 | | -2.03±1 | |
|  |  |  | PBO | 82 | 58 | 54.9 | 28.8 | / | 0.1±0.18 | 7.8±0.8 | 82.2±50 | | -0.75±1 | |
| Fonseca, 2013[^189^](#_ENREF_189) | | 12 weeks | ipraliflozin 12.5mg Qd | 70 | 53.9±9.6 | 55.7 | 31.0±5.9 | 4.08±3.24 | -0.22±0.8 | 7.95±0.78 | 86±22.3 | | -1.5±0.75 | |
|  |  |  | ipraliflozin 50mg Qd | 67 | 52.6±10.7 | 50.7 | 32.2±5.9 | 4.61±4.65 | -0.39±0.8 | 8.05±0.81 | 90.7±20.8 | | -1.66±7.5 | |
|  |  |  | ipraliflozin 150mg Qd | 68 | 54.2±10.3 | 42.6 | 30.9±6.3 | 5.11±6.46 | -0.47±0.6 | 7.83±0.65 | 83.3±21.6 | | -2.08±7.5 | |
|  |  |  | ipraliflozin 300mg Qd | 68 | 54.2±10.7 | 54.4 | 30.7±5.0 | 4.48±4.91 | -0.55±0.6 | 7.90±0.67 | 86.7±19.6 | | -2.67±7.5 | |
|  |  |  | metformin | 69 | 53.1±11.7 | 58 | 29.8±5.5 | 4.13±4.71 | -0.46±0.9 | 8.03±0.90 | 84.1±21.8 | | -0.88±7.5 | |
|  |  |  | PBO | 69 | 53.4±9.7 | 46.4 | 30.9±5.5 | 4.64±5.93 | 0.26±0.78 | 7.84±0.78 | 81.8±17.4 | | -1±7.5 | |
| Inagaki, 2013[^190^](#_ENREF_190) | | 12  weeks | Placebo | 75 | 57.7±11.0 | 72.0 | 26.41±4.34 | / | 0.11±0.77 | 7.99±0.77 | 72.56±15.36 | | -0.78±15.36 | |
|  |  |  | Canagliflozin 50mg | 82 | 57.4±10.8 | 61.0 | 25.11±4.13 | / | -0.61±0.78 | 8.13±0.78 | 65.77±13.56 | | -1.98±13.56 | |
|  |  |  | Canagliflozin 100mg | 74 | 57.7±10.5 | 70.3 | 25.61±4.64 | / | -0.80±0.86 | 8.05±0.86 | 68.61±14.86 | | -2.51±14.86 | |
|  |  |  | Canagliflozin 200mg | 76 | 57.0±10.7 | 64.5 | 25.51±4.30 | / | -0.79±0.88 | 8.11±0.88 | 68.97±14.5 | | -2.39±14.5 | |
|  |  |  | Canagliflozin 300mg | 75 | 57.1±10.1 | 73.3 | 25.89±3.68 | / | -0.88±0.81 | 8.17±0.81 | 71.3±12.19 | | -3.19±12.19 | |
| Ji, 2014[^191^](#_ENREF_191) | | 24  weeks | Placebo | 132 | 49.9±10.87 | 65.9 | 25.93±3.64 | 1.30±2.0 | -0.29±0.95 | 8.35±0.95 | 72.18±13.23 | | -0.27±0.45 | |
|  |  |  | Dapagliflozin 5mg | 128 | 53.0±11.07 | 65.6 | 25.17±3.29 | 1.15±2.3 | -1.04±0.74 | 8.14±0.74 | 68.89±11.43 | | -1.64±0.45 | |
|  |  |  | Dapagliflozin 10mg | 133 | 51.2±9.89 | 64.7 | 25.76±3.43 | 1.67±2.8 | -1.11±0.95 | 8.28±0.95 | 70.92±11.64 | | -2.25±0.15 | |
| List, 2009[^192^](#_ENREF_192) | | 12 weeks | dapagliflozin 2.5mg | 59 | 55±11 | 49 | 32±5 | / | -0.71±0.09 | 7.6±0.7 | 90±20 | | -2.7±0.75 | |
|  |  |  | dapagliflozin 5mg | 58 | 55±12 | 48 | 32±5 | / | -0.72±0.09 | 8.0±0.9 | 89±7 | | -2.5±0.75 | |
|  |  |  | dapagliflozin 10mg | 47 | 54±9 | 53 | 31±5 | / | -0.85±0.11 | 8.0±0.8 | 86±17 | | -2.7±0.85 | |
|  |  |  | dapagliflozin 20mg | 59 | 55±10 | 54 | 31±5 | / | -0.55±0.09 | 7.7±0.9 | 88±18 | | -3.4±0.75 | |
|  |  |  | dapagliflozin 50mg | 56 | 53±10 | 45 | 32±4 | / | -0.9±0.1 | 7.8±1.0 | 92±19 | | -3.4±0.75 | |
|  |  |  | PBO | 54 | 53±11 | 56 | 32±5 | / | -0.18±0.1 | 7.9±0.9 | 89±18 | | -1.2±0.75 | |
|  |  |  | metformin | 56 | 54±9 | 48 | 32±5 | / | -0.73±0.1 | 7.6±0.8 | 88±20 | | -1.7±0.75 | |
| Roden, 2013[^135^](#_ENREF_135) | | 24  weeks | Placebo | 228 | 54.9±10.9 | 54 | 28.7±6.2 | / | 0.08±0.78 | 7.91±0.78 | 78.2±19.9 | | -0.33±0.33 | |
|  |  |  | Empagliflozin 10mg | 224 | 56.2±11.6 | 63 | 28.3±5.5 | / | -0.66±0.88 | 7.87±0.88 | 78.4±18.7 | | -2.26±0.35 | |
|  |  |  | Empagliflozin 25mg | 224 | 53.8±11.6 | 65 | 28.2±5.5 | / | -0.78±0.85 | 7.86±0.85 | 77.8±18.0 | | -2.48±0.35 | |
|  |  |  | Sitagliptin 100mg | 223 | 55.1±9.9 | 63 | 28.2±5.2 | / | -0.66±0.79 | 7.85±0.79 | 79.3±20.4 | | 0.18±0.34 | |
| Stenlöf, 2013[^193^](#_ENREF_193) | | 26 weeks | canagliflozin 100mg/day | 195 | 55.1±10.8 | 41.5 | 31.3±6.6 | 4.5±4.4 | -0.77±1 | 8.1±1.0 | 85.8±21.4 | | -2.5±21.4 | |
|  |  |  | canagliflozin 300mg/day | 197 | 55.3±10.2 | 45.2 | 31.7±6.0 | 4.3±4.7 | -0.9±1 | 8.0±1.0 | 86.9±20.5 | | -3.4±20.5 | |
|  |  |  | PBO | 192 | 55.7±10.9 | 45.8 | 31.8±6.2 | 4.2±4.1 | 0.14±1.0 | 8.0±1.0 | 87.6±19.5 | | -0.5±19.5 | |
| **SGLT2 versus Placebo, add-on therapy** | | | | | | | | | | |  | |  | |
| Bailey, 2010[^194^](#_ENREF_194) | | 24  weeks | Placebo+met | 137 | 53.7±10.3 | 55 | 31·8±5·3 | 5.8±5.1 | –0.30±0.96 | 8.11±0.96 | 87.7±19.2 | | -0.9±0.5 | |
|  |  |  | Dapagliozin 2·5 mg+met | 137 | 55.0±9.3 | 51 | 31·6±4·8 | 6.0±6.2 | –0.67±0.90 | 7.99±0.90 | 84.9±17.8 | | -2.2±0.5 | |
|  |  |  | Dapagliflozin 5 mg+met | 137 | 54.3±9.4 | 50 | 31·4±5·0 | 6·4±5.8 | –0.70±0.96 | 8.17±0.96 | 84.7±16.3 | | -3±0.5 | |
|  |  |  | Dapagliflozin 10 mg+met | 135 | 52·7±9.9 | 57 | 31·2±5·1 | 6.1±5.4 | –0.84±0.82 | 7.92±0.82 | 86.3±11.5 | | -2.9±0.5 | |
| Barnett, 2014[^195^](#_ENREF_195) | | 52  weeks | Placebo(CKD,2) | 95 | 62·6±8.1 | 58.9 | 30.8±5.6 | / | 0.06±0.80 | 8.09±0.80 | 88.4±0.45 | | -0.44±0.63 | |
|  |  |  | Empagliflozin10mg(CKD,2) | 98 | 63.2±8.5 | 61.2 | 32.4±5.4 | / | –0.57±0.84 | 8.02±0.84 | 86.97±0.45 | | -2±0.6 | |
|  |  |  | Empagliflozin25mg(CKD,2) | 97 | 62.0±8.4 | 62.9 | 31.3±5.8 | / | –0.60±0.73 | 7.96±0.73 | 86.4±0.45 | | -2.6±0.6 | |
|  |  |  | Placebo(CKD,3) | 187 | 65.1±8.2 | 56.7 | 30.3±5.3 | / | 0.12±0.80 | 8.09±0.80 | 82.85±0.35 | | 0±0.4 | |
|  |  |  | Empagliflozin25mg(CKD,3) | 187 | 64.6±8.9 | 57.2 | 30.2±5.3 | / | –0.32±0.84 | 8.02±0.84 | 81.68±0.5 | | -1.17±0.4 | |
|  |  |  | Placebo(CKD,4) | 37 | 62.9±11.9 | 51.4 | 31.8±6.0 | / | –0.37±0.99 | 8.16±0.99 | 84.1±21 | | 0±3.6 | |
|  |  |  | Empagliflozin25mg(CKD,4) | 37 | 65.4±10.2 | 56.8 | 29.0±4.9 | / | 0.11±1.05 | 8.06±1.05 | 76.5±15.9 | | -1±3.3 | |
| Bolinder, 2012[^196^](#_ENREF_196) | | 24  weeks | Placebo+metformin | 91 | 60.8±6.9 | 59.2 | 31.7±3.9 | 5.5±5.3 | -0.1±0.53 | 7.16±0.53 | 90.9±13.7 | | -0.8±13.7 | |
|  |  |  | Dapagliflozin 10mg qd+metformin | 89 | 60.6±8.2 | 58 | 32.1±3.9 | 6.0±4.5 | -0.39±0.44 | 7.19±0.44 | 92.1±14.1 | | -2.88±14.1 | |
| Forst, 2014[^197^](#_ENREF_197) | | 26  weeks | Placebo+MET+PIO | 115 | 58.3±9.6 | 66.1 | 32.5±6.4 | 10.1±6.6 | -0.26±1.0 | 8.0±1.0 | 93.8±22.4 | | -0.2±22.4 | |
|  |  |  | Canagliflozin100mg+MET+PIO | 113 | 56.7±10.4 | 68.1 | 32.3±6.2 | 10.5±6.6 | -1.89±0.9 | 8.0±0.9 | 94.2±22.2 | | -2.6±22.2 | |
|  |  |  | Canagliflozin300mg+MET+PIO | 114 | 57.0±10.2 | 55.3 | 32.8±7.7 | 11.0±7.6 | -1.03±0.9 | 7.9±0.9 | 94.4±25.9 | | -3.7±25.9 | |
| Häring  2013[^198^](#_ENREF_198) | | 24  weeks | Empagliflozin 10mg+MET+SU | 225 | 57.0±9.2 | 50 | 28.3±5.4 | / | -0.82±0.05 | 8.07±0.81 | 77.1±18.3 | | -2.16±18.3 | |
|  |  |  | Empagliflozin 25mg+MET+SU | 216 | 57.4±9.3 | 53 | 28.3±5.5 | / | -0.77±0.05 | 8.10±0.83 | 77.5±18.8 | | -2.39±18.8 | |
|  |  |  | Placebo+MET+SU | 225 | 56.9±9.2 | 50 | 27.9±4.9 | / | -0.17±0.05 | 8.15±0.83 | 76.2±16.9 | | -0.39±16.9 | |
| Häring, 2014[^199^](#_ENREF_199) | | 24  weeks | Placebo+MET | 207 | 56.0±9.7 | 56 | 28.7±5.2 | / | -0.13±0.05 | 7.90±0.88 | 79.7±18.6 | | -0.45±18.6 | |
|  |  |  | Empagliflozin 10mg+MET | 217 | 55.5±9.9 | 58 | 29.1±5.5 | / | -0.7±0.05 | 7.94±0.79 | 81.6±18.5 | | -2.08±18.5 | |
|  |  |  | Empagliflozin 25mg+MET | 213 | 55.6±10.2 | 56 | 29.7±5.7 | / | -0.77±0.05 | 7.86±0.87 | 82.2±19.3 | | -2.46±19.3 | |
| Kovacs, 2014[^200^](#_ENREF_200) | | 24  weeks | Placebo+pio+met | 165 | 54.6 ±10.5 | 44.2 | 29.3±5.4 | / | −0.11±0.07 | 8.2±0.92 | 78.1±20.1 | | 0.34±0.21 | |
|  |  |  | Empagliflozin 10mg+pio+met | 165 | 54.7±9.9 | 50.3 | 29.2±5.6 | / | −0.59±0.07 | 8.1±0.89 | 78±19.1 | | -1.62±0.21 | |
|  |  |  | Empagliflozin 25mg+pio+met | 168 | 54.2±8.9 | 50.6 | 29.1±5.5 | / | −0.72±0.07 | 8.1±0.82 | 78.9±19.9 | | -1.47±0.21 | |
| Lavalle-González, 2013[^164^](#_ENREF_164) | | 26  weeks | Placebo+met | 183 | 55.3±9.8 | 51.4 | 31.1±6.1 | 6.8±5.3 | –0.17±0.06 | 8.0±0.9 | 86.6±22.4 | | -1.0±0.3 | |
|  |  |  | Sitagliptin100mg+met | 366 | 55.3±9.8 | 47 | 32.0±6.1 | 6.8±5.2 | -0.82±0.04 | 7.9±0.9 | 87.7±21.6 | | -1.2±0.2 | |
|  |  |  | Canagliflozin100mg+met | 368 | 55.5±9.4 | 47.3 | 32.4±6.4 | 6.7±5.4 | -0.79±0.04 | 7.9±0.9 | 88.8±22.2 | | -3.3±0.2 | |
|  |  |  | Canagliflozin300mg+met | 367 | 55.4±9.4 | 45 | 31.4±6.3 | 7.1±5.4 | -0.94±0.04 | 7.9±0.9 | 85.4±20.9 | | -3.7±0.2 | |
| Ljunggren, 2012[^201^](#_ENREF_201) | | 50 weeks | dapagliflozin 10mg/day+met | 89 | 60.6±8.2 | 55.1 | 32.1±3.9 | 6.0±4.5 | -0.38±0.11 | 7.19±0.44 | 92.1±14.1 | | -4.39±0.85 | |
|  |  |  | PBO+met | 91 | 60.8±6.9 | 56 | 31.7±3.9 | 5.5±5.3 | 0.02±0.1 | 7.16±0.53 | 90.9±13.7 | | -2.03±0.88 | |
| Rosenstock, 2012[^202^](#_ENREF_202) | | 12 weeks | canagliflozin 50mg Qd+met | 64 | 53.3±8.5 | 53 | 31.7±4.6 | 5.6±5.0 | -0.57±0.99 | 8.00±0.99 | 87.6±16.3 | | -2.3±16.3 | |
|  |  |  | canagliflozin 100mg Qd+met | 64 | 51.7±8.0 | 56 | 31.7±5.0 | 6.1±4.7 | -0.54±0.96 | 7.83±0.96 | 87.7±15.5 | | -2.6±15.5 | |
|  |  |  | canagliflozin 200mg Qd+met | 65 | 52.9±9.6 | 51 | 31.4±5.2 | 6.4±5.7 | -0.48±0.80 | 7.61±0.80 | 87.7±17 | | -2.7±17 | |
|  |  |  | canagliflozin 300mg Qd+met | 64 | 52.3±6.9 | 56 | 31.6±4.9 | 5.9±5.2 | -0.70±1.02 | 7.69±1.02 | 87.3±15.9 | | -3.4±15.9 | |
|  |  |  | canagliflozin 300mg bid+met | 64 | 55.2±7.1 | 44 | 31.8±5.2 | 5.8±4.6 | -0.22±0.89 | 7.73±0.89 | 86±19.1 | | -3.4±19.1 | |
|  |  |  | sitagliptin 100mg Qd+met | 65 | 51.7±8.1 | 58 | 31.6±5.0 | 5.6±4.7 | -0.74±0.95 | 7.64±0.95 | 87.2±18 | | -0.6±18 | |
|  |  |  | PBO+met | 65 | 53.3±7.8 | 48 | 30.6±4.6 | 6.4±5.0 | -0.22±0.9 | 7.75±0.83 | 85.9±19.5 | | -1.1±19.5 | |
| Rosenstock, 2014[^203^](#_ENREF_203) | | 52  weeks | Insulin+Placebo | 188 | 55.3±10.1 | 40 | 34.7±4.3 | / | -0.81±0.08 | 8.33±0.72 | 96.66±1.72 | | 0.44±0.36 | |
|  |  |  | Insulin+Empagliflozin 10mg | 186 | 56.7±8.7 | 52 | 34.7±3.8 | / | -1.18±0.08 | 8.39±0.74 | 94.57±1.47 | | -1.95±0.36 | |
|  |  |  | Insulin+Empagliflozin 25mg | 189 | 58.0±9.4 | 44 | 35.0±4.0 | / | -1.27±0.08 | 8.29±0.72 | 93.41±1.72 | | -2.04±0.36 | |
| Strojek, 2011[^204^](#_ENREF_204) | | 24 weeks | dapagliflozin 2.5mg/day  +glimepiride 4mg/day | 154 | 59.9±10.14 | 50 | / | 7.7±6.0 | -0.58±0.5 | 8.11±0.75 | 81.89±0.6 | | -1.18±0.6 | |
|  |  |  | dapagliflozin 5.0mg/day  +glimepiride 4mg/day | 142 | 60.2±9.73 | 50 | / | 7.4±5.7 | -0.63±0.5 | 8.12±0.78 | 81±0.6 | | -1.56±0.6 | |
|  |  |  | dapagliflozin 10mg/day  +glimepiride 4mg/day | 151 | 58.9±8.32 | 43.7 | / | 7.2±5.5 | -0.82±0.7 | 8.07±0.79 | 80.56±0.65 | | -2.26±0.65 | |
|  |  |  | PBO+glimepiride 4mg/day | 145 | 60.3±10.16 | 49 | / | 7.4±5.7 | -0.13±0.7 | 8.15±0.74 | 80.94±1 | | -0.72±1 | |
| Wilding, 2009[^205^](#_ENREF_205) | | 12 weeks | dapagliflozin 10mg+insulin | 24 | 55.7±9.2 | 54.2 | 35.5±3.6 | 11.8±5.8 | -0.61±0.25 | 8.4±0.7 | 103.4±10.2 | | -4.5±1 | |
|  |  |  | dapagliflozin 20mg+insulin | 24 | 56.1±10.6 | 54.2 | 36.2±4.6 | 11.3±5.6 | -0.69±0.25 | 8.5±0.9 | 101.2±15.3 | | -4.3±1 | |
|  |  |  | PBO+insulin | 23 | 58.4±6.5 | 69.6 | 34.8±4.6 | 13.8±7.3 | 0.09±0.3 | 8.4±0.9 | 101.8±16.5 | | -1.9±1 | |
| Wilding, 2012[^206^](#_ENREF_206) | | 48 weeks | dapagliflozin 2.5mg+insulin | 202 | 59.8±7.6 | 49.5 | 33.0±5.0 | 13.6±6.6 | -0.79±0.78 | 8.46±0.78 | 93±16.7 | | -0.96±0.75 | |
|  |  |  | dapagliflozin 5mg+insulin | 211 | 59.3±7.9 | 47.4 | 33.0±5.3 | 13.1±7.8 | -0.96±0.89 | 8.62±0.89 | 93.3±17.4 | | -1±0.75 | |
|  |  |  | dapagliflozin 10mg+insulin | 194 | 59.3±8.8 | 44.8 | 33.4±5.1 | 14.2±7.3 | -1.01±0.82 | 8.57±0.82 | 94.5±16.8 | | -1.61±0.75 | |
|  |  |  | PBO+insulin | 193 | 58.8±8.6 | 49.2 | 33.1±5.9 | 13.5±7.3 | -0.47±0.77 | 8.47±0.77 | 94.5±19.8 | | +0.82±0.75 | |
| Wilding, 2013[^207^](#_ENREF_207) | | 12 weeks | ipragliflozin 12.5mg/day+met | 69 | 56.6±8.5 | 47.8 | 31.9±4.9 | 6.8±6.4 | -0.53±0.17 | 7.78±0.64 | 89.5±13.6 | | -0.92±0.63 | |
|  |  |  | ipragliflozin 50mg/day+met | 68 | 58.6±7.6 | 47.1 | 31.1±4.9 | 6.0±5.3 | -0.65±0.18 | 7.76±0.66 | 86.7±13.7 | | -2.1±0.65 | |
|  |  |  | ipragliflozin 150mg/day+met | 67 | 58.1±8.2 | 56.7 | 31.8±5.2 | 5.7±4.8 | -0.72±0.18 | 7.73±0.69 | 89.3±17 | | -1.99±0.65 | |
|  |  |  | ipragliflozin 300mg/day+met | 72 | 56.6±8.9 | 50 | 31.8±4.6 | 5.5±4.8 | -0.79±0.17 | 7.87±0.82 | 89.3±15 | | -2.21±0.66 | |
|  |  |  | PBO+met | 66 | 57.3±8.6 | 54.5 | 32.0±4.8 | 5.7±3.2 | -0.31±0.18 | 7.68±0.60 | 89±14.5 | | -0.48±0.5 | |
| Yale, 2013[^208^](#_ENREF_208) | | 26 weeks | canagliflozin 100mg/day | 90 | 69.5±8.2 | 64.4 | 32.4±5.5 | 15.6±7.4 | -0.3±0.9 | 7.9±0.9 | 90.5±18.4 | | 0.2±0.65 | |
|  |  |  | canagliflozin 300mg/day | 89 | 67.9±8.2 | 53.9 | 33.4±6.5 | 17.0±7.8 | -0.39±0.9 | 8.0±0.8 | 90.2±18.1 | | -1.2±0.65 | |
|  |  |  | PBO | 90 | 68.2±8.4 | 63.3 | 33.1±6.5 | 16.4±10.1 | -0.03±0.9 | 8.0±0.9 | 92.8±17.4 | | -1.4±0.65 | |
| **Author , year** | | **Study duration** | **Treatment group** | **No. of patients** | **Age (years)** | **Men (%)** | **BMI (kg/m2)** | **DM duration**  **(years)** | **HbA1c change (%)** | **Baseline HbA1c (%)** | **Baseline Weight**  **(kg)** | | **Weight Change**  **(kg)** | |
| **GLP-1 versus Placebo, monotherapy** | | | | | | | | | | |  | |  | |
| Fonseca, 2012[^209^](#_ENREF_209) | | 12 weeks | lixisenatide 2-step dose increase | 120 | 53.5±9.7 | 52.5 | 32.3±6.7 | 1.4 | -0.94±0.9 | 7.98±0.9 | 89±22 | | -2±20 | |
|  |  |  | lixisenatide 1-step dose increase | 119 | 53.8±10.9 | 52.9 | 31.7±6.6 | 1.1 | -0.77±0.9 | 8.07±0.9 | 86.5±21 | |  |  |
|  |  |  | PBO | 122 | 54.1±11.0 | 49.2 | 31.8±6.7 | 1.4 | -0.27±0.9 | 8.07±0.9 | 86.1±22 | |  |  |
| Madsbad, 2004[^210^](#_ENREF_210) | | 12 weeks | liraglutide 0.045mg Qd | 26 | 53±9.0 | 84.6 | 30.2±5.4 | 4.1±3.7 | 0.25±0.8 | 7.4±0.8 | / | | -0.03±0.96 | |
|  |  |  | liraglutide 0.225mg Qd | 24 | 58±7.5 | 62.5 | 32.0±5.3 | 4.4±4.0 | -0.34±0.8 | 7.9±0.8 | / | | -0.74±0.15 | |
|  |  |  | liraglutide 0.45mg Qd | 27 | 57±11.3 | 66.7 | 30.1±5.0 | 4.5±4.6 | -0.3±1 | 7.7±1.0 | / | | -1.2±0.02 | |
|  |  |  | liraglutide 0.60mg Qd | 30 | 57±7.7 | 66.7 | 30.4±4.8 | 4.6±4.6 | -0.7±1 | 7.4±1.2 | / | | 0.27±0.58 | |
|  |  |  | liraglutide 0.75mg Qd | 28 | 58±9.7 | 57.1 | 31.9±4.3 | 6.1±7.9 | -0.75±0.9 | 7.4±0.9 | / | | -0.39±0.44 | |
|  |  |  | PBO | 29 | 57±9.4 | 69 | 30.3±4.2 | 3.8±3.4 | -0.1±1.0 | 7.8±0.9 | / | | 0 | |
|  |  |  | glimepiride | 26 | 57±9.2 | 61.5 | 30.2±4.6 | 3.4±2.9 | -0.74±1.2 | 7.4±1.2 | / | | 0.94±0.02 | |
| Moretto, 2008[^211^](#_ENREF_211) | | 24 weeks | exenatide 5ug/day | 77 | 54±10 | 52 | 32±5 | 2±3 | -0.7±0.1 | 7.9±1.0 | 85±15 | | -2.8±0.3 | |
|  |  |  | exenatide 10ug/day | 78 | 55±10 | 62 | 31±5 | 2±3 | -0.9±0.1 | 7.8±1.0 | 86±16 | | -3.1±0.3 | |
|  |  |  | PBO | 77 | 53±9 | 55 | 32±5 | 1±2 | -0.2±0.1 | 7.8±0.9 | 86±16 | | -1.4±0.3 | |
| Bergenstal, 2012[^133^](#_ENREF_133) | | 24 weeks | Taspoglutide 10mg | 182 | 55.3±9.5 | 56 | 32.7±5.2 | 6.1±4.8 | -1.23 | 7.95±0.93 | 93.6±20.4 | | -1.8±0.3 | |
|  |  |  | Taspoglutide 20mg | 187 | 56.8±8.8 | 52 | 32.3±5 | 5.7±4.7 | -1.3 | 7.97±0.86 | 91.8±18.0 | | -2.6±0.3 | |
|  |  |  | Sitagliptin 100mg | 177 | 55.5±9.9 | 59 | 32.4±5 | 6±5 | -0.89 | 7.94±0.85 | 92.5±19.7 | | -0.9±0.3 | |
|  |  |  | PBO | 90 | 56.1±10.1 | 52 | 32.5±5.5 | 5.5±3.9 | -0.1 | 8.03±0.83 | 91.1±19.0 | | -0.5±0.4 | |
| Seino, 2008[^212^](#_ENREF_212) | | 14 weeks | liraglutide 0.1mg/day | 45 | 56.5±8.4 | 68.9 | 24.26±2.77 | 7.15±5.14 | -0.72±0.96 | 8.50±0.84 | 64.82±10.29 | | -0.05±0.68 | |
|  |  |  | liraglutide 0.3mg/day | 46 | 56.8±8.8 | 69.6 | 23.93±3.09 | 6.78±4.69 | -1.07±1.1 | 8.24±0.92 | 62.42±11.18 | | 0.13±0.68 | |
|  |  |  | liraglutide 0.6mg/day | 45 | 60.0±7.0 | 62.2 | 23.74±2.78 | 8.87±6.77 | -1.5±0.9 | 8.21±0.83 | 61.97±9.40 | | -0.1±0.68 | |
|  |  |  | liraglutide 0.9mg/day | 44 | 55.5±7.6 | 70.5 | 23.59±3.04 | 7.62±4.92 | -1.67±1.0 | 8.12±0.98 | 62.36±10.65 | | -0.48±0.68 | |
|  |  |  | PBO | 46 | 57.5±8.7 | 63 | 23.77±2.63 | 7.48±5.65 | 0.09±1.2 | 8.43±1.02 | 62.78±10.88 | | -0.95±0.68 | |
| Vilsbøll, 2007[^213^](#_ENREF_213) | | 14 weeks | liraglutide 0.65mg/day | 40 | 56.5±9.3 | 67.5 | 28.9±3.9 | 6 | -0.98±0.9 | 8.1±0.6 | / | | / | |
|  |  |  | liraglutide 1.25mg/day | 42 | 53.8±10.7 | 54.8 | 31.2±4.7 | 7 | -1.40±0.9 | 8.3±0.8 | / | | / | |
|  |  |  | liraglutide 1.9mg/day | 41 | 55.4±11.4 | 73.2 | 29.9±4.2 | 4 | -1.45±0.9 | 8.5±0.9 | / | | -2.99±1 | |
|  |  |  | PBO | 40 | 57.7±8.2 | 47.5 | 30.4±4.0 | 5 | +0.29±0.9 | 8.2±0.7 | / | | -1.78±1 | |
| Vilsbøll, 2008[^214^](#_ENREF_214) | | 14 weeks | liraglutide 0.65mg/day | 8 | 61.1±7.6 | 100 | 26.6±2.8 | 7.1±2.5 | -1.0±0.8 | 8.7±0.7 | 83.1±13.1 | | -1.3±2 | |
|  |  |  | liraglutide 1.25mg/day | 10 | 56.9±10.1 | 100 | 29.1±3.2 | 7.9±2.7 | -1.3±0.7 | 8.4±0.6 | 94.3±10.4 | | -2.4±2.4 | |
|  |  |  | liraglutide 1.9mg/day | 11 | 58.6±10.3 | 86 | 31.4±3.3 | 4.6±3.2 | -1.5±0.7 | 8.2±0.6 | 93.1±19.7 | | -2.8±2.7 | |
|  |  |  | PBO | 10 | 55.4±6.7 | 80 | 30.3±4.3 | 1.8±0.8 | 1.5±1.8 | 8.1±0.3 | 84.5±19.5 | | -4±4.8 | |
| **GLP-1 versus Placebo, add-on therapy** | | | | | | | | | | |  | |  | |
| Apovian, 2010[^215^](#_ENREF_215) | | 24 weeks | Exenatide 10ug bid+metformin | 52 | 53.4±10.7 | 29 | 32.9±3.5 | 4.3±3.2 | -0.9±0.8 | 7.5±0.8 | 91.4±13.2 | | -7.3±13.2 | |
|  |  |  | metformin+PBO | 51 | 55.0±7.9 | 39 | 33.6±4.6 | 3.9±3.2 | -0.4±0.5 | 7.2±0.5 | 94.9±14.3 | | -4.3±14.3 | |
|  |  |  | metformin+SU+exenatide10ug bid | 33 | 55.9±9.3 | 48 | 34.0±3.7 | 8.5±7.5 | -1.4±0.8 | 8.0±0.8 | 98.6±20.1 | | -5.2±20.1 | |
|  |  |  | metformin+SU+PBO | 36 | 55.1±9.9 | 31 | 34.3±4.0 | 7.6±6.9 | -0.9±0.9 | 7.9±0.9 | 96.5±17 | | -3.6±17 | |
|  |  |  | Exenatide 10ug bid+SU | 11 | 55.6±8.7 | 45 | 36.2±4.2 | 4.0±3.5 | -1.5±1.1 | 7.9±1.1 | 100.6±16.5 | | -4.2±16.5 | |
|  |  |  | SU+PBO | 11 | 55.3±11.3 | 54 | 33.8±4.3 | 4.3±2.8 | -1.3±1.1 | 7.7±1.1 | 100.8±17.4 | | -4.6±17.4 | |
| Buse, 2004[^216^](#_ENREF_216) | | 30 weeks | exenatide 5ug bid+SU | 125 | 55±10 | 59.2 | 33±6 | 6.3±5.2 | -0.46±0.12 | 8.5±1.1 | 95±22 | | -0.9±0.3 | |
|  |  |  | exenatide 10ug bid+SU | 129 | 56±11 | 57.4 | 33±6 | 6.6±6.6 | -0.86±0.11 | 8.6±1.2 | 95±18 | | -1.6±0.3 | |
|  |  |  | PBO+SU | 123 | 55±11 | 62.6 | 34±5 | 5.7±4.7 | 0.12±0.09 | 8.7±1.2 | 99±18 | | -0.6±0.3 | |
| Defronzo, 2005[^217^](#_ENREF_217) | | 30 weeks | exenatide 5ug bid+metformin | 110 | 55±11 | 51.8 | 34±6 | 6.2±5.9 | -0.4±0.11 | 8.3±1.1 | 100±22 | | -1.6±0.4 | |
|  |  |  | exenatide 10ug bid+metformin | 113 | 52±11 | 60.2 | 34±6 | 4.9±4.7 | -0.8±0.1 | 8.2±1.0 | 101±20 | | -2.8±0.5 | |
|  |  |  | PBO+metformin | 113 | 54±9 | 59.3 | 34±6 | 6.6±6.1 | 0.08±0.1 | 8.2±1.0 | 100±19 | | -0.3±0.3 | |
| Gao, 2009[^218^](#_ENREF_218) | | 12 weeks | Exenatide 10ug bid+met or met/SU | 234 | 55±9 | 48 | 26.4±3.2 | 8±6 | -1.2±0.1 | 8.3±1.0 | 69.6±11.2 | | -1.2±0.3 | |
|  |  |  | PBO+met or met/SU | 232 | 54±9 | 41 | 26.1±3.4 | 8±5 | -0.4±0.1 | 8.3±1.0 | 67.9±11.1 | | -0.1±0.25 | |
| Kadowaki, 2009[^219^](#_ENREF_219) | | 12 weeks | exenatide 2.5ug/day | 37 | 62.2±7.8 | 70.3 | 24.2±3.3 | 14.8±10.9 | -0.9±0.1 | 8.0±0.8 | 64.9±11.6 | | 0.08±0.2 | |
|  |  |  | exenatide 5ug/day | 37 | 60.7±9.8 | 67.6 | 25.0±3.4 | 11.3±6.4 | -1.2±0.1 | 7.9±0.8 | 65.6±9.8 | | -0.2±0.3 | |
|  |  |  | exenatide 10ug/day | 37 | 57.8±10.4 | 62.6 | 26.1±5.3 | 9.6±6.0 | -1.2±0.1 | 7.9±0.9 | 70.3±15.9 | | -1.3±0.3 | |
|  |  |  | PBO | 40 | 60.5±10.2 | 75 | 25.8±4.6 | 11.9±6.0 | 0.02±0.1 | 8.1±0.7 | 71.1±14 | | -0.7±0.2 | |
| Kaku, 2010[^220^](#_ENREF_220) | | 24 weeks | liraglutide 0.6mg/day+SU | 88 | 59.1±10.3 | 60 | 25.3±3.6 | 9.3±5.8 | -1.46±0.95 | 8.60±0.91 | 66.1±12.1 | | -1.12±0.55 | |
|  |  |  | liraglutide 0.9mg/day+SU | 88 | 61.3±11.0 | 67 | 24.4±3.4 | 11.6±7.7 | -1.56±0.84 | 8.21±0.78 | 64.5±12.0 | | -0.48±0.55 | |
|  |  |  | PBO+SU | 88 | 58.6±9.7 | 65 | 24.9±4.0 | 10.1±7.3 | -0.4±0.93 | 8.45±0.99 | 66.7±13.5 | | -0.37±0.55 | |
| Kendall, 2005[^221^](#_ENREF_221) | | 30 weeks | exenatide 5ug bid+met+SU | 245 | 55±9 | 59.2 | 33±6 | 8.7±5.9 | -0.7±0.1 | 8.5±1.0 | 97±19 | | -1.6±0.2 | |
|  |  |  | exenatide 10ug bid+met+SU | 241 | 55±10 | 59.3 | 34±6 | 8.7±6.4 | -0.6±0.1 | 8.5±1.1 | 98±21 | | -1.6±0.2 | |
|  |  |  | PBO+met+SU | 247 | 56±10 | 55.9 | 34±5 | 9.4±6.2 | 0.2±0.1 | 8.5±1.0 | 99±19 | | -0.9±0.2 | |
| Kim, 2007[^222^](#_ENREF_222) | | 15 weeks | exenatide LAR 0.8mg/day | 15 | 51±12 | 75 | 35±6 | 5±3 | -1.4±0.3 | 8.6±1.1 | 107±24 | | 0±1 | |
|  |  |  | exenatide LAR 2.0mg/day | 15 | 51±11 | 67 | 36±6 | 4±5 | -1.7±0.3 | 8.3±1.1 | 110±17 | | -3.8±1.4 | |
|  |  |  | PBO | 15 | 55±9 | 36 | 36±6 | 4±4 | 0.4±0.3 | 8.6±1.4 | 101±20 | | 0±1 | |
| Marre, 2009[^104^](#_ENREF_104) | | 26 weeks | liraglutide 0.6mg/day  +glimepiride 2-4mg/day | 233 | 55.7±9.9 | 54 | 30.0±5.0 | 6.5 | -0.6±1.0 | 8.4±1.0 | / | | / | |
|  |  |  | liraglutide 1.2mg/day  +glimepiride 2-4mg/day | 228 | 57.7±9.0 | 45 | 29.8±5.1 | 6.7 | -1.1±1.5 | 8.5±1.1 | / | | / | |
|  |  |  | liraglutide 1.8mg/day  +glimepiride 2-4mg/day | 234 | 55.6±10.0 | 53 | 30.0±5.1 | 6.5 | -1.1±1.1 | 8.5±0.9 | / | | / | |
|  |  |  | PBO+glimepiride 2-4mg/day | 114 | 54.7±10.0 | 47 | 30.3±5.4 | 6.5 | 0.2±1.0 | 8.4±1.0 | / | | / | |
|  |  |  | liraglutide 0.6mg/day  +rosiglitazone 4mg/day | 232 | 56.0±9.8 | 47 | 29.4±4.8 | 6.6 | -0.4±0.8 | 8.4±1.0 | / | | / | |
| Nauck, 2009[^8^](#_ENREF_8) | | 26 weeks | liraglutide 0.6mg/day+met | 242 | 56±11 | 62 | 30.5±4.8 | 7±5 | -0.7±0.1 | 8.4±0.9 | / | | / | |
|  |  |  | liraglutide 1.2mg/day+met | 240 | 57±9 | 54 | 31.1±4.8 | 7±5 | -1.0±0.1 | 8.3±1.0 | / | | / | |
|  |  |  | liraglutide 1.8mg/day+met | 242 | 57±9 | 59 | 30.9±4.6 | 8±5 | -1±0.1 | 8.4±1.0 | / | | / | |
|  |  |  | glimepiride 4mg/day+met | 242 | 57±9 | 57 | 31.2±4.6 | 8±5 | -1±0.1 | 8.4±1.0 | / | | / | |
|  |  |  | PBO+met | 121 | 56±9 | 60 | 31.6±4.4 | 8±6 | 0.1±0.1 | 8.4±1.1 | / | | / | |
| Ratner, 2010[^223^](#_ENREF_223) | | 13 weeks | lixisenatide 5ug Qd+met | 55 | 56.8±7.8 | 47.3 | 30.7±4.6 | 7.2±4.9 | -0.47±0.7 | 7.58±0.7 | 84.6±16 | | -2±0.4 | |
|  |  |  | lixisenatide 10ug Qd+met | 52 | 55.4±9.2 | 59.6 | 31.9±4.0 | 6.2±4.1 | -0.5±0.6 | 7.52±0.6 | 90.5±17 | | -2.39±0.42 | |
|  |  |  | lixisenatide 20ug Qd+met | 55 | 55.4±9.9 | 50.9 | 32.0±4.3 | 6.4±6.8 | -0.69±0.7 | 7.58±0.7 | 89.4±17 | | -3.01±0.41 | |
|  |  |  | lixisenatide 30ug Qd+met | 54 | 56.5±8.7 | 50 | 31.6±3.6 | 6.0±4.8 | -0.76±0.7 | 7.52±0.7 | 87.6±15 | | -3.47±0.41 | |
|  |  |  | lixisenatide 5ug bid+met | 53 | 57.1±8.2 | 47.2 | 31.6±4.2 | 6.2±6.0 | -0.65±0.6 | 7.60±0.6 | 86.5±14 | | -2.1±0.41 | |
|  |  |  | lixisenatide 10ug bid+met | 56 | 56.0±7.9 | 51.8 | 32.8±4.4 | 6.4±5.0 | -0.78±0.6 | 7.54±0.6 | 89.8±17 | | -2.21±0.41 | |
|  |  |  | lixisenatide 20ug bid+met | 54 | 56.7±8.3 | 37 | 32.7±4.4 | 6.6±5.1 | -0.75±0.7 | 7.61±0.7 | 88.5±17 | | -2.61±0.41 | |
|  |  |  | lixisenatide 30ug bid+met | 54 | 55.3±9.1 | 42.6 | 32.3±4.5 | 7.0±5.4 | -0.87±0.5 | 7.46±0.5 | 87.5±13 | | -3.89±0.41 | |
|  |  |  | PBO | 109 | 56.3±9.2 | 56 | 31.7±4.2 | 7.1±5.4 | -0.18±0.6 | 7.53±0.6 | 87.7±14 | | -1.94±0.32 | |
| Russell-Jones, 2009[^224^](#_ENREF_224) | | 26 weeks | Liraglutide 1.8mg qd+met+glimepide | 230 | 57.6±9.5 | 57 | 30.4±5.3 | 9.2±5.8 | -1.33±0.9 | 8.3±0.9 | 85.5±19.4 | | -1.8±0.33 | |
|  |  |  | PBO+met+glimepide | 114 | 57.5±9.6 | 49 | 31.3±5.0 | 9.4±6.2 | -0.24±0.9 | 8.3±0.9 | 85.7±16.7 | | -0.42±0.39 | |
| Umpierrez, 2011[^225^](#_ENREF_225) | | 16 weeks | LY 2189265 0.5/1.0mg | 66 | 59±12 | 53 | 33.7±4.1 | 9.0±7.6 | -1.32±0.12 | 8.25±0.9 | 94.8±17 | | -1.44±0.39 | |
|  |  |  | LY 2189265 1.0/1.0mg | 65 | 57±12 | 54 | 33.9±4.0 | 8.1±5.4 | -1.32±0.12 | 8.25±1.0 | 96.7±17 | | -1.34±0.39 | |
|  |  |  | LY 2189265 1.0/2.0mg | 65 | 54±11 | 52 | 34.2±4.1 | 8.6±6.9 | -1.59±0.12 | 8.43±1.0 | 98.6±18.4 | | -2.55±0.4 | |
|  |  |  | PBO | 66 | 56±12 | 44 | 33.9±4.3 | 7.5±5.4 | -0.3±0.8 | 8.05±0.8 | 94.7±15 | | -0.12±0.39 | |
| Zinman, 2007[^226^](#_ENREF_226) | | 16 weeks | Exenatide 10ug bid+TZD±metformin | 121 | 55.6±10.8 | 53.7 | 34.0±5.1 | 7.3±4.9 | -0.89±0.9 | 7.9±0.9 | 97.5±18.8 | | -1.75±0.65 | |
|  |  |  | PBO+TZD±metformin | 112 | 53.7±10.2 | 57.1 | 34.0±5.0 | 8.2±5.8 | 0.09±0.8 | 7.9±0.8 | 96.9±19.0 | | -0.24±0.65 | |
| Zinman, 2009[^227^](#_ENREF_227) | | 26 weeks | liraglutide 1.2mg/day+met 1g bid+RSG 4mg bid | 178 | 55±10 | 57 | 33.3±5.4 | 9±6 | -1.5±0.1 | 8.5±1.2 | / | | -1.0±0.3 | |
|  |  |  | liraglutide 1.8mg/day+met 1g bid+RSG 4mg bid | 178 | 55±11 | 51 | 33.5±5.1 | 9±6 | -1.5±0.1 | 8.6±1.2 | / | | -2±0.3 | |
|  |  |  | PBO+met 1g bid  +RSG 4mg bid | 177 | 55±10 | 62 | 33.9±5.2 | 9±6 | -0.5±0.1 | 8.4±1.2 | / | | 0.6±0.3 | |

**Supplement references:**

1. Ebeling P, Teppo AM, Koistinen HA, Koivisto VA. Concentration of the complement activation product, acylation-stimulating protein, is related to C-reactive protein in patients with type 2 diabetes. Metabolism: clinical and experimental 2001;50:283-7.

2. Hoffmann J, Spengler M. Efficacy of 24-week monotherapy with acarbose, glibenclamide, or placebo in NIDDM patients. The Essen Study. Diabetes care 1994;17:561-6.

3. Segal P, Feig PU, Schernthaner G, et al. The efficacy and safety of miglitol therapy compared with glibenclamide in patients with NIDDM inadequately controlled by diet alone. Diabetes care 1997;20:687-91.

4. Simonson DC, Kourides IA, Feinglos M, Shamoon H, Fischette CT. Efficacy, safety, and dose-response characteristics of glipizide gastrointestinal therapeutic system on glycemic control and insulin secretion in NIDDM. Results of two multicenter, randomized, placebo-controlled clinical trials. The Glipizide Gastrointestinal Therapeutic System Study Group. Diabetes care 1997;20:597-606.

5. Burant CF, Viswanathan P, Marcinak J, et al. TAK-875 versus placebo or glimepiride in type 2 diabetes mellitus: a phase 2, randomised, double-blind, placebo-controlled trial. Lancet 2012;379:1403-11.

6. Camerini-Davalos RA, Bloodworth JM, Jr., Velasco CA, Reddi AS. Effect of insulin-glipizide combination on skeletal muscle capillary basement membrane width in diabetic patients. Clinical therapeutics 1994;16:952-61.

7. Feinglos M, Dailey G, Cefalu W, et al. Effect on glycemic control of the addition of 2.5 mg glipizide GITS to metformin in patients with T2DM. Diabetes research and clinical practice 2005;68:167-75.

8. Nauck M, Frid A, Hermansen K, et al. Efficacy and safety comparison of liraglutide, glimepiride, and placebo, all in combination with metformin, in type 2 diabetes: the LEAD (liraglutide effect and action in diabetes)-2 study. Diabetes care 2009;32:84-90.

9. Riddle MC, Schneider J. Beginning insulin treatment of obese patients with evening 70/30 insulin plus glimepiride versus insulin alone. Glimepiride Combination Group. Diabetes care 1998;21:1052-7.

10. Roberts VL, Stewart J, Issa M, Lake B, Melis R. Triple therapy with glimepiride in patients with type 2 diabetes mellitus inadequately controlled by metformin and a thiazolidinedione: results of a 30-week, randomized, double-blind, placebo-controlled, parallel-group study. Clinical therapeutics 2005;27:1535-47.

11. Chiasson JL, Naditch L. The synergistic effect of miglitol plus metformin combination therapy in the treatment of type 2 diabetes. Diabetes care 2001;24:989-94.

12. DeFronzo RA, Goodman AM. Efficacy of metformin in patients with non-insulin-dependent diabetes mellitus. The Multicenter Metformin Study Group. The New England journal of medicine 1995;333:541-9.

13. Dornan TL, Heller SR, Peck GM, Tattersall RB. Double-blind evaluation of efficacy and tolerability of metformin in NIDDM. Diabetes care 1991;14:342-4.

14. Fujioka K, Brazg RL, Raz I, et al. Efficacy, dose-response relationship and safety of once-daily extended-release metformin (Glucophage XR) in type 2 diabetic patients with inadequate glycaemic control despite prior treatment with diet and exercise: results from two double-blind, placebo-controlled studies. Diabetes, obesity & metabolism 2005;7:28-39.

15. Hallsten K, Virtanen KA, Lonnqvist F, et al. Rosiglitazone but not metformin enhances insulin- and exercise-stimulated skeletal muscle glucose uptake in patients with newly diagnosed type 2 diabetes. Diabetes 2002;51:3479-85.

16. Horton ES, Clinkingbeard C, Gatlin M, Foley J, Mallows S, Shen S. Nateglinide alone and in combination with metformin improves glycemic control by reducing mealtime glucose levels in type 2 diabetes. Diabetes care 2000;23:1660-5.

17. Tessari P, Biolo G, Bruttomesso D, et al. Effects of metformin treatment on whole-body and splanchnic amino acid turnover in mild type 2 diabetes. The Journal of clinical endocrinology and metabolism 1994;79:1553-60.

18. Viljanen AP, Virtanen KA, Jarvisalo MJ, et al. Rosiglitazone treatment increases subcutaneous adipose tissue glucose uptake in parallel with perfusion in patients with type 2 diabetes: a double-blind, randomized study with metformin. The Journal of clinical endocrinology and metabolism 2005;90:6523-8.

19. Douek IF, Allen SE, Ewings P, Gale EA, Bingley PJ. Continuing metformin when starting insulin in patients with Type 2 diabetes: a double-blind randomized placebo-controlled trial. Diabetic medicine : a journal of the British Diabetic Association 2005;22:634-40.

20. Gram J, Henriksen JE, Grodum E, et al. Pharmacological treatment of the pathogenetic defects in type 2 diabetes: the randomized multicenter South Danish Diabetes Study. Diabetes care 2011;34:27-33.

21. Hermann LS, Kalen J, Katzman P, et al. Long-term glycaemic improvement after addition of metformin to insulin in insulin-treated obese type 2 diabetes patients. Diabetes, obesity & metabolism 2001;3:428-34.

22. Kooy A, de Jager J, Lehert P, et al. Long-term effects of metformin on metabolism and microvascular and macrovascular disease in patients with type 2 diabetes mellitus. Archives of internal medicine 2009;169:616-25.

23. Calle-Pascual A, Garcia-Honduvilla J, Martin-Alvarez PJ, Calle JR, Maranes JP. Influence of 16-week monotherapy with acarbose on cardiovascular risk factors in obese subjects with non-insulin-dependent diabetes mellitus: a controlled, double-blind comparison study with placebo. Diabetes & metabolism 1996;22:201-2.

24. Chiasson JL, Josse RG, Hunt JA, et al. The efficacy of acarbose in the treatment of patients with non-insulin-dependent diabetes mellitus. A multicenter controlled clinical trial. Annals of internal medicine 1994;121:928-35.

25. Coniff RF, Shapiro JA, Seaton TB. Long-term efficacy and safety of acarbose in the treatment of obese subjects with non-insulin-dependent diabetes mellitus. Archives of internal medicine 1994;154:2442-8.

26. Coniff RF, Shapiro JA, Robbins D, et al. Reduction of glycosylated hemoglobin and postprandial hyperglycemia by acarbose in patients with NIDDM. A placebo-controlled dose-comparison study. Diabetes care 1995;18:817-24.

27. Coniff RF, Shapiro JA, Seaton TB, Bray GA. Multicenter, placebo-controlled trial comparing acarbose (BAY g 5421) with placebo, tolbutamide, and tolbutamide-plus-acarbose in non-insulin-dependent diabetes mellitus. The American journal of medicine 1995;98:443-51.

28. Delgado H, Lehmann T, Bobbioni-Harsch E, Ybarra J, Golay A. Acarbose improves indirectly both insulin resistance and secretion in obese type 2 diabetic patients. Diabetes & metabolism 2002;28:195-200.

29. Fischer S, Patzak A, Rietzsch H, et al. Influence of treatment with acarbose or glibenclamide on insulin sensitivity in type 2 diabetic patients. Diabetes, obesity & metabolism 2003;5:38-44.

30. Hanefeld M, Haffner SM, Menschikowski M, et al. Different effects of acarbose and glibenclamide on proinsulin and insulin profiles in people with Type 2 diabetes. Diabetes research and clinical practice 2002;55:221-7.

31. Hanefeld M, Schaper F, Koehler C, et al. Effect of acarbose on postmeal mononuclear blood cell response in patients with early type 2 diabetes: the AI(I)DA study. Hormone and metabolic research = Hormon- und Stoffwechselforschung = Hormones et metabolisme 2009;41:132-6.

32. Hasche H, Mertes G, Bruns C, et al. Effects of acarbose treatment in Type 2 diabetic patients under dietary training: a multicentre, double-blind, placebo-controlled, 2-year study. Diabetes, nutrition & metabolism 1999;12:277-85.

33. Hoffmann J, Spengler M. Efficacy of 24-week monotherapy with acarbose, metformin, or placebo in dietary-treated NIDDM patients: the Essen-II Study. The American journal of medicine 1997;103:483-90.

34. Josse RG, Chiasson JL, Ryan EA, et al. Acarbose in the treatment of elderly patients with type 2 diabetes. Diabetes research and clinical practice 2003;59:37-42.

35. Meneilly GS, Ryan EA, Radziuk J, et al. Effect of acarbose on insulin sensitivity in elderly patients with diabetes. Diabetes care 2000;23:1162-7.

36. Rosenbaum P, Peres RB, Zanella MT, Ferreira SR. Improved glycemic control by acarbose therapy in hypertensive diabetic patients: effects on blood pressure and hormonal parameters. Brazilian journal of medical and biological research = Revista brasileira de pesquisas medicas e biologicas / Sociedade Brasileira de Biofisica [et al] 2002;35:877-84.

37. Scott R, Lintott CJ, Zimmet P, Campbell L, Bowen K, Welborn T. Will acarbose improve the metabolic abnormalities of insulin-resistant type 2 diabetes mellitus? Diabetes research and clinical practice 1999;43:179-85.

38. Wagner H, Degerblad M, Thorell A, et al. Combined treatment with exercise training and acarbose improves metabolic control and cardiovascular risk factor profile in subjects with mild type 2 diabetes. Diabetes care 2006;29:1471-7.

39. Bachmann W, Petzinna D, Raptis SA, Wascher T, Westermeier T. Long-term improvement of metabolic control by acarbose in type 2 diabetes patients poorly controlled with maximum sulfonylurea therapy. Clinical drug investigation 2003;23:679-86.

40. Halimi S, Le Berre MA, Grange V. Efficacy and safety of acarbose add-on therapy in the treatment of overweight patients with Type 2 diabetes inadequately controlled with metformin: a double-blind, placebo-controlled study. Diabetes research and clinical practice 2000;50:49-56.

41. Kelley DE, Bidot P, Freedman Z, et al. Efficacy and safety of acarbose in insulin-treated patients with type 2 diabetes. Diabetes care 1998;21:2056-61.

42. Johnston PS, Feig PU, Coniff RF, Krol A, Kelley DE, Mooradian AD. Chronic treatment of African-American type 2 diabetic patients with alpha-glucosidase inhibition. Diabetes care 1998;21:416-22.

43. Johnston PS, Feig PU, Coniff RF, Krol A, Davidson JA, Haffner SM. Long-term titrated-dose alpha-glucosidase inhibition in non-insulin-requiring Hispanic NIDDM patients. Diabetes care 1998;21:409-15.

44. Lam KS, Tiu SC, Tsang MW, Ip TP, Tam SC. Acarbose in NIDDM patients with poor control on conventional oral agents. A 24-week placebo-controlled study. Diabetes care 1998;21:1154-8.

45. Mitrakou A, Tountas N, Raptis AE, Bauer RJ, Schulz H, Raptis SA. Long-term effectiveness of a new alpha-glucosidase inhibitor (BAY m1099-miglitol) in insulin-treated type 2 diabetes mellitus. Diabetic medicine : a journal of the British Diabetic Association 1998;15:657-60.

46. Phillips P, Karrasch J, Scott R, Wilson D, Moses R. Acarbose improves glycemic control in overweight type 2 diabetic patients insufficiently treated with metformin. Diabetes care 2003;26:269-73.

47. Schnell O, Mertes G, Standl E. Acarbose and metabolic control in patients with type 2 diabetes with newly initiated insulin therapy. Diabetes, obesity & metabolism 2007;9:853-8.

48. Standl E, Baumgartl HJ, Fuchtenbusch M, Stemplinger J. Effect of acarbose on additional insulin therapy in type 2 diabetic patients with late failure of sulphonylurea therapy. Diabetes, obesity & metabolism 1999;1:215-20.

49. Standl E, Schernthaner G, Rybka J, Hanefeld M, Raptis SA, Naditch L. Improved glycaemic control with miglitol in inadequately-controlled type 2 diabetics. Diabetes research and clinical practice 2001;51:205-13.

50. Van Gaal L, Maislos M, Schernthaner G, Rybka J, Segal P. Miglitol combined with metformin improves glycaemic control in type 2 diabetes. Diabetes, obesity & metabolism 2001;3:326-31.

51. Carey DG, Cowin GJ, Galloway GJ, et al. Effect of rosiglitazone on insulin sensitivity and body composition in type 2 diabetic patients [corrected]. Obesity research 2002;10:1008-15.

52. Chou HS, Truitt KE, Moberly JB, et al. A 26-week, placebo- and pioglitazone-controlled monotherapy study of rivoglitazone in subjects with type 2 diabetes mellitus. Diabetes, obesity & metabolism 2012;14:1000-9.

53. Ebeling P, Teppo AM, Koistinen HA, et al. Troglitazone reduces hyperglycaemia and selectively acute-phase serum proteins in patients with Type II diabetes. Diabetologia 1999;42:1433-8.

54. Fonseca VA, Reynolds T, Hemphill D, et al. Effect of troglitazone on fibrinolysis and activated coagulation in patients with non-insulin-dependent diabetes mellitus. Journal of diabetes and its complications 1998;12:181-6.

55. Gastaldelli A, Miyazaki Y, Pettiti M, et al. The effect of rosiglitazone on the liver: decreased gluconeogenesis in patients with type 2 diabetes. The Journal of clinical endocrinology and metabolism 2006;91:806-12.

56. Gastaldelli A, Ferrannini E, Miyazaki Y, Matsuda M, Mari A, DeFronzo RA. Thiazolidinediones improve beta-cell function in type 2 diabetic patients. American journal of physiology Endocrinology and metabolism 2007;292:E871-83.

57. Gastaldelli A, Casolaro A, Ciociaro D, et al. Decreased whole body lipolysis as a mechanism of the lipid-lowering effect of pioglitazone in type 2 diabetic patients. American journal of physiology Endocrinology and metabolism 2009;297:E225-30.

58. Haffner SM, Greenberg AS, Weston WM, Chen H, Williams K, Freed MI. Effect of rosiglitazone treatment on nontraditional markers of cardiovascular disease in patients with type 2 diabetes mellitus. Circulation 2002;106:679-84.

59. Herz M, Johns D, Reviriego J, et al. A randomized, double-blind, placebo-controlled, clinical trial of the effects of pioglitazone on glycemic control and dyslipidemia in oral antihyperglycemic medication-naive patients with type 2 diabetes mellitus. Clinical therapeutics 2003;25:1074-95.

60. Iwamoto Y, Kosaka K, Kuzuya T, Akanuma Y, Shigeta Y, Kaneko T. Effects of troglitazone: a new hypoglycemic agent in patients with NIDDM poorly controlled by diet therapy. Diabetes care 1996;19:151-6.

61. Juhl CB, Hollingdal M, Porksen N, Prange A, Lonnqvist F, Schmitz O. Influence of rosiglitazone treatment on beta-cell function in type 2 diabetes: evidence of an increased ability of glucose to entrain high-frequency insulin pulsatility. The Journal of clinical endocrinology and metabolism 2003;88:3794-800.

62. Khan M, Murray FT, Karunaratne M, Perez A. Pioglitazone and reductions in post-challenge glucose levels in patients with type 2 diabetes. Diabetes, obesity & metabolism 2006;8:31-8.

63. Kong AP, Yamasaki A, Ozaki R, et al. A randomized-controlled trial to investigate the effects of rivoglitazone, a novel PPAR gamma agonist on glucose-lipid control in type 2 diabetes. Diabetes, obesity & metabolism 2011;13:806-13.

64. Kumar S, Boulton AJ, Beck-Nielsen H, et al. Troglitazone, an insulin action enhancer, improves metabolic control in NIDDM patients. Troglitazone Study Group. Diabetologia 1996;39:701-9.

65. Lautamaki R, Airaksinen KE, Seppanen M, et al. Rosiglitazone improves myocardial glucose uptake in patients with type 2 diabetes and coronary artery disease: a 16-week randomized, double-blind, placebo-controlled study. Diabetes 2005;54:2787-94.

66. Miyazaki Y, Glass L, Triplitt C, et al. Effect of rosiglitazone on glucose and non-esterified fatty acid metabolism in Type II diabetic patients. Diabetologia 2001;44:2210-9.

67. Miyazaki Y, Mahankali A, Matsuda M, et al. Improved glycemic control and enhanced insulin sensitivity in type 2 diabetic subjects treated with pioglitazone. Diabetes care 2001;24:710-9.

68. Miyazaki Y, Matsuda M, DeFronzo RA. Dose-response effect of pioglitazone on insulin sensitivity and insulin secretion in type 2 diabetes. Diabetes care 2002;25:517-23.

69. Natali A, Baldeweg S, Toschi E, et al. Vascular effects of improving metabolic control with metformin or rosiglitazone in type 2 diabetes. Diabetes care 2004;27:1349-57.

70. Oz O, Tuncel E, Eryilmaz S, et al. Arterial elasticity and plasma levels of adiponectin and leptin in type 2 diabetic patients treated with thiazolidinediones. Endocrine 2008;33:101-5.

71. Oz Gul O, Tuncel E, Yilmaz Y, et al. Comparative effects of pioglitazone and rosiglitazone on plasma levels of soluble receptor for advanced glycation end products in type 2 diabetes mellitus patients. Metabolism: clinical and experimental 2010;59:64-9.

72. Patel J, Anderson RJ, Rappaport EB. Rosiglitazone monotherapy improves glycaemic control in patients with type 2 diabetes: a twelve-week, randomized, placebo-controlled study. Diabetes, obesity & metabolism 1999;1:165-72.

73. Phillips LS, Grunberger G, Miller E, Patwardhan R, Rappaport EB, Salzman A. Once- and twice-daily dosing with rosiglitazone improves glycemic control in patients with type 2 diabetes. Diabetes care 2001;24:308-15.

74. Raskin P, Rappaport EB, Cole ST, Yan Y, Patwardhan R, Freed MI. Rosiglitazone short-term monotherapy lowers fasting and post-prandial glucose in patients with type II diabetes. Diabetologia 2000;43:278-84.

75. Rosenblatt S, Miskin B, Glazer NB, Prince MJ, Robertson KE. The impact of pioglitazone on glycemic control and atherogenic dyslipidemia in patients with type 2 diabetes mellitus. Coronary artery disease 2001;12:413-23.

76. Rosenstock J, Shen SG, Gatlin MR, Foley JE. Combination therapy with nateglinide and a thiazolidinedione improves glycemic control in type 2 diabetes. Diabetes care 2002;25:1529-33.

77. Scherbaum WA, Goke B. Metabolic efficacy and safety of once-daily pioglitazone monotherapy in patients with type 2 diabetes: a double-blind, placebo-controlled study. Hormone and metabolic research = Hormon- und Stoffwechselforschung = Hormones et metabolisme 2002;34:589-95.

78. Tan GD, Fielding BA, Currie JM, et al. The effects of rosiglitazone on fatty acid and triglyceride metabolism in type 2 diabetes. Diabetologia 2005;48:83-95.

79. Truitt KE, Goldberg RB, Rosenstock J, et al. A 26-week, placebo- and pioglitazone-controlled, dose-ranging study of rivoglitazone, a novel thiazolidinedione for the treatment of type 2 diabetes. Current medical research and opinion 2010;26:1321-31.

80. Wallace TM, Levy JC, Matthews DR. An increase in insulin sensitivity and basal beta-cell function in diabetic subjects treated with pioglitazone in a placebo-controlled randomized study. Diabetic medicine : a journal of the British Diabetic Association 2004;21:568-76.

81. Barnett AH, Grant PJ, Hitman GA, et al. Rosiglitazone in Type 2 diabetes mellitus: an evaluation in British Indo-Asian patients. Diabetic medicine : a journal of the British Diabetic Association 2003;20:387-93.

82. Berhanu P, Perez A, Yu S. Effect of pioglitazone in combination with insulin therapy on glycaemic control, insulin dose requirement and lipid profile in patients with type 2 diabetes previously poorly controlled with combination therapy. Diabetes, obesity & metabolism 2007;9:512-20.

83. Bertrand OF, Poirier P, Rodes-Cabau J, et al. Cardiometabolic effects of rosiglitazone in patients with type 2 diabetes and coronary artery bypass grafts: A randomized placebo-controlled clinical trial. Atherosclerosis 2010;211:565-73.

84. Brackenridge AL, Jackson N, Jefferson W, et al. Effects of rosiglitazone and pioglitazone on lipoprotein metabolism in patients with Type 2 diabetes and normal lipids. Diabetic medicine : a journal of the British Diabetic Association 2009;26:532-9.

85. Buras J, Reenstra WR, Orlow D, Horton ES, Veves A. Troglitazone-induced changes in adiponectin do not affect endothelial function in diabetes. Obesity research 2005;13:1167-74.

86. Buse JB, Gumbiner B, Mathias NP, Nelson DM, Faja BW, Whitcomb RW. Troglitazone use in insulin-treated type 2 diabetic patients. The Troglitazone Insulin Study Group. Diabetes care 1998;21:1455-61.

87. Charpentier G, Halimi S. Earlier triple therapy with pioglitazone in patients with type 2 diabetes. Diabetes, obesity & metabolism 2009;11:844-54.

88. Dailey GE, 3rd, Noor MA, Park JS, Bruce S, Fiedorek FT. Glycemic control with glyburide/metformin tablets in combination with rosiglitazone in patients with type 2 diabetes: a randomized, double-blind trial. The American journal of medicine 2004;116:223-9.

89. Davidson JA, McMorn SO, Waterhouse BR, Cobitz AR. A 24-week, multicenter, randomized, double-blind, placebo-controlled, parallel-group study of the efficacy and tolerability of combination therapy with rosiglitazone and sulfonylurea in African American and Hispanic American patients with type 2 diabetes inadequately controlled with sulfonylurea monotherapy. Clinical therapeutics 2007;29:1900-14.

90. Derosa G, Salvadeo SA, D'Angelo A, et al. Rosiglitazone therapy improves insulin resistance parameters in overweight and obese diabetic patients intolerant to metformin. Archives of medical research 2008;39:412-9.

91. Einhorn D, Rendell M, Rosenzweig J, Egan JW, Mathisen AL, Schneider RL. Pioglitazone hydrochloride in combination with metformin in the treatment of type 2 diabetes mellitus: a randomized, placebo-controlled study. The Pioglitazone 027 Study Group. Clinical therapeutics 2000;22:1395-409.

92. Fonseca V, Rosenstock J, Patwardhan R, Salzman A. Effect of metformin and rosiglitazone combination therapy in patients with type 2 diabetes mellitus: a randomized controlled trial. Jama 2000;283:1695-702.

93. Galle J, Kleophas W, Dellanna F, et al. Comparison of the Effects of Pioglitazone versus Placebo when Given in Addition to Standard Insulin Treatment in Patients with Type 2 Diabetes Mellitus Requiring Hemodialysis: Results from the PIOren Study. Nephron extra 2012;2:104-14.

94. Gomez-Perez FJ, Fanghanel-Salmon G, Antonio Barbosa J, et al. Efficacy and safety of rosiglitazone plus metformin in Mexicans with type 2 diabetes. Diabetes/metabolism research and reviews 2002;18:127-34.

95. Grey A, Beckley V, Doyle A, et al. Pioglitazone increases bone marrow fat in type 2 diabetes: results from a randomized controlled trial. European journal of endocrinology / European Federation of Endocrine Societies 2012;166:1087-91.

96. Henriksen K, Byrjalsen I, Qvist P, et al. Efficacy and safety of the PPARgamma partial agonist balaglitazone compared with pioglitazone and placebo: a phase III, randomized, parallel-group study in patients with type 2 diabetes on stable insulin therapy. Diabetes/metabolism research and reviews 2011;27:392-401.

97. Hollander P, Yu D, Chou HS. Low-dose rosiglitazone in patients with insulin-requiring type 2 diabetes. Archives of internal medicine 2007;167:1284-90.

98. Hwang YC, Lee EY, Lee WJ, et al. Effects of rosiglitazone on body fat distribution and insulin sensitivity in Korean type 2 diabetes mellitus patients. Metabolism: clinical and experimental 2008;57:479-87.

99. Iwamoto Y, Kosaka K, Kuzuya T, Akanuma Y, Shigeta Y, Kaneko T. Effect of combination therapy of troglitazone and sulphonylureas in patients with Type 2 diabetes who were poorly controlled by sulphonylurea therapy alone. Diabetic medicine : a journal of the British Diabetic Association 1996;13:365-70.

100. Kaku K. Efficacy and safety of therapy with metformin plus pioglitazone in the treatment of patients with type 2 diabetes: a double-blind, placebo-controlled, clinical trial. Current medical research and opinion 2009;25:1111-9.

101. Kawamori R, Matsuhisa M, Kinoshita J, et al. Pioglitazone enhances splanchnic glucose uptake as well as peripheral glucose uptake in non-insulin-dependent diabetes mellitus. AD-4833 Clamp-OGL Study Group. Diabetes research and clinical practice 1998;41:35-43.

102. Kelly IE, Han TS, Walsh K, Lean ME. Effects of a thiazolidinedione compound on body fat and fat distribution of patients with type 2 diabetes. Diabetes care 1999;22:288-93.

103. Kipnes MS, Krosnick A, Rendell MS, Egan JW, Mathisen AL, Schneider RL. Pioglitazone hydrochloride in combination with sulfonylurea therapy improves glycemic control in patients with type 2 diabetes mellitus: a randomized, placebo-controlled study. The American journal of medicine 2001;111:10-7.

104. Marre M, Shaw J, Brandle M, et al. Liraglutide, a once-daily human GLP-1 analogue, added to a sulphonylurea over 26 weeks produces greater improvements in glycaemic and weight control compared with adding rosiglitazone or placebo in subjects with Type 2 diabetes (LEAD-1 SU). Diabetic medicine : a journal of the British Diabetic Association 2009;26:268-78.

105. Mattoo V, Eckland D, Widel M, et al. Metabolic effects of pioglitazone in combination with insulin in patients with type 2 diabetes mellitus whose disease is not adequately controlled with insulin therapy: results of a six-month, randomized, double-blind, prospective, multicenter, parallel-group study. Clinical therapeutics 2005;27:554-67.

106. Mimura K, Umeda F, Hiramatsu S, et al. Effects of a new oral hypoglycaemic agent (CS-045) on metabolic abnormalities and insulin resistance in type 2 diabetes. Diabetic medicine : a journal of the British Diabetic Association 1994;11:685-91.

107. Negro R, Mangieri T, Dazzi D, Pezzarossa A, Hassan H. Rosiglitazone effects on blood pressure and metabolic parameters in nondipper diabetic patients. Diabetes research and clinical practice 2005;70:20-5.

108. Osende JI, Badimon JJ, Fuster V, et al. Blood thrombogenicity in type 2 diabetes mellitus patients is associated with glycemic control. Journal of the American College of Cardiology 2001;38:1307-12.

109. Raskin P, Rendell M, Riddle MC, Dole JF, Freed MI, Rosenstock J. A randomized trial of rosiglitazone therapy in patients with inadequately controlled insulin-treated type 2 diabetes. Diabetes care 2001;24:1226-32.

110. Reynolds LR, Konz EC, Frederich RC, Anderson JW. Rosiglitazone amplifies the benefits of lifestyle intervention measures in long-standing type 2 diabetes mellitus. Diabetes, obesity & metabolism 2002;4:270-5.

111. Rosenstock J, Chou HS, Matthaei S, Seidel DK, Hamann A. Potential benefits of early addition of rosiglitazone in combination with glimepiride in the treatment of type 2 diabetes. Diabetes, obesity & metabolism 2008;10:862-73.

112. Scheen AJ, Tan MH, Betteridge DJ, Birkeland K, Schmitz O, Charbonnel B. Long-term glycaemic effects of pioglitazone compared with placebo as add-on treatment to metformin or sulphonylurea monotherapy in PROactive (PROactive 18). Diabetic medicine : a journal of the British Diabetic Association 2009;26:1242-9.

113. Schwartz S, Raskin P, Fonseca V, Graveline JF. Effect of troglitazone in insulin-treated patients with type II diabetes mellitus. Troglitazone and Exogenous Insulin Study Group. The New England journal of medicine 1998;338:861-6.

114. Smith SR, De Jonge L, Volaufova J, Li Y, Xie H, Bray GA. Effect of pioglitazone on body composition and energy expenditure: a randomized controlled trial. Metabolism: clinical and experimental 2005;54:24-32.

115. Sridhar S, Walia R, Sachdeva N, Bhansali A. Effect of pioglitazone on testosterone in eugonadal men with type 2 diabetes mellitus: a randomized double-blind placebo-controlled study. Clinical endocrinology 2013;78:454-9.

116. Wolffenbuttel BH, Gomis R, Squatrito S, Jones NP, Patwardhan RN. Addition of low-dose rosiglitazone to sulphonylurea therapy improves glycaemic control in Type 2 diabetic patients. Diabetic medicine : a journal of the British Diabetic Association 2000;17:40-7.

117. Yale JF, Valiquett TR, Ghazzi MN, Owens-Grillo JK, Whitcomb RW, Foyt HL. The effect of a thiazolidinedione drug, troglitazone, on glycemia in patients with type 2 diabetes mellitus poorly controlled with sulfonylurea and metformin. A multicenter, randomized, double-blind, placebo-controlled trial. Annals of internal medicine 2001;134:737-45.

118. Yang WS, Jeng CY, Wu TJ, et al. Synthetic peroxisome proliferator-activated receptor-gamma agonist, rosiglitazone, increases plasma levels of adiponectin in type 2 diabetic patients. Diabetes care 2002;25:376-80.

119. Zhu XX, Pan CY, Li GW, et al. Addition of rosiglitazone to existing sulfonylurea treatment in chinese patients with type 2 diabetes and exposure to hepatitis B or C. Diabetes technology & therapeutics 2003;5:33-42.

120. Dejager S, Razac S, Foley JE, Schweizer A. Vildagliptin in drug-naive patients with type 2 diabetes: a 24-week, double-blind, randomized, placebo-controlled, multiple-dose study. Hormone and metabolic research = Hormon- und Stoffwechselforschung = Hormones et metabolisme 2007;39:218-23.

121. Frederich R, McNeill R, Berglind N, Fleming D, Chen R. The efficacy and safety of the dipeptidyl peptidase-4 inhibitor saxagliptin in treatment-naive patients with type 2 diabetes mellitus: a randomized controlled trial. Diabetology & metabolic syndrome 2012;4:36.

122. Haak T, Meinicke T, Jones R, Weber S, von Eynatten M, Woerle HJ. Initial combination of linagliptin and metformin improves glycaemic control in type 2 diabetes: a randomized, double-blind, placebo-controlled study. Diabetes, obesity & metabolism 2012;14:565-74.

123. Hanefeld M, Herman GA, Wu M, Mickel C, Sanchez M, Stein PP. Once-daily sitagliptin, a dipeptidyl peptidase-4 inhibitor, for the treatment of patients with type 2 diabetes. Current medical research and opinion 2007;23:1329-39.

124. Inagaki N, Onouchi H, Sano H, Funao N, Kuroda S, Kaku K. SYR-472, a novel once-weekly dipeptidyl peptidase-4 (DPP-4) inhibitor, in type 2 diabetes mellitus: a phase 2, randomised, double-blind, placebo-controlled trial. The lancet Diabetes & endocrinology 2014;2:125-32.

125. Iwamoto Y, Taniguchi T, Nonaka K, et al. Dose-ranging efficacy of sitagliptin, a dipeptidyl peptidase-4 inhibitor, in Japanese patients with type 2 diabetes mellitus. Endocrine journal 2010;57:383-94.

126. Kawamori R, Inagaki N, Araki E, et al. Linagliptin monotherapy provides superior glycaemic control versus placebo or voglibose with comparable safety in Japanese patients with type 2 diabetes: a randomized, placebo and active comparator-controlled, double-blind study. Diabetes, obesity & metabolism 2012;14:348-57.

127. Kikuchi M, Abe N, Kato M, Terao S, Mimori N, Tachibana H. Vildagliptin dose-dependently improves glycemic control in Japanese patients with type 2 diabetes mellitus. Diabetes research and clinical practice 2009;83:233-40.

128. Mohan V, Yang W, Son HY, et al. Efficacy and safety of sitagliptin in the treatment of patients with type 2 diabetes in China, India, and Korea. Diabetes research and clinical practice 2009;83:106-16.

129. Nonaka K, Kakikawa T, Sato A, et al. Efficacy and safety of sitagliptin monotherapy in Japanese patients with type 2 diabetes. Diabetes research and clinical practice 2008;79:291-8.

130. Del Prato S, Barnett AH, Huisman H, Neubacher D, Woerle HJ, Dugi KA. Effect of linagliptin monotherapy on glycaemic control and markers of beta-cell function in patients with inadequately controlled type 2 diabetes: a randomized controlled trial. Diabetes, obesity & metabolism 2011;13:258-67.

131. Pan CY, Yang W, Tou C, Gause-Nilsson I, Zhao J. Efficacy and safety of saxagliptin in drug-naive Asian patients with type 2 diabetes mellitus: a randomized controlled trial. Diabetes/metabolism research and reviews 2012;28:268-75.

132. Pi-Sunyer FX, Schweizer A, Mills D, Dejager S. Efficacy and tolerability of vildagliptin monotherapy in drug-naive patients with type 2 diabetes. Diabetes research and clinical practice 2007;76:132-8.

133. Bergenstal RM, Forti A, Chiasson JL, Woloschak M, Boldrin M, Balena R. Efficacy and safety of taspoglutide versus sitagliptin for type 2 diabetes mellitus (T-emerge 4 trial). Diabetes therapy : research, treatment and education of diabetes and related disorders 2012;3:13.

134. Ristic S, Byiers S, Foley J, Holmes D. Improved glycaemic control with dipeptidyl peptidase-4 inhibition in patients with type 2 diabetes: vildagliptin (LAF237) dose response. Diabetes, obesity & metabolism 2005;7:692-8.

135. Roden M, Weng J, Eilbracht J, et al. Empagliflozin monotherapy with sitagliptin as an active comparator in patients with type 2 diabetes: a randomised, double-blind, placebo-controlled, phase 3 trial. The lancet Diabetes & endocrinology 2013;1:208-19.

136. Rosenstock J, Aguilar-Salinas C, Klein E, Nepal S, List J, Chen R. Effect of saxagliptin monotherapy in treatment-naive patients with type 2 diabetes. Current medical research and opinion 2009;25:2401-11.

137. Rosenstock J, Sankoh S, List JF. Glucose-lowering activity of the dipeptidyl peptidase-4 inhibitor saxagliptin in drug-naive patients with type 2 diabetes. Diabetes, obesity & metabolism 2008;10:376-86.

138. Scherbaum WA, Schweizer A, Mari A, et al. Efficacy and tolerability of vildagliptin in drug-naive patients with type 2 diabetes and mild hyperglycaemia*. Diabetes, obesity & metabolism 2008;10:675-82.

139. Scott R, Wu M, Sanchez M, Stein P. Efficacy and tolerability of the dipeptidyl peptidase-4 inhibitor sitagliptin as monotherapy over 12 weeks in patients with type 2 diabetes. International journal of clinical practice 2007;61:171-80.

140. Seino Y, Fujita T, Hiroi S, Hirayama M, Kaku K. Efficacy and safety of alogliptin in Japanese patients with type 2 diabetes mellitus: a randomized, double-blind, dose-ranging comparison with placebo, followed by a long-term extension study. Current medical research and opinion 2011;27:1781-92.

141. Ahren B, Gomis R, Standl E, Mills D, Schweizer A. Twelve- and 52-week efficacy of the dipeptidyl peptidase IV inhibitor LAF237 in metformin-treated patients with type 2 diabetes. Diabetes care 2004;27:2874-80.

142. Barnett AH, Charbonnel B, Donovan M, Fleming D, Chen R. Effect of saxagliptin as add-on therapy in patients with poorly controlled type 2 diabetes on insulin alone or insulin combined with metformin. Current medical research and opinion 2012;28:513-23.

143. Barnett AH, Huisman H, Jones R, von Eynatten M, Patel S, Woerle HJ. Linagliptin for patients aged 70 years or older with type 2 diabetes inadequately controlled with common antidiabetes treatments: a randomised, double-blind, placebo-controlled trial. Lancet 2013;382:1413-23.

144. Bosi E, Camisasca RP, Collober C, Rochotte E, Garber AJ. Effects of vildagliptin on glucose control over 24 weeks in patients with type 2 diabetes inadequately controlled with metformin. Diabetes care 2007;30:890-5.

145. Bosi E, Ellis GC, Wilson CA, Fleck PR. Alogliptin as a third oral antidiabetic drug in patients with type 2 diabetes and inadequate glycaemic control on metformin and pioglitazone: a 52-week, randomized, double-blind, active-controlled, parallel-group study. Diabetes, obesity & metabolism 2011;13:1088-96.

146. DeFronzo RA, Burant CF, Fleck P, Wilson C, Mekki Q, Pratley RE. Efficacy and tolerability of the DPP-4 inhibitor alogliptin combined with pioglitazone, in metformin-treated patients with type 2 diabetes. The Journal of clinical endocrinology and metabolism 2012;97:1615-22.

147. Derosa G, Carbone A, D'Angelo A, et al. A randomized, double-blind, placebo-controlled trial evaluating sitagliptin action on insulin resistance parameters and beta-cell function. Expert opinion on pharmacotherapy 2012;13:2433-42.

148. Derosa G, Ragonesi PD, Carbone A, et al. Vildagliptin added to metformin on beta-cell function after a euglycemic hyperinsulinemic and hyperglycemic clamp in type 2 diabetes patients. Diabetes technology & therapeutics 2012;14:475-84.

149. Dobs AS, Goldstein BJ, Aschner P, et al. Efficacy and safety of sitagliptin added to ongoing metformin and rosiglitazone combination therapy in a randomized placebo-controlled 54-week trial in patients with type 2 diabetes. Journal of diabetes 2013;5:68-79.

150. Fonseca V, Schweizer A, Albrecht D, Baron MA, Chang I, Dejager S. Addition of vildagliptin to insulin improves glycaemic control in type 2 diabetes. Diabetologia 2007;50:1148-55.

151. Fonseca V, Staels B, Morgan JD, 2nd, et al. Efficacy and safety of sitagliptin added to ongoing metformin and pioglitazone combination therapy in a randomized, placebo-controlled, 26-week trial in patients with type 2 diabetes. Journal of diabetes and its complications 2013;27:177-83.

152. Forst T, Uhlig-Laske B, Ring A, et al. Linagliptin (BI 1356), a potent and selective DPP-4 inhibitor, is safe and efficacious in combination with metformin in patients with inadequately controlled Type 2 diabetes. Diabetic medicine : a journal of the British Diabetic Association 2010;27:1409-19.

153. Garber AJ, Schweizer A, Baron MA, Rochotte E, Dejager S. Vildagliptin in combination with pioglitazone improves glycaemic control in patients with type 2 diabetes failing thiazolidinedione monotherapy: a randomized, placebo-controlled study. Diabetes, obesity & metabolism 2007;9:166-74.

154. Garber AJ, Foley JE, Banerji MA, et al. Effects of vildagliptin on glucose control in patients with type 2 diabetes inadequately controlled with a sulphonylurea. Diabetes, obesity & metabolism 2008;10:1047-56.

155. Gomis R, Espadero RM, Jones R, Woerle HJ, Dugi KA. Efficacy and safety of initial combination therapy with linagliptin and pioglitazone in patients with inadequately controlled type 2 diabetes: a randomized, double-blind, placebo-controlled study. Diabetes, obesity & metabolism 2011;13:653-61.

156. Goodman M, Thurston H, Penman J. Efficacy and tolerability of vildagliptin in patients with type 2 diabetes inadequately controlled with metformin monotherapy. Hormone and metabolic research = Hormon- und Stoffwechselforschung = Hormones et metabolisme 2009;41:368-73.

157. Hermansen K, Kipnes M, Luo E, Fanurik D, Khatami H, Stein P. Efficacy and safety of the dipeptidyl peptidase-4 inhibitor, sitagliptin, in patients with type 2 diabetes mellitus inadequately controlled on glimepiride alone or on glimepiride and metformin. Diabetes, obesity & metabolism 2007;9:733-45.

158. Hollander P, Li J, Allen E, Chen R. Saxagliptin added to a thiazolidinedione improves glycemic control in patients with type 2 diabetes and inadequate control on thiazolidinedione alone. The Journal of clinical endocrinology and metabolism 2009;94:4810-9.

159. Jadzinsky M, Pfutzner A, Paz-Pacheco E, Xu Z, Allen E, Chen R. Saxagliptin given in combination with metformin as initial therapy improves glycaemic control in patients with type 2 diabetes compared with either monotherapy: a randomized controlled trial. Diabetes, obesity & metabolism 2009;11:611-22.

160. Kaku K, Itayasu T, Hiroi S, Hirayama M, Seino Y. Efficacy and safety of alogliptin added to pioglitazone in Japanese patients with type 2 diabetes: a randomized, double-blind, placebo-controlled trial with an open-label long-term extension study. Diabetes, obesity & metabolism 2011;13:1028-35.

161. Kadowaki T, Kondo K. Efficacy and safety of teneligliptin added to glimepiride in Japanese patients with type 2 diabetes mellitus: a randomized, double-blind, placebo-controlled study with an open-label, long-term extension. Diabetes, obesity & metabolism 2014;16:418-25.

162. Kikuchi M, Haneda M, Koya D, et al. Efficacy and tolerability of vildagliptin as an add-on to glimepiride in Japanese patients with Type 2 diabetes mellitus. Diabetes research and clinical practice 2010;89:216-23.

163. Kothny W, Shao Q, Groop PH, Lukashevich V. One-year safety, tolerability and efficacy of vildagliptin in patients with type 2 diabetes and moderate or severe renal impairment. Diabetes, obesity & metabolism 2012;14:1032-9.

164. Lavalle-Gonzalez FJ, Januszewicz A, Davidson J, et al. Efficacy and safety of canagliflozin compared with placebo and sitagliptin in patients with type 2 diabetes on background metformin monotherapy: a randomised trial. Diabetologia 2013;56:2582-92.

165. Lewin AJ, Arvay L, Liu D, Patel S, von Eynatten M, Woerle HJ. Efficacy and tolerability of linagliptin added to a sulfonylurea regimen in patients with inadequately controlled type 2 diabetes mellitus: an 18-week, multicenter, randomized, double-blind, placebo-controlled trial. Clinical therapeutics 2012;34:1909-19 e15.

166. Lukashevich V, Prato SD, Araga M, Kothny W. Efficacy and safety of vildagliptin in patients with type 2 diabetes mellitus inadequately controlled with dual combination of metformin and sulphonylurea. Diabetes, obesity & metabolism 2014;16:403-9.

167. Moses RG, Kalra S, Brook D, et al. A randomized controlled trial of the efficacy and safety of saxagliptin as add-on therapy in patients with type 2 diabetes and inadequate glycaemic control on metformin plus a sulphonylurea. Diabetes, obesity & metabolism 2014;16:443-50.

168. Nauck MA, Ellis GC, Fleck PR, Wilson CA, Mekki Q. Efficacy and safety of adding the dipeptidyl peptidase-4 inhibitor alogliptin to metformin therapy in patients with type 2 diabetes inadequately controlled with metformin monotherapy: a multicentre, randomised, double-blind, placebo-controlled study. International journal of clinical practice 2009;63:46-55.

169. Nowicki M, Rychlik I, Haller H, Warren ML, Suchower L, Gause-Nilsson I. Saxagliptin improves glycaemic control and is well tolerated in patients with type 2 diabetes mellitus and renal impairment. Diabetes, obesity & metabolism 2011;13:523-32.

170. Owens DR, Swallow R, Dugi KA, Woerle HJ. Efficacy and safety of linagliptin in persons with type 2 diabetes inadequately controlled by a combination of metformin and sulphonylurea: a 24-week randomized study. Diabetic medicine : a journal of the British Diabetic Association 2011;28:1352-61.

171. Pan C, Xing X, Han P, et al. Efficacy and tolerability of vildagliptin as add-on therapy to metformin in Chinese patients with type 2 diabetes mellitus. Diabetes, obesity & metabolism 2012;14:737-44.

172. Pratley RE, Reusch JE, Fleck PR, Wilson CA, Mekki Q. Efficacy and safety of the dipeptidyl peptidase-4 inhibitor alogliptin added to pioglitazone in patients with type 2 diabetes: a randomized, double-blind, placebo-controlled study. Current medical research and opinion 2009;25:2361-71.

173. Pratley RE, Kipnes MS, Fleck PR, Wilson C, Mekki Q. Efficacy and safety of the dipeptidyl peptidase-4 inhibitor alogliptin in patients with type 2 diabetes inadequately controlled by glyburide monotherapy. Diabetes, obesity & metabolism 2009;11:167-76.

174. Raz I, Chen Y, Wu M, et al. Efficacy and safety of sitagliptin added to ongoing metformin therapy in patients with type 2 diabetes. Current medical research and opinion 2008;24:537-50.

175. Rosenstock J, Brazg R, Andryuk PJ, Lu K, Stein P. Efficacy and safety of the dipeptidyl peptidase-4 inhibitor sitagliptin added to ongoing pioglitazone therapy in patients with type 2 diabetes: a 24-week, multicenter, randomized, double-blind, placebo-controlled, parallel-group study. Clinical therapeutics 2006;28:1556-68.

176. Ross SA, Rafeiro E, Meinicke T, Toorawa R, Weber-Born S, Woerle HJ. Efficacy and safety of linagliptin 2.5 mg twice daily versus 5 mg once daily in patients with type 2 diabetes inadequately controlled on metformin: a randomised, double-blind, placebo-controlled trial. Current medical research and opinion 2012;28:1465-74.

177. Scott R, Loeys T, Davies MJ, Engel SS. Efficacy and safety of sitagliptin when added to ongoing metformin therapy in patients with type 2 diabetes. Diabetes, obesity & metabolism 2008;10:959-69.

178. Seino Y, Fujita T, Hiroi S, Hirayama M, Kaku K. Alogliptin plus voglibose in Japanese patients with type 2 diabetes: a randomized, double-blind, placebo-controlled trial with an open-label, long-term extension. Current medical research and opinion 2011;27 Suppl 3:21-9.

179. Seino Y, Miyata Y, Hiroi S, Hirayama M, Kaku K. Efficacy and safety of alogliptin added to metformin in Japanese patients with type 2 diabetes: a randomized, double-blind, placebo-controlled trial with an open-label, long-term extension study. Diabetes, obesity & metabolism 2012;14:927-36.

180. Strain WD, Lukashevich V, Kothny W, Hoellinger MJ, Paldanius PM. Individualised treatment targets for elderly patients with type 2 diabetes using vildagliptin add-on or lone therapy (INTERVAL): a 24 week, randomised, double-blind, placebo-controlled study. Lancet 2013;382:409-16.

181. Taskinen MR, Rosenstock J, Tamminen I, et al. Safety and efficacy of linagliptin as add-on therapy to metformin in patients with type 2 diabetes: a randomized, double-blind, placebo-controlled study. Diabetes, obesity & metabolism 2011;13:65-74.

182. Vilsboll T, Rosenstock J, Yki-Jarvinen H, et al. Efficacy and safety of sitagliptin when added to insulin therapy in patients with type 2 diabetes. Diabetes, obesity & metabolism 2010;12:167-77.

183. Yang W, Pan CY, Tou C, Zhao J, Gause-Nilsson I. Efficacy and safety of saxagliptin added to metformin in Asian people with type 2 diabetes mellitus: a randomized controlled trial. Diabetes research and clinical practice 2011;94:217-24.

184. Yang W, Guan Y, Shentu Y, et al. The addition of sitagliptin to ongoing metformin therapy significantly improves glycemic control in Chinese patients with type 2 diabetes. Journal of diabetes 2012;4:227-37.

185. Yang W, Xing X, Lv X, et al. Vildagliptin added to sulfonylurea improves glycemic control without hypoglycemia and weight gain in Chinese patients with type 2 diabetes mellitus. Journal of diabetes 2015;7:174-81.

186. Bailey CJ, Iqbal N, T'Joen C, List JF. Dapagliflozin monotherapy in drug-naive patients with diabetes: a randomized-controlled trial of low-dose range. Diabetes, obesity & metabolism 2012;14:951-9.

187. Ferrannini E, Ramos SJ, Salsali A, Tang W, List JF. Dapagliflozin monotherapy in type 2 diabetic patients with inadequate glycemic control by diet and exercise: a randomized, double-blind, placebo-controlled, phase 3 trial. Diabetes care 2010;33:2217-24.

188. Ferrannini E, Seman L, Seewaldt-Becker E, Hantel S, Pinnetti S, Woerle HJ. A Phase IIb, randomized, placebo-controlled study of the SGLT2 inhibitor empagliflozin in patients with type 2 diabetes. Diabetes, obesity & metabolism 2013;15:721-8.

189. Fonseca VA, Ferrannini E, Wilding JP, et al. Active- and placebo-controlled dose-finding study to assess the efficacy, safety, and tolerability of multiple doses of ipragliflozin in patients with type 2 diabetes mellitus. Journal of diabetes and its complications 2013;27:268-73.

190. Inagaki N, Kondo K, Yoshinari T, Maruyama N, Susuta Y, Kuki H. Efficacy and safety of canagliflozin in Japanese patients with type 2 diabetes: a randomized, double-blind, placebo-controlled, 12-week study. Diabetes, obesity & metabolism 2013;15:1136-45.

191. Ji L, Ma J, Li H, et al. Dapagliflozin as monotherapy in drug-naive Asian patients with type 2 diabetes mellitus: a randomized, blinded, prospective phase III study. Clinical therapeutics 2014;36:84-100 e9.

192. List JF, Woo V, Morales E, Tang W, Fiedorek FT. Sodium-glucose cotransport inhibition with dapagliflozin in type 2 diabetes. Diabetes care 2009;32:650-7.

193. Stenlof K, Cefalu WT, Kim KA, et al. Efficacy and safety of canagliflozin monotherapy in subjects with type 2 diabetes mellitus inadequately controlled with diet and exercise. Diabetes, obesity & metabolism 2013;15:372-82.

194. Bailey CJ, Gross JL, Pieters A, Bastien A, List JF. Effect of dapagliflozin in patients with type 2 diabetes who have inadequate glycaemic control with metformin: a randomised, double-blind, placebo-controlled trial. Lancet 2010;375:2223-33.

195. Barnett AH, Mithal A, Manassie J, et al. Efficacy and safety of empagliflozin added to existing antidiabetes treatment in patients with type 2 diabetes and chronic kidney disease: a randomised, double-blind, placebo-controlled trial. The lancet Diabetes & endocrinology 2014;2:369-84.

196. Bolinder J, Ljunggren O, Kullberg J, et al. Effects of dapagliflozin on body weight, total fat mass, and regional adipose tissue distribution in patients with type 2 diabetes mellitus with inadequate glycemic control on metformin. The Journal of clinical endocrinology and metabolism 2012;97:1020-31.

197. Forst T, Guthrie R, Goldenberg R, et al. Efficacy and safety of canagliflozin over 52 weeks in patients with type 2 diabetes on background metformin and pioglitazone. Diabetes, obesity & metabolism 2014;16:467-77.

198. Haring HU, Merker L, Seewaldt-Becker E, et al. Empagliflozin as add-on to metformin plus sulfonylurea in patients with type 2 diabetes: a 24-week, randomized, double-blind, placebo-controlled trial. Diabetes care 2013;36:3396-404.

199. Haring HU, Merker L, Seewaldt-Becker E, et al. Empagliflozin as add-on to metformin in patients with type 2 diabetes: a 24-week, randomized, double-blind, placebo-controlled trial. Diabetes care 2014;37:1650-9.

200. Kovacs CS, Seshiah V, Swallow R, et al. Empagliflozin improves glycaemic and weight control as add-on therapy to pioglitazone or pioglitazone plus metformin in patients with type 2 diabetes: a 24-week, randomized, placebo-controlled trial. Diabetes, obesity & metabolism 2014;16:147-58.

201. Ljunggren O, Bolinder J, Johansson L, et al. Dapagliflozin has no effect on markers of bone formation and resorption or bone mineral density in patients with inadequately controlled type 2 diabetes mellitus on metformin. Diabetes, obesity & metabolism 2012;14:990-9.

202. Rosenstock J, Aggarwal N, Polidori D, et al. Dose-ranging effects of canagliflozin, a sodium-glucose cotransporter 2 inhibitor, as add-on to metformin in subjects with type 2 diabetes. Diabetes care 2012;35:1232-8.

203. Rosenstock J, Jelaska A, Frappin G, et al. Improved glucose control with weight loss, lower insulin doses, and no increased hypoglycemia with empagliflozin added to titrated multiple daily injections of insulin in obese inadequately controlled type 2 diabetes. Diabetes care 2014;37:1815-23.

204. Strojek K, Yoon KH, Hruba V, Elze M, Langkilde AM, Parikh S. Effect of dapagliflozin in patients with type 2 diabetes who have inadequate glycaemic control with glimepiride: a randomized, 24-week, double-blind, placebo-controlled trial. Diabetes, obesity & metabolism 2011;13:928-38.

205. Wilding JP, Norwood P, T'Joen C, Bastien A, List JF, Fiedorek FT. A study of dapagliflozin in patients with type 2 diabetes receiving high doses of insulin plus insulin sensitizers: applicability of a novel insulin-independent treatment. Diabetes care 2009;32:1656-62.

206. Wilding JP, Woo V, Soler NG, et al. Long-term efficacy of dapagliflozin in patients with type 2 diabetes mellitus receiving high doses of insulin: a randomized trial. Annals of internal medicine 2012;156:405-15.

207. Wilding JP, Ferrannini E, Fonseca VA, Wilpshaar W, Dhanjal P, Houzer A. Efficacy and safety of ipragliflozin in patients with type 2 diabetes inadequately controlled on metformin: a dose-finding study. Diabetes, obesity & metabolism 2013;15:403-9.

208. Yale JF, Bakris G, Cariou B, et al. Efficacy and safety of canagliflozin in subjects with type 2 diabetes and chronic kidney disease. Diabetes, obesity & metabolism 2013;15:463-73.

209. Fonseca VA, Alvarado-Ruiz R, Raccah D, Boka G, Miossec P, Gerich JE. Efficacy and safety of the once-daily GLP-1 receptor agonist lixisenatide in monotherapy: a randomized, double-blind, placebo-controlled trial in patients with type 2 diabetes (GetGoal-Mono). Diabetes care 2012;35:1225-31.

210. Madsbad S, Schmitz O, Ranstam J, Jakobsen G, Matthews DR. Improved glycemic control with no weight increase in patients with type 2 diabetes after once-daily treatment with the long-acting glucagon-like peptide 1 analog liraglutide (NN2211): a 12-week, double-blind, randomized, controlled trial. Diabetes care 2004;27:1335-42.

211. Moretto TJ, Milton DR, Ridge TD, et al. Efficacy and tolerability of exenatide monotherapy over 24 weeks in antidiabetic drug-naive patients with type 2 diabetes: a randomized, double-blind, placebo-controlled, parallel-group study. Clinical therapeutics 2008;30:1448-60.

212. Seino Y, Rasmussen MF, Zdravkovic M, Kaku K. Dose-dependent improvement in glycemia with once-daily liraglutide without hypoglycemia or weight gain: A double-blind, randomized, controlled trial in Japanese patients with type 2 diabetes. Diabetes research and clinical practice 2008;81:161-8.

213. Vilsboll T, Zdravkovic M, Le-Thi T, et al. Liraglutide, a long-acting human glucagon-like peptide-1 analog, given as monotherapy significantly improves glycemic control and lowers body weight without risk of hypoglycemia in patients with type 2 diabetes. Diabetes care 2007;30:1608-10.

214. Vilsboll T, Brock B, Perrild H, et al. Liraglutide, a once-daily human GLP-1 analogue, improves pancreatic B-cell function and arginine-stimulated insulin secretion during hyperglycaemia in patients with Type 2 diabetes mellitus. Diabetic medicine : a journal of the British Diabetic Association 2008;25:152-6.

215. Apovian CM, Bergenstal RM, Cuddihy RM, et al. Effects of exenatide combined with lifestyle modification in patients with type 2 diabetes. The American journal of medicine 2010;123:468 e9-17.

216. Buse JB, Henry RR, Han J, Kim DD, Fineman MS, Baron AD. Effects of exenatide (exendin-4) on glycemic control over 30 weeks in sulfonylurea-treated patients with type 2 diabetes. Diabetes care 2004;27:2628-35.

217. DeFronzo RA, Ratner RE, Han J, Kim DD, Fineman MS, Baron AD. Effects of exenatide (exendin-4) on glycemic control and weight over 30 weeks in metformin-treated patients with type 2 diabetes. Diabetes care 2005;28:1092-100.

218. Gao Y, Yoon KH, Chuang LM, et al. Efficacy and safety of exenatide in patients of Asian descent with type 2 diabetes inadequately controlled with metformin or metformin and a sulphonylurea. Diabetes research and clinical practice 2009;83:69-76.

219. Kadowaki T, Namba M, Yamamura A, Sowa H, Wolka AM, Brodows RG. Exenatide exhibits dose-dependent effects on glycemic control over 12 weeks in Japanese patients with suboptimally controlled type 2 diabetes. Endocrine journal 2009;56:415-24.

220. Kaku K, Rasmussen MF, Clauson P, Seino Y. Improved glycaemic control with minimal hypoglycaemia and no weight change with the once-daily human glucagon-like peptide-1 analogue liraglutide as add-on to sulphonylurea in Japanese patients with type 2 diabetes. Diabetes, obesity & metabolism 2010;12:341-7.

221. Kendall DM, Riddle MC, Rosenstock J, et al. Effects of exenatide (exendin-4) on glycemic control over 30 weeks in patients with type 2 diabetes treated with metformin and a sulfonylurea. Diabetes care 2005;28:1083-91.

222. Kim D, MacConell L, Zhuang D, et al. Effects of once-weekly dosing of a long-acting release formulation of exenatide on glucose control and body weight in subjects with type 2 diabetes. Diabetes care 2007;30:1487-93.

223. Ratner RE, Rosenstock J, Boka G. Dose-dependent effects of the once-daily GLP-1 receptor agonist lixisenatide in patients with Type 2 diabetes inadequately controlled with metformin: a randomized, double-blind, placebo-controlled trial. Diabetic medicine : a journal of the British Diabetic Association 2010;27:1024-32.

224. Russell-Jones D, Vaag A, Schmitz O, et al. Liraglutide vs insulin glargine and placebo in combination with metformin and sulfonylurea therapy in type 2 diabetes mellitus (LEAD-5 met+SU): a randomised controlled trial. Diabetologia 2009;52:2046-55.

225. Umpierrez GE, Blevins T, Rosenstock J, Cheng C, Anderson JH, Bastyr EJ, 3rd. The effects of LY2189265, a long-acting glucagon-like peptide-1 analogue, in a randomized, placebo-controlled, double-blind study of overweight/obese patients with type 2 diabetes: the EGO study. Diabetes, obesity & metabolism 2011;13:418-25.

226. Zinman B, Hoogwerf BJ, Duran Garcia S, et al. The effect of adding exenatide to a thiazolidinedione in suboptimally controlled type 2 diabetes: a randomized trial. Annals of internal medicine 2007;146:477-85.

227. Zinman B, Gerich J, Buse JB, et al. Efficacy and safety of the human glucagon-like peptide-1 analog liraglutide in combination with metformin and thiazolidinedione in patients with type 2 diabetes (LEAD-4 Met+TZD). Diabetes care 2009;32:1224-30.
